# The SEMA3F-NRP1/NRP2 axis is a key factor in the acquisition of invasive traits in in situ breast ductal carcinoma

**DOI:** 10.1186/s13058-024-01871-0

**Published:** 2024-08-13

**Authors:** Núria Moragas, Patricia Fernandez-Nogueira, Leire Recalde-Percaz, Jamie L. Inman, Anna López-Plana, Helga Bergholtz, Aleix Noguera-Castells, Pedro J. del Burgo, Xieng Chen, Therese Sorlie, Pere Gascón, Paloma Bragado, Mina Bissell, Neus Carbó, Gemma Fuster

**Affiliations:** 1 Department of Biochemistry and Molecular Biomedicine, Universitat de Barcelona (UB), 08028 Barcelona, Spain; 2 Institute of Biomedicine of the Universitat de Barcelona (IBUB), Barcelona, Spain; 3 Department of Biomedicine, School of Medicine, Universitat de Barcelona (UB), 08036 Barcelona, Spain; 4 Biological Systems and Engineering Division, Lawrence Berkeley National Laboratory, 1 Cyclotron Rd., Berkeley, CA 94720 USA; 5 Department of Cancer Genetics, Institute for Cancer Research, Oslo University Hospital, 0450 Oslo, Norway; 6 Cancer Epigenetics Group, Josep Carreras Leukaemia Research Institute (IJC), Barcelona, Catalonia Spain; 7 Centro de Investigacion Biomedica en Red Cancer (CIBERONC), Madrid, Spain; 8 Department of Biosciences, Faculty of Science, Technology and Engineering, University of Vic - Central University of Catalonia (UVic-UCC), Vic, Barcelona, Catalonia Spain; 9 Department of Biochemistry and Molecular Biology, Faculty of Pharmacy, Universidad Complutense de Madrid, Health Research Institute of the Hospital Clínico San Carlos, 28040 Madrid, Spain; 10 Tissue Repair and Regeneration Laboratory (TR2Lab), Institute of Research and Innovation of Life Sciences and Health, Catalunya Central (IRIS-CC), UVIC-UCC, Vic, Spain

**Keywords:** DCIS, Breast cancer, Neural mediator, Semaphorin 3F, Axonal guidance molecule, IDC

## Abstract

**Background:**

A better understanding of ductal carcinoma in situ (DCIS) is urgently needed to identify these preinvasive lesions as distinct clinical entities. Semaphorin 3F (SEMA3F) is a soluble axonal guidance molecule, and its coreceptors Neuropilin 1 (NRP1) and NRP2 are strongly expressed in invasive epithelial BC cells.

**Methods:**

We utilized two cell line models to represent the progression from a healthy state to the mild-aggressive or ductal carcinoma in situ (DCIS) stage and, ultimately, to invasive cell lines. Additionally, we employed in vivo models and conducted analyses on patient databases to ensure the translational relevance of our results.

**Results:**

We revealed SEMA3F as a promoter of invasion during the DCIS-to-invasive ductal carcinoma transition in breast cancer (BC) through the action of NRP1 and NRP2. In epithelial cells, SEMA3F activates epithelialmesenchymal transition, whereas it promotes extracellular matrix degradation and basal membrane and myoepithelial cell layer breakdown.

**Conclusions:**

Together with our patient database data, these proof-of-concept results reveal new SEMA3F-mediated mechanisms occurring in the most common preinvasive BC lesion, DCIS, and represent potent and direct activation of its transition to invasion. Moreover, and of clinical and therapeutic relevance, the effects of SEMA3F can be blocked directly through its coreceptors, thus preventing invasion and keeping DCIS lesions in the preinvasive state.

**Supplementary Information:**

The online version contains supplementary material available at 10.1186/s13058-024-01871-0.

## Background

Among all new cancer cases, breast cancer (BC) has the highest incidence and is the second leading cause of cancer death among women. The most common noninvasive breast lesion is ductal carcinoma in situ (DCIS), which currently represents 20–25% of all new BC cases diagnosed and accounts for more than 40,000 predicted new cases in the USA in 2020 [1]. DCIS constitutes a collection of heterogeneous lesions characterized by a wide diversity of risk factors for progression [2]. In fact, up to 40% of patients will rapidly progress to invasive ductal carcinoma (IDC) if untreated or undertreated, whereas most cases remain virtually unaltered for up to 5–20 years or even do not evolve at all [3]. The widespread utilization of screening mammography has led to an increase in diagnosed DCIS cases, which can be readily treated with surgery and radiotherapy. In contrast, the frequency of IDC remains stable, indicating that some DCIS cases will be overtreated without any therapeutic benefit [2]. Overtreatment of DCIS patients is an underlying reality and must be addressed since it clearly worsens patients’ quality of life, including physical and psychological aspects [4]. Considering this, both DCIS risk factors and the diagnostic tools used must be taken into consideration. On the one hand, DCIS risk factors include those that are determinants of IDC, such as sex, older age, obesity, high breast density, BC family history, nulliparity or late age at the first pregnancy, and hormone therapy after menopause [2]. On the other hand, histological grade and margin state are some of the diagnostic factors used to determine the probability of a preinvasive lesion evolving into invasive cancer. However, the efficiency and robustness of risk prediction and diagnosis are insufficient for discerning the potential risk of a given DCIS lesion becoming an IDC.

Little is known about the molecular and cellular mechanisms underlying the eventual progression from DCIS to IDC in BC patients. In this context, cancer cells and microenvironmental interactions play crucial roles in the DCIS transition to IDC by stimulating invasion and blocking immune surveillance [5, 6]. Soluble factors, extracellular matrix (ECM) components and ECM organization, and different cell types, including immune and myoepithelial cells, are important microenvironmental factors and are considered essential elements for the regulation of eventual progression [7, 8]. Myoepithelial cells constitute one of the most important microenvironmental cells involved in the DCIS transition to IDC since they surround the duct and are responsible for basement membrane secretion. Hence, the loss of myoepithelial function is associated with the progression of DCIS and the acquisition of an invasive character [9]*.*

In the context of the influence of the tumor microenvironment on cancer, little is known about the role of the nervous system. Independent studies performed in the prostate [10], stomach [11] and, recently, in BC [12, 13] have shown that the peripheral autonomic nervous system is a fundamental actor in how the tumor microenvironment regulates cancer progression [10, 11]. The underlying mechanism involves stress response factors such as hormones and neuronal factors, which stimulate cancer cells through specific receptors [14]. In addition, neuronal factors may stimulate not only cancer cells but also key stromal cells, such as myoepithelial cells and fibroblasts, during the transition from DCIS to invasive disease [15]. Taken together, these data strongly suggest that crosstalk between cancer cells, stromal cells, and the nervous system through innervation and, most importantly, through soluble factors is crucial in oncology [15]. Concerning this, our group has identified several neural-related genes that are upregulated in different BC subtypes, and their expression correlates with prognosis [16, 17]. Among these neural factors, NRP2 is related to poor prognosis in IDC patients, especially in those with the basal-like BC subtype [16]; interestingly, NRP2 has also been described as an important contributor to mammary gland branching and development [18]. However, the impact of several of these neural factors on the transition from DCIS to IDC, either directly or through altering the phenotype of cancer or microenvironmental cells, has not been elucidated.

Semaphorin 3F (SEMA3F) is one of the ligands of NRP. SEMA3F was first described as a repulsive axonal guidance signal [19] and is involved in cancer-related vascular and tumor biology as a PI3K-AKT-mTOR pathway inhibitor [20, 21]. SEMA3F signals through NRP1 and NRP2 coreceptors, with high affinity for NRP2, that form heteroprotein complexes with the plexin (PLXN) family of receptors, mainly the PLXN A type, and therefore transduce signals intracellularly [22]. Moreover, it has been described that SEMA3-NRPs-PLXNs complexes are hexamers that include a NRPs homodimer or heterodimer, indicating that NRP1 and NRP2 could be present in one of these possible complexes of SEMA3F signal transduction [23]. PLXNs possess a cytoplasmic domain with GTPase-activating protein (GAP) activity, which has the ability to interact with Ras and Rho family small GTPases to transduce the signal downstream, ultimately leading to higher proliferation and migration capacities [24]. Interestingly, SEMA3F through NRP2 has been described as a promoter of postnatal mammary gland morphogenesis [25]. Although SEMA3F has been extensively reported to act as a tumor suppressor element by inhibiting cell migration in the context of metastasis [26–29], two recent publications point to a prometastatic role of SEMA3F in hepatocarcinoma [30, 31], thus suggesting that SEMA3F is a suitable poor prognosis marker. Another recent publication related high SEMA3F expression in cancer tissue, specifically in BC tissue, to that in contiguous normal tissue, and the authors noted the possibility that SEMA3F may be associated with poor prognosis in patients with the HER2 + BC subtype [32].

In this scenario, our hypothesis is that SEMA3F and its receptors NRP1 and NRP2 could contribute to DCIS progression to IDC, affecting cancer epithelial cells. Understanding how this occurs could help identify new therapeutic targets and relevant biomarkers for patient clinical management in the early stages of BC.

Our data provide clear evidence that SEMA3F and its receptors NRP1 and NRP2, which were previously used in the prognosis of invasive BC subtypes, also participate in fostering DCIS to IDC progression through the induction of an EMT process in cancer cells and consequently enhancing migration and invasion. Our study identified three new diagnostic and prognostic biomarkers of invasion risk (SEMA3F, NRP1 and NRP2) in DCIS patients and paves the way for new therapeutic strategies to maintain early BC at a noninvasive stage, a more affordable disease in clinical terms.

## Methods

### Cell lines

Human breast cell lines used were cultured in the following manner: **MCF10A (RRID:CVCL_0598)** (American Type Culture Collection (ATCC), VA, USA) and **MCF10A-T** (generated from the MCF10A, described below; Supplementary Fig. 1a–c) were cultured in Dulbecco’s modified Eagle’s medium nutrient mixture-F12 (DMEM-F12) (Gibco, Life Technologies, CA, USA), supplemented with 1% of L-GlutaMAX™ 200 mM (Gibco, Life Technologies CA, USA), 1% fungizone—penicillin—streptomycin mixture (Invitrogen), 5% of horse serum (HS; Gibco, Life Technologies CA, USA), 20 ng/mL epithelial growth factor (EGF) (Peprotech, NJ, USA), 0.5 μg/mL hydrocortisone (Sigma, MO, USA), 10 μg/mL insulin (Sigma, MO, USA) and 100 ng/mL cholera toxin (Sigma, MO, USA); **MCF10DCIS.com** cell line **(RRID:CVCL_5552)** (Asterand Inc, MI, USA) was cultured in DMEM-F12, supplemented with 1% of L-GlutaMAX™ 200 mM, 1% fungizone—penicillin—streptomycin mixture and 5% of HS; **SUM225 (RRID:CVCL_5593)** was cultured in DMEM-F12 supplemented with 1% fungizone—penicillin—streptomycin mixture, 5% FBS (Gibco, Life Technologies), 5 μg/mL insulin, 1 μg/ml hydrocortisone and 10 mM HEPES (Invitrogen, 15630). The **HMT-3522** mammary epithelial cells**,** which were obtained from the DCIS stage (S3A, S3B, S3C and T4), were obtained and cultured as previously described [33].

Using the above-described cell lines and as an experimental approach to study the transition from healthy breast epithelial cells and DCIS to IDC, two in vitro models were used. First, an in vitro model of commercial cell lines from MCF10DCIS.com and SUM225 (mild-aggressive cell lines in 3D culture and in vivo mimicking DCIS kindly provided by Dr. Fariba Behbod, Kansas University, USA) was used, and finally, the aggressive cell line MCF10A-T (invasive BC cell line) was produced in our laboratory (Supplementary Fig. 2a). In parallel, a second in vitro model was also used as a model of evolution from mild aggressive BC epithelial cells to invasive breast epithelial cell lines: S3A, S3B (mildly aggressive), S3C and T4 (more aggressive cell lines) (Supplementary Fig. 2b) [33].

All the cell lines used were cultured under a humidified atmosphere of 5% CO_2_ at 37 °C.

### Conditioned media experiments

For the experiments with conditioned media, 2 × 10^6^ cells were plated in 75 cm^3^ plates. After 24 h, fresh complete culture medium was added, and the cells were incubated for 24, 48 and 72 h. The supernatant was collected, centrifuged (150×*g* for 2 min) and filtered (0.45 μm) to discard unattached cells and cell debris. The conditioned medium (CM) was used directly or concentrated by centrifugal filter units 10 k (Amicon Ultra4—Millipore, UFC801096) to generate concentrated conditioned medium (CCM), which was subsequently stored at − 80 °C. CM and CMC were collected to carry out experimental treatments or protein detection in media by Western blot (WB).

### Generation of MCF10A-T cells: invasive BC model

MCF10A cells were transformed to generate MCF10A-T cells by sequential transfection of largeTgenomic and H-Ras V12 cells with the following plasmids: pBABE-zeo largeTgenomic was a gift from Bob Weinberg (Addgene plasmid # 1778; http://n2t.net/addgene:1778; RRID:Addgene_1778) [34], and pBABE puro H-Ras V12 was a gift from William Hahn (Addgene plasmid # 9051; http://n2t.net/addgene:9051; RRID:Addgene_9051). In parallel, MCF10A mock cells were generated by transfection of the following empty vectors: pBABE-zeo (pBABE-bleo), which was a gift from Hartmut Land and Jay Morgenstern and Bob Weinberg (Addgene plasmid # 1766; https://www.addgene.org/1766/; RRID:Addgene_1766) [35], and pBABE-puro, which was a gift from Hartmut Land and Jay Morgenstern and Bob Weinberg (Addgene plasmid # 1764; https://www.addgene.org/1764/; RRID:Addgene_1764)[35]. Briefly, MCF10A cells were seeded (0.33 × 10^6^ cells/cm^2^) in two 6-well plates and grown for 18–20 h. Then, the cells were infected with retroviral particles obtained from HEK293T cells diluted in media and in the presence of polybrene (8 µg/ml). Six-well plates were centrifuged at 950×*g* for 20 min to optimize virus infection. Twenty-four hours later, the media was changed, and 48 h later, selection with antibiotics started and was maintained for approximately 3 weeks (depending on control cell evolution). Once the selection process was complete, the MCF10A cells were fully transformed into MCF10A-T cells. The presence of the 2 transgenes was tested by WB and IF using antibodies against Sv40 large T and H-Ras (Supplementary Fig. 1a, b). 3D cell culture characterization of this cell line was also performed (Supplementary Fig. 1c), which revealed a more invasive 3D growth phenotype in MCF10A-T cells than in mock-transduced MCF10A control cells, which were clearly less rounded in shape [36]. Moreover, MCF10A-T cells exhibited a decrease in the expression of the myoepithelial cell markers CK14 and E-cadherin and an increase in the expression of vimentin, indicating that activation of the EMT program is intimately linked to invasion [37].

### Generation of MCF10DCIS BC cell lines overexpressing SEMA3F (MCF10DCIS_SEMA3F)

#### Plasmid constructs

The SEMA3F sequence was amplified via PCR from the pCMV6-XL5_SEMA3F plasmid (SC117510 NM_004186.2, OriGene) and subcloned and inserted into the BamHI and EcoRV sites of the pENTRA1A plasmid (ref. w186-1; Thermo Fisher).

This plasmid was subsequently used to generate lentiviral vectors via the Gateway cloning system (Invitrogen). This system allows the transfer of SEMA3F DNA fragments from the pENTRA1A plasmid into the entry clone pLenti CMV/TO Puro DEST 670-1 (Addgene). This recombination occurs specifically between the sequences attL1 and attL2, which are present in both vectors, owing to the enzyme LR clonase (Thermo Fisher).

Finally, to generate a stable MCF10DCIS.com cell line that overexpresses SEMA3F, cDNA was cloned and inserted into the pTRIPZ tetracycline-controlled expression (TET-on) vector (Dharmacon) and used as described by Onodera et al. [38]. MCF10DCIS.com cells were transduced with the Lv-SEMA3F and Lv-rtTA3 lentiviruses isolated from HEK-293T cells. The pLenti CMV/TO Puro DEST 670–1 plasmid with the SEMA3F fragment and the pLenti CMV rtTA3 Blast w756-1 plasmid, which encodes a tetracycline-repressible transactivator, were separately transfected into HEK-293T cells by the transfection reagent Lipofectamine (Invitrogen). After 24 h, the medium was changed, and the lentiviruses (Lv-SEMA3F and Lv-rtTA3) were collected daily for 2 days, passed through a 0.45 μm filter and concentrated using the reactive Lenti-X concentration (Takara). The MCF10DCIS.com cells were cotransduced with a 10^–4^ dilution of the concentrated lentiviruses Lv-SEMA3F and Lv-rtTA3, which contained puromycin and blasticidin resistance, respectively. Puromycin- and blasticidin-resistant cells were isolated and maintained in the presence of 1 μg/ml puromycin and 4 μg/ml blasticidin. SEMA3F gene activation was performed with doxycycline (a tetracycline analogue) at 1 μg/ml, as indicated in Supplementary Fig. 4a.

### Three-dimensional cell culture

On-top three-dimensional (3D) cultures of Matrigel™ Basement Membrane Matrix (BD Bioscience, 10429212) were made in MW24 plates, and 10^4^ epithelial cells/well were seeded in 500 μL of complete medium supplemented with 5% Matrigel in a Matrigel precoated MW24-well. The medium was changed three times per week, and 0.5 ml of medium was added to each well. 3D cultures were maintained for 7 to 14 days. In the cases where this was needed, treatment was started after 5 days. Briefly, 100 ng/ml of SEMA3F recombinant protein (RP-SEMA 3F) (R&D Systems, 3237-S3) or blocking antibodies against NRP1 or NRP2 at 1 µg/ml were added every day to the cultured cells until day 14 after seeding. At the end of the experiment, the 3D cultures were photographed in vivo for further analysis of the photos. Next, the Matrigel was removed via a series of washes with PBS-EDTA, and the 3D structures were fixed on slides using 4% PFA, as previously described [17, 39, 40]. Fixed 3D cultures were stored at − 20 °C until use.

These cultures were evaluated by quantifying several characteristics, including the number of structures, the size, and the percentage of spherical structures, analysed from images captured on the collecting day and analysed using the ImageJ program. Other characteristics analysed were the organization and polarization of the cells detected by fluorescence F-actin (phalloidin Alexa Fluor 488) and the integrity of the acinar structure determined by performing IF with laminin-1 in the 3D cultures.

### Downregulation of SMAD2 by siRNA

SMAD2 siRNA transfection was performed according to the manufacturer’s instructions. Briefly, MCF10DCIS cells were seeded (40,000 cells/ml) in antibiotic-free medium overnight. The next day, the transfection reagents DharmaFECT 1 (Horizon) and SMAD2 siRNA (Thermo Fisher, ID 107873) were diluted in serum-free and antibiotic-free media, respectively, for 5 min, after which both dilutions were combined. After incubating for 20 min at RT, the solution was diluted five times in antibiotic-free medium and added to the cells. After incubating for 48 to 72 h, RNA and protein were extracted.

### RNA extraction and RT‒PCR procedures

Total RNA from cells and tumors was isolated with TRItidy G reagent and quantified using a NanoDrop 3000 Spectrophotometer. For RT‒PCR, 1 μg of RNA was reverse transcribed using a High-Capacity cDNA Reverse Transcription Kit (Life Technologies) following the manufacturer’s protocol. For the determination of the gene expression of SEMA3F, NRP1, NRP2, PLXNA1, PLXNA2, PLXNA3, PLXNA4, PLXND1, e-cadherin, vimentin, n-cadherin, TWIST, SNAIL1, SNAIL2, fibronectin, CD10, CK14, CK19 and SMAD2, the NZYSpeedy qPCR probe master mix and Applied Biosystems TaqMan Gene Expression assay were used, which consisted of a FAM dye-labelled TaqMan probe and the corresponding unlabelled primers (summarized in Supplementary Table 2). The PCR mixture consisted of a 10 μl final volume of 0.5 μl of each assayed probe, 2.5 μl of master mix (TaqMan Universal PCR Master Mix, Applied Biosystems), 5 μl of H2O DEPC and 2 μl of cDNA (final concentration of 1 ng). PCR was performed on a 7500 Real Time PCR System (Applied Biosystems). Transcript levels were normalized to those of beta-actin, which was used as an endogenous control. The expression levels were analysed in triplicate and were calculated using the 2^−ΔΔCt^ method.

### Western blot

Protein extraction was performed with RIPA lysis buffer (5 mM Tris–HCl, pH 7.4; 1% Nonidet P-40; 0.25% sodium deoxycholate; 150 mM NaCl; 1 mM EDTA; 1 mM PMSF; 1 mM protease inhibitor; 1 mM Na3VO4; and 1 mM NaF) and was quantified with a Pierce BCA Protein Assay Kit (Thermo Fisher). For the protein analysis, equal amounts of protein from each sample were separated via SDS‒PAGE, transferred to polyvinylidene difluoride membranes, blocked with 5% PBS–milk and incubated overnight with primary antibodies (summarized in Suppl Table 3). After 24 h, the membranes were incubated for 1 h at room RT with HRP-conjugated secondary antibodies (summarized in Suppl Table 3). Protein bands were detected after incubation with enhanced chemiluminescence (ECL) (*Amersham ECL Western Blotting Detection Reagent* (GE Healthcare)), and images were acquired with an LAS 4000 (ImageQuant). The obtained results were quantified using the Multi Gauge V3 0 Software program (Fujifilm). All the values obtained were normalized to the control protein values (GAPDH, tubulin or total Ponceau lane staining in CM and CMC WB assays).

### Immunofluorescence

IF staining of 2D and 3D cell culture and paraffin-embedded tissue was performed as previously described [17, 39, 41]. In brief, the fixed cultures were blocked with 1 × IF Buffer (10 × buffer: 10 × PBS, 1% BSA, 2% Triton X-100, 0.5%), 10% normal goat serum (Sigma‒Aldrich, G9023) and 1% mouse blocking antibody F(ab′)2 Fragment Goat Anti-Mouse IgG from Jackson Immune Research, 115-006-072) in the case of 3D culture for 1 h and incubated overnight with a primary antibody (summarized in Supplementary Table 3). Before blocking, the paraffin-embedded tissue was deparaffinized, rehydrated and subjected to antigen retrieval. The next day, after washing, secondary antibodies were added (summarized in Supplementary Table 3), and the *nuclei* were counterstained with 2 μg/mL Hoechst dye (Life Technologies, CA, USA). Cell coverslips were mounted using ProLong® Gold Antifade Reagent (Life Technologies, CA, USA). 2D culture and paraffin-embedded tissue images were taken with a Leica SP2 fluorescence microscope, and 3D culture was obtained via confocal microscopy (Leica SP5).

### Invasion assay

The invasive capacity of the cells was assessed via a transwell invasion assay. Briefly, 75,000 MCF10DCIS cells/200 μl were seeded in a transwell cell culture insert (MCEP24H48, Millipore) with an 8 μm pore size precoated with 40 µg of Matrigel in serum-free media and placed on a 24-well cell culture plate. 10% horse serum was added to the bottom of the cell culture plate as a chemoattractant. The cell cultures were incubated at 37 °C for 24 h, after which the cells were fixed with 4% PFA and stained with crystal violet solution. Images were captured, and the area of the cells was quantified using ImageJ software.

### ECM degradation assay using DQ-collagen IV

The degradation capacity of epithelial cells for extracellular matrix components was evaluated via an in vitro proteolysis assay with living cells as previously described [42]. Briefly, 24-well plates were coated with 120 μg of the 25 μg/ml quenched fluorescent substrate DQ-collagen IV (D12052; Invitrogen) mixed with Matrigel. Cells were seeded on top of the coating in 500 μl of complete media and incubated for 72 h. Proteolysis of DQ-collagen IV was observed, images were captured with a Leica SP5 confocal microscope (Leica), and quantification was performed using the ImageJ program.

### In vivo chicken embryo model

For the chicken chorioallantoic membrane (CAM) model, premium specific pathogen-free (SPF), fertile, 9-day-incubated embryonated chicken eggs were used. MCF10DCIS cells (2·10^6^) diluted in PBS and Matrigel were inoculated on CAMs, the tumors were grown for 6 days [17, 43], and the tumor sizes were no longer than 1 cm in diameter. After chronic treatment with SEMA3F, the day after inoculation, the tumors were treated with RP-SEMA3F for 5 days. For the MCF10DCIS_SEMA3F cells, the cells were treated with doxycycline at 1 µg/ml 3 days before inoculation and every day during the in vivo experiment at 10 µg/ml. On day 6 after inoculation, the tumors were excised, weighed, measured, and immediately fixed in 4% formaldehyde for IF analysis, or the samples were processed for RNA extraction.

### In vivo mouse model samples

Samples from an in vivo mouse model generated from nude mice (RRID:IMSR_JAX:002019) orthotopically inoculated with 100,000 MCF10DCIS cells in PBS:Matrigel [17]. The animals were sacrificed, and the tumors were excised, fixed, and embedded in paraffin at 7 and 29 days after inoculation. It should be noted that on day 7, the tumors were DCIS structures, and on day 29, these structures exhibited IDC characteristics, as it has been previously demonstrated with the loss of p63 in the myoepithelial cell’s layer [17].

### Patient samples

The patient samples used were obtained from the biobank of the Hospital Clinic de Barcelona (IDBAPS) under the approval of the institutional ethics committee. The samples used were obtained from patients with DCIS or IDC with a DCIS component.

### Bioinformatics procedure/analysis of the database

The open databases “Gene Expression-Based Outcome for Breast Cancer” (GOBO) [44] and “Gene Expression Omnibus” (GEO) [45] were used to study SEMA3F expression in patients.

GOBO data were used to generate Kaplan‒Meier survival curves for BC patients derived from published microarray datasets in the NCBI GEO database. These data were subsequently used to analyse the relationship between the expression of SEMA3F and the expression of the NRP1 and NRP2 coreceptors and OS as an endpoint and 10-year censoring; these data included all breast cancer subtypes, histological grade and 3–5 groups, from low expression to high expression.

Two GEO studies, GSE33692 [37] and GSE26304 [46], were used. These two studies focused on genetic analysis between patients with pure DCIS and patients with invasive BC and studies of the transition from DCIS to IDC. The GSE33692 and GSE26304 datasets were used to evaluate the differential gene expression of SEMA3F and its coreceptors NRP1 and NRP2 between patients with pure DCIS and patients with IDC. In particular, GSE33692 includes 9 patients with DCIS and 10 patients with IDC, and GSE26304 includes 31 patients with pure DCIS, 36 patients with IDC and 42 patients with DCIS + IDC.

In addition, a gene set enrichment analysis (GSEA) [47] was applied to the GSE26304 dataset from various previously generated groups based on SEMA3F expression in the pure DCIS samples divided into high expression and low-expression groups. The results shown in this work are those that were significantly different between the two groups tested, and from these, those that were considered most important according to their role in the transition from DCIS to IDC were chosen.

The Dr. Therese Sørlie cohort consisted of 57 pure DCIS and 313 fresh-frozen IDC tumor samples [48] obtained from 3 different BC cohorts (“Uppsala” and “Oslo2” [46, 48, 49] and the third one (“Milan”) [48]). Normal breast tissue samples were obtained from noncancer female patients via core needle biopsy [50]. All samples were obtained with the approval of future biomarker research studies; therefore, this study complied with the Helsinki Declaration Principles and was approved by the institution’s internal review and ethics board (Approval Numbers: 2016/433 [Oslo, Norway], PG/U-25/01/2012–00001497 [Milan, Italy], 2005/118 [Uppsala, Sweden]; IRB00003099, Barcelona, Spain]). The results derived from this cohort were all obtained at Oslo University Hospital and generated by Dr. Therese Sørlie. mRNA expression and Pearson correlation analyses were also carried out.

The scores were obtained from Estimate, a method that infers immune and stromal infiltration in tumors using gene expression data, as previously published [51].

## Statistical analysis

The results obtained in this work were graphically represented and statistically analysed using the GraphPad Prism 7 program. Depending on the type of study, Student's t-test, Mann‒Whitney U test, one-way ANOVA, two-way ANOVA, Wilcoxon signed-rank test, or Pearson correlation test was used. Differences were considered significant when p values less than or equal to 0.05 were obtained. The p values are marked on the graphs by asterisks: *, **, ***, and **** corresponding to p values less than or equal to 0.05, 0.01, 0.001 and 0.0001, respectively.

## Results

### SEMA3F and its coreceptors NRP1 and NRP2 are upregulated during BC invasion

**Fig. 1:**
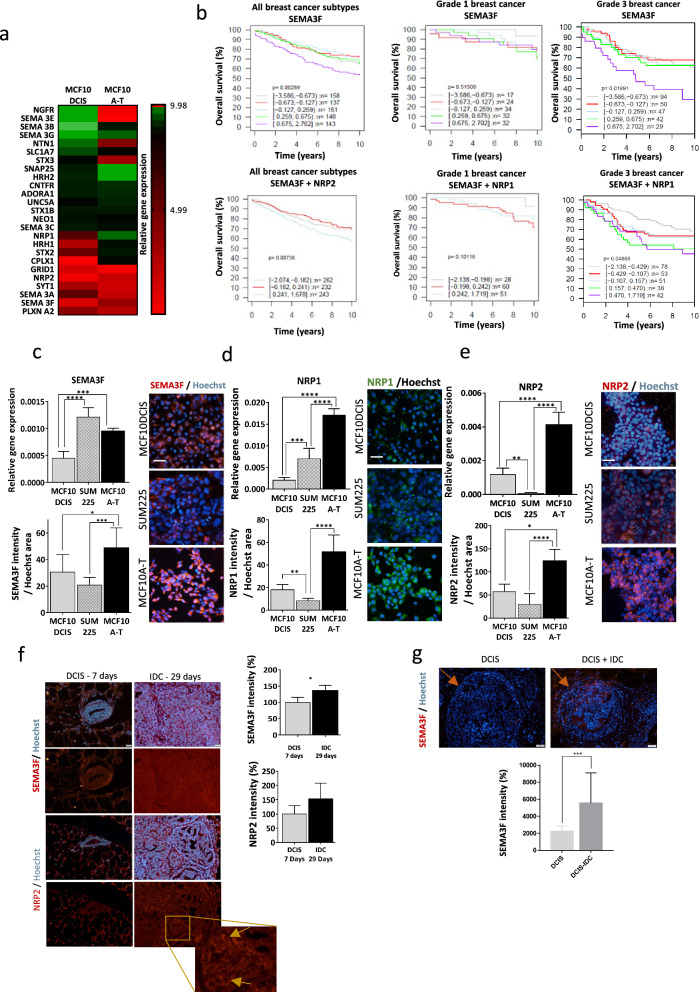
SEMA3F and its coreceptors NRP1 and NRP2 are upregulated during invasion. **a** Heatmap showing the gene expression analysis of the MCF10DCIS.com and invasive MCF10A-T-cell lines related to the nontumorigenic breast epithelial cell line (MCF10A). The color legend depicts an enrichment score [log2(expression/mean)], where relative gene expression ranges from 0 to 10. **b** Kaplan‒Meier curves for overall survival in a cohort of 1881 BC patients from the GOBO database based on SEMA3F, SEMA3F + NRP1 or SEMA3F + NRP2 expression in Grade 1 and 3 or all breast cancer types. Adapted from Györffy et al. [97]. Upper panels colors indicate from less expression to high expression of SEMA3F in this order: grey, red, light blue, green, purple. Bottom panels colors indicate from less expression to high expression of merged analysis from SEMA3F + NRP1 or SEMA3F + NRP2: grey, red and light blue. **c–e** Top left panels, analysis of the relative expression of *SEMA3F* **c**, *NRP1* **d** and *NRP2* **e** mRNAs in the MCF10DCIS, SUM225 and MCF10A-T-cell lines. Bottom left and right panels, representative IF images (right panels; scale bar: 40 µm) and mean fluorescence intensity (mfi) quantification (bottom left panels) for SEMA3F **c**, NRP1 **d** and NRP2 **e** in the in vitro DCIS-to-IDC transition model. The graphs represent the relative mean values ± S.E.M.ss; *P < 0.05, **P < 0.01, ***P < 0.001, ****P < 0.0001 for all the tumor cell lines according to one-way ANOVA and the Mann‒Whitney test. **f** Representative merged IF images (left panels; scale bar: 40 µm), and red individual channel corresponding to SEMA3F and NRP2 (including image amplification), and quantification of mfi expression (right panels) for SEMA3F and NRP2 in the in vivo DCIS-to-IDC transition mouse model. Yellow arrows indicate NRP2 membrane marker. The graphs represent the percentage (%) of mfi normalized to DCIS ± S.E.M. (n = 1, 5 inoculated mice per group); ns, nonsignificant; comparison of DCIS *vs* IDC by one-way ANOVA and the Mann‒Whitney test. **g** Representative IF images (left panels; scale bar: 20 µm) and mfi quantification (right panel) of SEMA3F expression in BC patients with DCIS or DCIS + IDC tumors. Orange arrows indicate the DCIS margin localization in both images. The graph represents mfi values ± S.E.M.; ***P < 0.001, comparing DCIS *vs* DCIS + IDC tumors by Student’s t test

Based on our previous findings that the prognosis of patients with different invasive BC subtypes is related to the differential expression of ten neurogenes (NTN1, HRH1, NRP2, STX1A, GRID1, NGFR, CNTFR, SLC17A7, ADORA1, APP) [16], we evaluated the expression of these genes and other related molecules, as well as some of their receptors/coreceptors and ligands (Supplementary Table 1), in our panel of BC cell lines. We constructed a model of evolution from healthy (MCF10A) to mildly aggressive (MCF10DCIS.com) and invasive (MCF10A-T) cell lines (Supplementary Fig. 1a). We also used another model of the DCIS cell line, SUM225 (Supplementary Fig. 1a), and a model of DCIS evolution to IDC, the HMT-3522 cell line series (Supplementary Fig. 1b). Both models fairly mimicked what happens in xenograft in vivo models and in patient DCIS (Supplementary Fig. 1c). From our gene expression analysis, those genes with changes greater than twofold in the MCF10DCIS.com and MCF10A-T cells relative to their expression in MCF10A cells were represented in a heatmap (Fig. 1a). A list of poor prognostic marker candidates was obtained from this heatmap, which included genes that were overexpressed in MCF10A-T cells relative to MCF10DCIS-treated cells, namely, NRP1, NRP2, HRH1, STX2, SNAP25, HRH2, SEMA3F and ADORA1 (Fig. 1a). Unexpectedly, these changes in the expression of SEMA3F and its coreceptors were not accompanied by significant increases in SEMA3F PLXN receptor expression (Fig. 1a). Through a GOBO database analysis [44] of the genes related to poor prognosis, we selected three genes related to these genes: SEMA3F and its coreceptors NRP1 and NRP2 (Fig. 1b). The expression of SEMA3F was negatively correlated with overall survival in invasive BC patients with all breast cancer subtypes and in grade 1 (less aggressive) and grade 3 tumors (the most aggressive ones), denoting the importance of SEMA3F expression as a marker of poor survival (Fig. 1b). Moreover, the combined expression of SEMA3F and NRP2 was inversely related to OS in all BC subtypes, and the combined expression of SEMA3F and NRP1 was negatively correlated with OS in patients with grade 1 and 3 tumors (Fig. 1b).

To further confirm SEMA3F expression in our cell line model from DCIS (MCF10DCIS.com and SUM225 cell lines) to invasive (MCF10A-T), both relative gene expression and protein levels were evaluated by qPCR and by WB and IF, respectively. The results obtained by IF (Fig. 1c) and WB (Supplementary Fig. 2a) confirmed the increase in the relative gene expression and protein level of SEMA3F in MCF10A-T cells compared to those in the healthy and mildly aggressive cell lines MCF10DCIS.com and SUM225. Additionally, the protein levels of the coreceptors NRP1 and NRP2 were also evaluated, confirming that both these two coreceptors can colocalize at perinuclear level and were upregulated in the invasive cell lines (Fig. 1d–e and Supplementary Fig. 2b). In parallel, the expression levels of SEMA3F were also verified in another BC evolution cell line series model that was previously validated [33]. In accordance with the MCF10DCIS.com and SUM225 transition models, the S3C and T4 cell lines, the most aggressive cell lines in the HMT-3522 cell line series, also showed an increase or a tendency toward an increase in SEMA3F expression (Supplementary Fig. 2c).

The increased expression levels of SEMA3F and NRP2 were confirmed using an in vivo mouse model of MCF10DCIS.com, which was previously described [17]; on day 7, MCF10DCIS.com was found to form DCIS, and on day 29, IDC BC or IDC was already present. The analysis of SEMA3F expression by IF revealed that on day 29 (IDC step) of the in vivo course, SEMA3F staining increased compared to that of the DCIS stage on day 7 (Fig. 1f, upper panel). We also tested the expression of the SEMA3F coreceptor NRP2 and found that its expression tended to increase on day 29 (Fig. 1f, bottom panel).

Finally, to determine the clinical relevance of SEMA3F in DCIS transition to IDC, DCIS tumors and DCIS + IDC tumors from BC patients were also stained for SEMA3F, which was greater in invasive patients than in DCIS patients (Fig. 1g); these findings indicate that SEMA3F could be a poor prognostic marker in DCIS patients.

### SEMA3F overexpression leads to a more invasive phenotype in MCF10DCIS.com cells

**Fig. 2:**
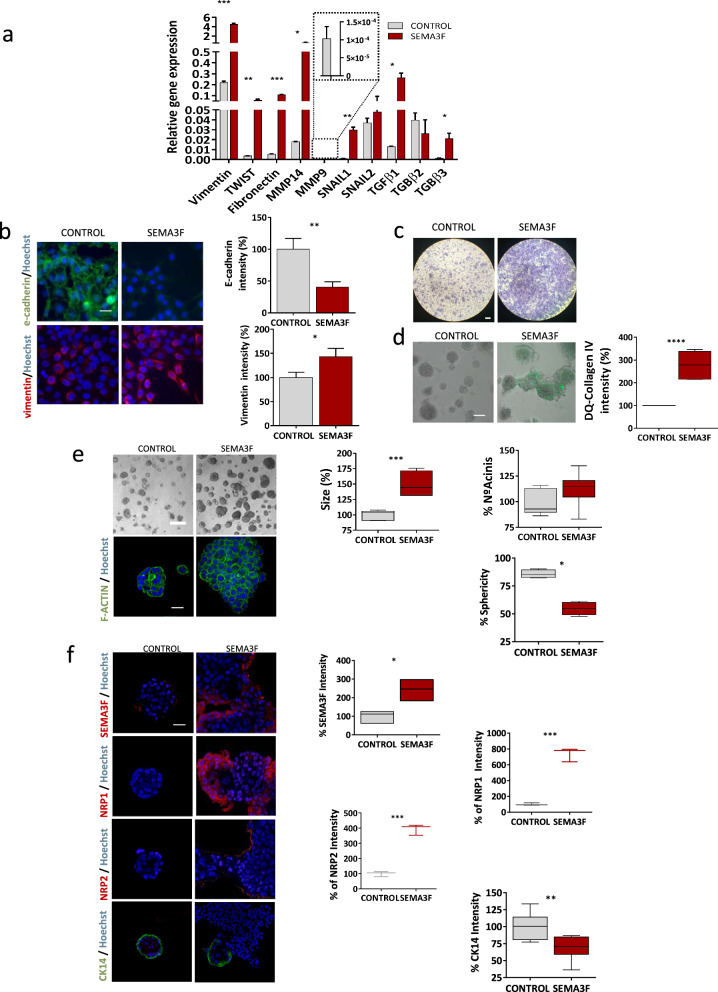
Effects of SEMA3F overexpression on MCF10DCIS cells in vitro. **a** Analysis of the relative expression of EMT-related genes in control or SEMA3F-overexpressing MCF10DCIS cells. **b** Representative IF images (left panels; scale bar: 20 µm) and the % of mfi quantification (right panels) for E-cadherin (upper panels) and vimentin (bottom panels) in SEMA3F-overexpressing MCF10DCIS cells. **c** Representative images of Transwell invasion assays of SEMA3F-overexpressing MCF10DCIS cells. Scale bar: 260 µm. **d** Representative DQ-collagen IV degradation assay images (left panels; scale bar: 50 µm) and the percentage of mfi-stained cells (right panels) for degraded DQ-collagen IV in SEMA3F-overexpressing MCF10DCIS cells. **e**, **f** Representative 3D phase contrast and IF images (e; scale bar = 200 µm for 3D images and scale bar = 20 µm for IF images) and the % of the number of acini, size, sphericity and SEMA3F, NRP1, NRP2 and CK14 mfi quantification (**f**) in SEMA3F-overexpressing MCF10DCIS cells. The graphs represent the mean values ± S.E.M.s. *P < 0.05, **P < 0.01, ***P < 0.001, ****P < 0.0001 for the comparison of control and SEMA3F-overexpressing MCF10DCIS cells determined by one-way ANOVA, an unpaired Student's t-test and the Mann‒Whitney U test

**Fig. 3:**
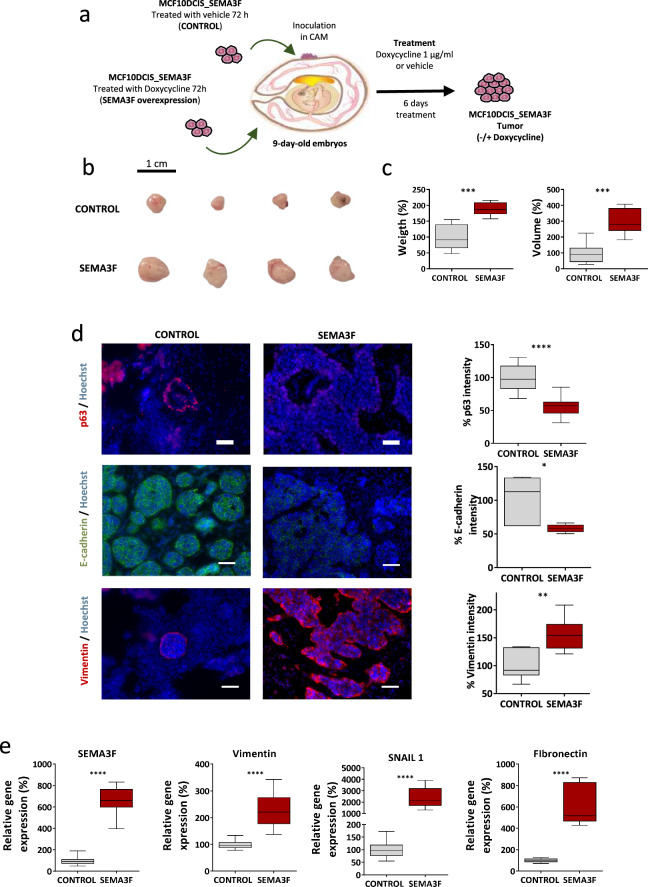
Effects of SEMA3F overexpression on MCF10DCIS cells in vivo. **a** Diagram of the CAM in vivo experiment using control or SEMA3F-overexpressing MCF10DCIS cells. A total of 2 × 10^6^ cells were inoculated in day-9-year-old chicken embryo CAM. Seventy-two hours later, the cells were pretreated with doxycycline (1 µg/mL) for SEMA3F overexpression. Tumors were treated with PBS (vehicle, control) or doxycycline (1 µg/mL) for 6 days. At the end of the experiment, the tumors were excised for further analyses. **b–c** Representative tumor images (**b**) and graphs representing the % of tumor weight (**c**; left panel) and tumor volume (**c**; right panel) at the end of the study. **d** Representative IF images (left panels; scale bar: 40 µm) and quantification of p63 (upper panels), E-cadherin (middle panels) and vimentin (bottom panels) expression in SEMA3F-overexpressing MCF10DCIS tumors. **e** Analysis of the relative mRNA expression of EMT-related genes in control or SEMA3F-overexpressing MCF10DCIS tumors. All the graphs represent the mean values ± S.E.M.s (n = 2, 5 inoculated eggs per group); ns, nonsignificant difference; *P < 0.05, ***P < 0.001, compared with control *or* SEMA3F-overexpressing tumors; one-way ANOVA and Mann‒Whitney U test were used

We showed that SEMA3F is overexpressed in invasive BC in in vitro and in vivo models and in BC patients (Fig. 1 and Supplementary Fig. 2). In the next step, we sought to test whether SEMA3F overexpression in MCF10DCIS.com cells leads to increased invasiveness in this mild-aggressive BC cell line. MCF10DCIS.com cells overexpressing SEMA3F (hereafter referred to as MCF10DCIS_SEMA3F) were generated by lentiviral infection, and SEMA3F expression was assessed via a tet-on system for doxycycline-inducible gene expression [38]. After 72 h of selection at 1 µg/ml doxycycline to achieve potent SEMA3F expression activation in MCF10DCIS_SEMA3F cells (Supplementary Fig. 3a), the effects of overexpression on DCIS progression to IDC were tested by in vitro and in vivo experiments (Figs. 2 and 3). We additionally observed that the expression of the SEMA3F coreceptors NRP1 and NRP2 was also upregulated in MCF10DCIS_SEMA3F (Supplementary Fig. 3b and c).

The epithelial-to-mesenchymal transition (EMT) is one of the most important processes in cancer progression and is particularly important for the acquisition of invasive traits [37]. The expression of EMT [52] markers and transcription factors was determined in MCF10DCIS_SEMA3F cells by qPCR and IF (Fig. 2). The expression of the vimentin, TWIST, fibronectin, SNAIL1 and SNAIL2 genes was induced in MCF10DCIS_SEMA3F cells compared with control cells (Fig. 2a). In addition, the expression of the invasiveness marker MMP14 and the TGFβ family member, a widely known inducer of EMT [53] (TGFβ1, TGFβ3) [54], also increased in MCF10DCIS_SEMA3F cells (Fig. 2a). Moreover, IF staining for vimentin and E-cadherin, the main EMT markers, revealed activation and downregulation, respectively, as expected and in agreement with our qPCR results (Fig. 2b).

The invasive ability of the SEMA3F-expressing cells was determined via transwell assays. MCF10DCIS_SEMA3F cells were able to invade the bottom side of the transwell membrane to a greater extent than the other cells (Fig. 2c). The invasive capacity of cancer cells partly relies on the proteolytic action of MMPs [55], which are certain extracellular matrix (ECM) components. Therefore, the presence of MMP2, MMP14 and TIMP2 was examined in the concentrated conditioned media (CCM) of MCF10DCIS_SEMA3F cells (Supplementary Fig. 3d). Consistently, SEMA3F overexpression in MCF10DCIS.com cells induced an increase in the protein level in all the patients evaluated (Supplementary Fig. 5d). To further corroborate the role of SEMA3F as a key invasiveness factor, real-time ECM degradation ability was monitored by a DQ-collagen IV assay (Fig. 2d). SEMA3F overexpression resulted in enhanced collagenase activity compared to that in the control group (Fig. 2d).

To gain deeper insight into the role of SEMA3F in DCIS BC biology, 3D cell culture, which recapitulates the main features of ECM-cancer cell crosstalk and mimics in vivo organization [56], was used. This was of utmost importance since the type and organization of 3D cell structures are linked to aggressiveness in BC cells, as has been previously described [36]. For this reason, we grew 3D-cultured MCF10DCIS.com cells overexpressing SEMA3F and then evaluated them by IF (Fig. 2e). SEMA3F overexpression affected the ability of MCF10DCIS.com cells to form acinar structures (Fig. 2e), as indicated by an increased size, fewer rounded 3D structures, and a decreased polarity, as indicated by the F-actin distribution. The localization of SEMA3F at the 3D acini edges (Fig. 2f) is highly reminiscent of what we previously observed in samples from patients (Fig. 1f). As expected, MCF10DCIS.com cells that overexpress SEMA3F exhibit a noticeable increase in both NRP1 and NRP2 expression in 3D cell cultures, which is more evident for NRP1 (Fig. 2f), and with a localization compatible with myoepithelial cell distribution in 3D BC cell cultures. Since MCF10DCIS.com cells can give rise to epithelial and myoepithelial cell lineages [57, 58] and because myoepithelial cells play a key role in DCIS progression to IDC [9]**,** we examined the status of myoepithelial cells in these 3D MCF10DCIS_SEMA3F structures (Fig. 2f). As expected, MCF10DCIS.com produced CK14+ myoepithelial cells surrounding the 3D growth of the cells (Fig. 2f). However, SEMA3F overexpression represents a challenge for the differentiation of MCF10DCIS.com cells into myoepithelial cells, as indicated by a decrease in the expression of the myoepithelial cell marker CK14 (Fig. 2f). In fact, CK14 + staining was present in only a few remaining rounded and nondisrupted *acini* (Fig. 2f). Interestingly, NRP1 was distributed similarly to CK14 and was strictly localized at the edges of acinar structures only in 3D cultures; moreover, the former had a rounded shape and were not disrupted (Fig. 2f). In contrast, NRP1 was absent in cells with an invasive capacity derived from the core of the 3D structures and invading the surrounding matrix (Fig. 2f). Similarly, NRP2 is expressed at 3D structure limits but is present in all 3D structures independent of shape (Fig. 2f). These results revealed that SEMA3F may enhance the expression of both NRP1 and NRP2 at the edge of the acinar structure, a region compatible with myoepithelial cell localization. Thus, SEMA3F overexpression could also affect the ability of MCF10DCIS.com cells to differentiate into the myoepithelial lineage, thereby inducing invasion through its effects on the myoepithelial cell layer.

In vivo studies to test SEMA3F overexpression in MCF10DCIS.com cells were also conducted in a chicken chorioallantoic membrane (CAM) model. Control and SEMA3F (MCF10DCIS-overexpressing SEMA3F) cells were inoculated into 9-day-old CAM chicken embryo eggs. SEMA3F-overexpressing cells were treated for 6 days with doxycycline to maintain SEMA3F overexpression (Fig. 3a). Control MCF10DCIS_SEMA3F cells were treated with vehicle. Seven days after inoculation, the tumors were excised, and the weight and volume were recorded (Fig. 3b, c). Overexpression of SEMA3F led to an increase in both tumor weight and tumor volume (Fig. 3b, c). Moreover, SEMA3F overexpression resulted in greater and less rounded growth and a reduction in the expression of the myoepithelial cell marker p63, which tended to be higher in the cancerous epithelial cells (Fig. 3d), as previously described in cells with the basal BC phenotype [59]. Similarly, changes in the distribution patterns of vimentin in the control tumors, which only labels myoepithelial cells, and in the tumors over-expressing SEMA3F, which is widely distributed in the tumor mass, have been observed (Fig. 3d). Additionally, MCF10DCIS_SEMA3F tumors also exhibited significant alterations toward EMT activation through a reduction in E-cadherin and an increase in vimentin (Fig. 3d), which additionally correlated with the qPCR vimentin, SNAIL1 and fibronectin results (Fig. 3e).

In summary, our results indicate that SEMA3F overexpression in MCF10DCIS.com leads to a more invasive phenotype and behavior in vitro and in vivo in this mild-aggressive cell line, suggesting that SEMA3F might promote DCIS progression to IDC.

### The SEMA3F-NRP1/NRP2 interaction promotes invasion

**Fig. 4:**
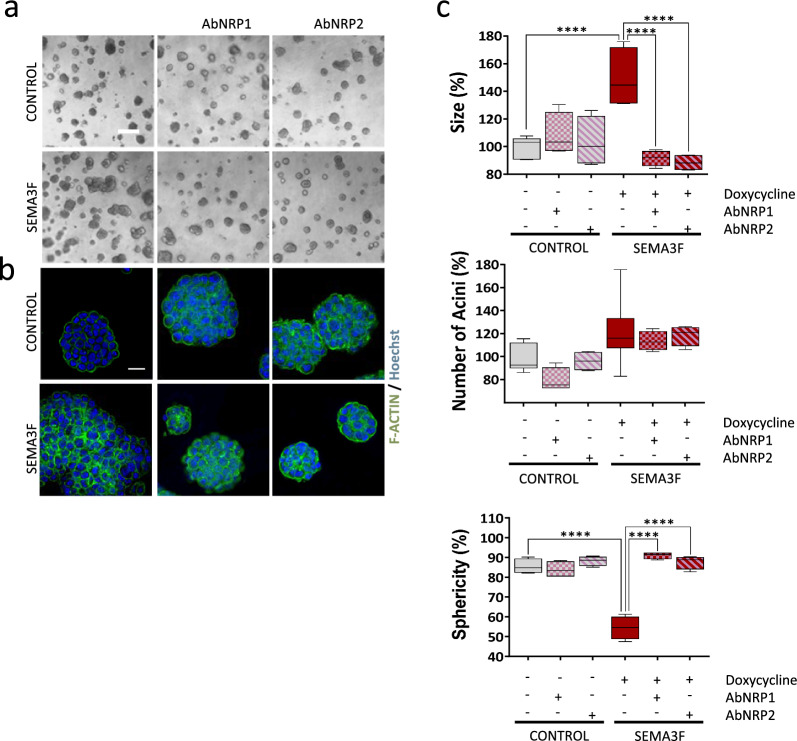
SEMA3F is a soluble proinvasive agent. **a** Representative 3D phase contrast images of SEMA3F-overexpressing MCF10DCIS cells in which NRP1 or NRP2 activity was blocked using monoclonal antibodies (1 µg/ml). Scale bar: 200 µm. **b** Representative IF images of 3D F-actin (phalloidin) colonies in A. Scale bar: 20 µm. **c** Quantification of the size, number of acini and sphericity of SEMA3F-overexpressing MCF10DCIS cells with NRP1 or NRP2 blocking antibodies. The graphs represent the mean % values ± S.E.M. ****P < 0.0001 for comparisons of control cells and SEMA3F-overexpressing MCF10DCIS cells; one-way ANOVA and the Mann‒Whitney U test were used

Since overexpression of SEMA3F induces the expression of NRP1 and NRP2 (Fig. 2g, h), we wondered whether SEMA3F was involved in signalling through these coreceptors. To test this, we blocked NRP2 and NRP1 activity in 3D cultures with monoclonal antibodies against these two co-receptors in SEMA3F-overexpressing cells. (Fig. 4a–c). Both NRP1 and NRP2 blockade clearly counteracted the effects of SEMA3F overexpression in 3D cell cultures of MCF10DCIS.com cells, reducing the size of acinar structures (counteraction of the effects of SEMA3F overexpression by 47.8% for AbNRP1 and by 51.1% for AbNRP2) and enhancing their rounded shape (counteraction of the effects of SEMA3F overexpression by 36.5% for AbNRP1 and by 33.2% for AbNRP2), similar to the control status (Fig. 4a–c). Remarkably, in SEMA3F-overexpressing cells, AbNRP2 treatment also restored 3D growth polarization, which is characteristic of control 3D growth, as detected by the distribution of F-actin (Fig. 4b), indicating that NRP2 could be a good target for inhibiting progression to an invasive phenotype. No significant effects were observed in the control groups treated with blocking antibodies against NRP1 or NRP2 (Fig. 4a–c). All these data reveal that SEMA3F is an invasive agent that acts in an autocrine and/or paracrine way, depending on the cellular source of secreted SEMA3F. These effects are mediated through the coreceptors NRP1 and NRP2, since the blockade of NRP1 and NRP2 in MCF10DCIS_SEMA3F cells indicates that secreted SEMA3F, which acts via NRP1 and/or NRP2 but not through the collateral changes that result in its overexpression, is the main factor responsible for the effects detected in cancer cells.

To better establish the pro-invasive role of SEMA3F, we analysed the effects of the recombinant SEMA3F protein (RP-SEMA3F) on mild aggressive BC cells both in vitro and in vivo to test whether SEMA3F per se can promote invasion in these cells, which could make it a good candidate for a poor prognosis in patients with DCIS BC.

**Fig. 5:**
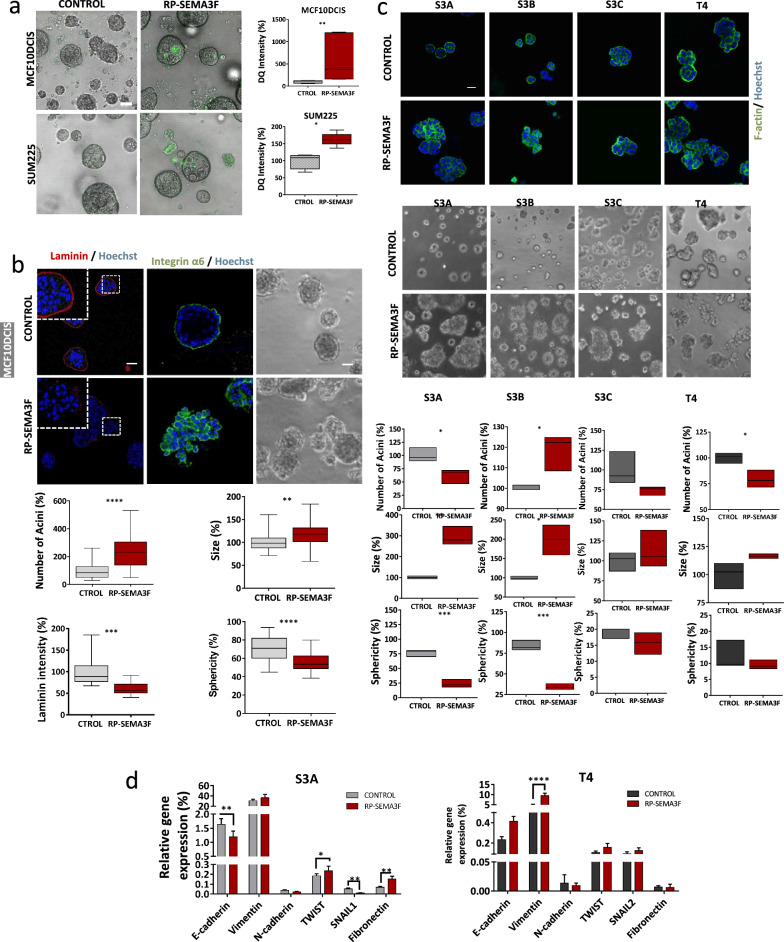
Effects of chronic SEMA3F treatment in DCIS and IDC model BC cell lines. **a** Representative DQ-collagen IV degradation assay images (left panels; scale bar: 50 µm) and the % of mfi quantification (right panels) for degraded DQ-collagen IV in MCF10DCIS (upper panels) and SUM225 (bottom panels) cells chronically treated with SEMA3F (RP-SEMA3F). **b** Representative 3D phase contrast (right images; scale bar: 50 µm) and IF images (left and middle images; scale bar = 20 µm for laminin-1, scale bar = 50 µm for integrin) and the percentages of acini, sizes, laminin-1 mfi quantification and sphericity (bottom panels) in MCF10DCIS cells chronically treated with SEMA3F (RP-SEMA3F). **c** Representative 3D IF (upper panels; scale bar: 50 µm) and phase contrast images (middle panels; scale bar: 20 µm) of HMT-3522 S1-derived cells chronically treated with SEMA3F (RP-SEMA3F). Lower panels, % of the number of acini, size and sphericity in HMT-3522 S1-derived cells chronically treated with SEMA3F (RP-SEMA3F). **d** Analysis of the relative mRNA expression of EMT-related genes in the less invasive and most invasive HMT-3522 S1-derived cells (control) or cells chronically treated with SEMA3F (RP-SEMA3F). The graphs represent the mean values ± S.E.M.s; ns, nonsignificant; *P < 0.05, **P < 0.01, ***P < 0.001, ****P < 0.0001 for comparisons of control cells and SEMA3F-treated cells; one-way ANOVA and the Mann‒Whitney U test were used

For this purpose, MCF10DCIS.com and SUM225 cells were chronically treated with 10 ng/ml RP-SEMA3F for two weeks (Supplementary Fig. 5a). WB analysis of MCF10DCIS.com cells treated with RP-SEMA3F revealed an increase in vimentin and the signalling proteins pSTAT3, pFAK, pSRC and β-catenin and a decrease in E-cadherin levels (Supplementary Fig. 5B). MCF10DCIS.com and SUM225 cells treated with SEMA3F also exhibited significantly greater collagenase activity than did the control cells (Fig. 5a). 3D cell cultures of MCF10DCIS.com and SUM225 cells, as well as cells from the HMT-3522 DCIS-to-IDC evolution model [33], were assessed under RP-SEMA3F treatment (Fig. 5b, c; Supplementary Fig. 4c). RP-SEMA3F affected 3D structures, particularly in MCF10DCIS.com cells, in the same way and direction as did SEMA3F overexpression, altering morphometric parameters, reducing basal polarity (as determined by integrin-α6 distribution Clone GoH3), and leading to decreased integrity of the basal membrane (as evaluated by IF of laminin-1) (Fig. 5b).

Among the mildly aggressive HMT-3522 cells (S3A and S3C), RP-SEMA3F treatment induced a significant increase in the 3D structure size and reduced sphericity and polarity, as determined by F-actin organization (Fig. 5c). qPCR analysis of the mRNA expression of EMT markers revealed that the EMT marker expression of mild-aggressive cell lines (S3A) significantly increased following SEMA3F treatment (Fig. 5d). These results point in the same direction as those obtained from the other mild-aggressive cell lines MCF10DCIS.com and SUM225. In contrast, the 3D growth of the most aggressive cell lines, S3C and T4, did not noticeably change, probably due to the invasive features already present in S3C and T4 cells per se [33]. However, T4, the most aggressive cell line, even exhibited a weakly significant increase in vimentin mRNA levels after SEMA3F treatment (Fig. 5d). Taken together, these results, from different DCIS cell line models, confirm the regulatory role of SEMA3F per se on invasion in 3D cell cultures.

### SEMA3F-TGFβ shows reciprocal positive interplay

**Fig. 6:**
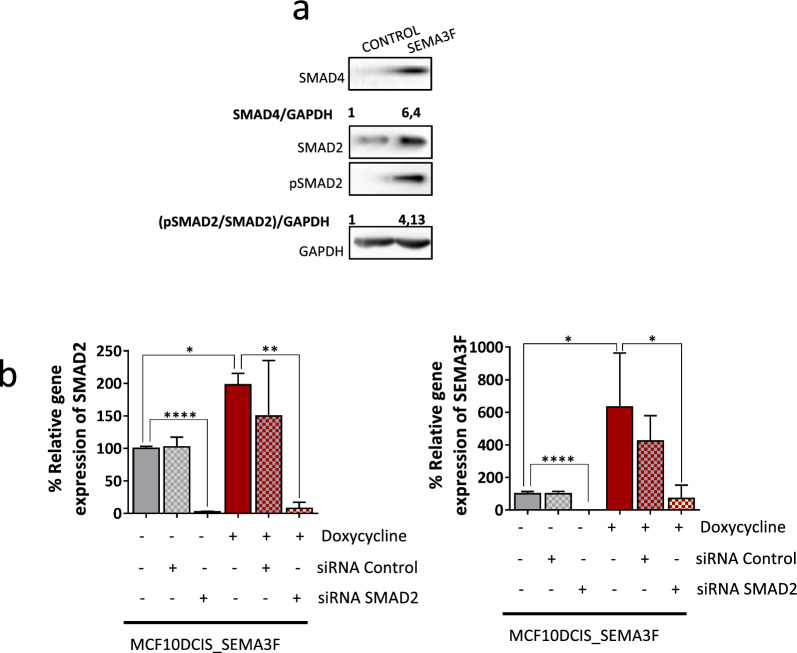
Mutual connection between the TGFβ signalling pathway and SEMA3F expression. **a** Representative western blot analysis of pSMAD2/SMAD2 and SMAD4 protein levels normalized to GAPDH in SEMA3F-overexpressing MCF10DCIS cells. Protein levels were quantified according to the nontreated control conditions. **b** Analysis of SMAD2 (left panel) and SEMA3F (right panel) relative mRNA expression in SEMA3F-overexpressing MCF10DCIS cells, in which SMAD2 was inhibited by a siRNA. The graphs represent the mean % values ± S.E.M.; *P < 0.05, **P < 0.01, ****P < 0.0001 for the comparison of the control *vs* SEMA3F-treated or SMAD2-inhibited cells determined by one-way ANOVA and the Mann‒Whitney U test

The responses to both SEMA3F treatment and overexpression resulted mainly in the activation of the EMT program (Fig. 2a–c, e; Fig. 5a, d; and Supplementary Fig. 4b, d). The TGFβ pathway, the most important signalling pathway involved in the activation of the EMT process [60], was also modulated (Fig. 2a). Therefore, we further analysed the relationship between SEMA3F and the TGFβ pathway. MCF10DCIS.com cells were treated with TGFβ1, one of the main ligands of the TGFβ signalling pathway, in time course experiments performed at different doses. In addition, the SEMA3F protein level increased in MCF10DCIS.com cells (Supplementary Fig. 5d), suggesting that TGFβ1 has a stimulatory effect on SEMA3F expression at 72 h and at higher doses tested. Interestingly, in MCF10DCIS cells overexpressing SEMA3F, pSMAD2, SMAD2, and SMAD4 protein levels were increased in comparison to those in control cells, indicating activation of the TGFβ signalling pathway by SEMA3F overexpression (Fig. 6a) [61].

To further investigate the mutual connection between the TGFβ pathway and SEMA3F, we investigated whether inhibition of TGFβ intracellular signalling affects SEMA3F expression. SMAD2 expression was blocked by siRNA in control and SEMA3F-overexpressing MCF10DCIS_SEMA3F cells (Fig. 6b, left panel), and a dramatic reduction in SEMA3F expression was observed in both the control and SEMA3F-overexpressing groups (Fig. 6b, right panel). Therefore, it can be concluded that there is a bidirectional relationship between the TGFβ signalling pathway and SEMA3F expression: TGFβ signalling activation promotes SEMA3F expression, while SEMA3F triggers the TGFβ pathway.

### High SEMA3F, stromal NRP1 and NRP2 expression as invasive DCIS prognosis markers in BC patients

To complement the first clinical analysis of SEMA3F, NRP1 and NRP2, which are poor prognostic markers for BC and DCIS, we further explored the clinical relevance of these proteins in DCIS and invasive BC tumors. Therefore, SEMA3F, NRP1 and NRP2 expression was correlated with molecular and clinical parameters in two datasets from the Gene Expression Omnibus (GEO) database [45] and in a BC patient cohort from the Institute for Cancer Research, Oslo (Norway) [48].

**Fig. 7:**
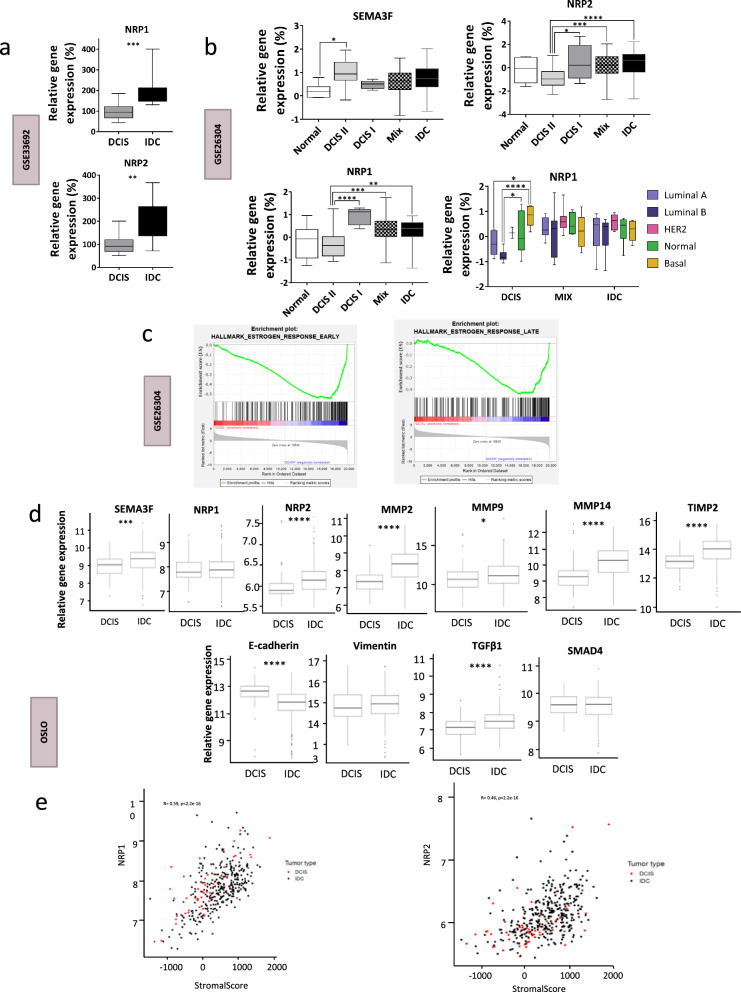
Bioinformatic analyses of SEMA3F and SEMA3F-related genes in BC patient databases. **a** Analysis of relative *NRP1* (upper panel) and *NRP2* (bottom panel) mRNA expression in DCIS and IDC patients using the GSE33692 dataset. **b** Analysis of the relative expression of the *SEMA3F* (upper left panel), *NRP2* (upper right panel) and *NRP1* (bottom left panel) mRNAs in healthy/normal samples and in several subtypes of DCIS and IDC BC patients using the GSE26304 database. Normal, DCIS I (more invasive), DCIS II (less invasive), mixed (DCIS + IDC), and IDC. The bottom right panel shows the relative *NRP1* mRNA expression in different BC subtypes, namely, luminal A, luminal B, HER2-enriched (HER2), normal-like and basal-like (basal) subtypes. **c** GSEA of a gene set panel of early (left panel) and late (right panel) estrogen response-related genes in low- and high-expressing SEMA3F DCIS BC patients from the GSE26304 database. **d** Analysis of *SEMA3F*, *NRP1*, *NRP2*, *MMP2*, *MMP9*, *MMP14*, *TIMP2*, *E-cadherin*, *vimentin*, *TGFβ1* and *SMAD4* mRNA expression in DCIS and IDC BC patients using Dr. Sørlie’s database in Oslo. **e** Correlations between stromal score and NRP1 (left panel) or NRP2 (right panel) in DCIS and IDC patients (Spearman’s correlation). The graphs represent the mean % values ± S.E.M.s; *P < 0.05, **P < 0.01, ***P < 0.001, ****P < 0.0001 for the comparison of DCIS versus IDC (**a**, **d**) and control versus BC (**b**) subtypes by one-way ANOVA, the Mann‒Whitney U test (**a**, **b**) or Wilcoxon’s test (**d**)

Data from two different studies (GSE33692 [37] and GSE26304 [46]), which focused on the genetic analysis of pure DCIS and IDCs in BC patients, were analysed. The GSE33692 dataset was used to evaluate the differential expression of SEMA3F and its coreceptors between pure DCIS and IDC [37] (Fig. 7a). We found that NRP1 and NRP2 were significantly upregulated in invasive tumors. Gene set enrichment analysis (GSEA) was performed on these data [37], and as expected and in accordance with our results, the EMT gene set was also upregulated in IDCs. The differential expression of SEMA3F, NRP1 and NRP2 was also evaluated in the GSE26304 dataset, which contains samples from normal, pure DCIS, DCIS + IDC, and IDC patients, with DCIS samples divided into DCIS type I (DCIS I, more prone to becoming invasive) and DCIS type II (DCIS II, less prone to becoming invasive) [62]. Although no clear difference in SEMA3F expression was detected between the DCIS and IDC groups, SEMA3F expression was significantly upregulated from the normal to DCIS II subgroup, and it was nearly significantly upregulated between the normal and both the DCIS I (more prone to becoming invasive) and invasive (IDC) groups (Fig. 7b; upper left panel). As already observed in the GSE33692 data analysis (Fig. 7a), in the GSE26304 cohort, NRP1 and NRP2 also exhibited increased expression in IDC samples versus DCIS II (the less invasive group) (Fig. 7b, upper right and bottom left panels). Moreover, high NRP1 expression correlated with the most aggressive BC subtype, basal-like tumors (Fig. 7b).

In addition, GSEA was carried out to compare high- and low-expression SEMA3F pure DCIS tumors, and within this analysis, we evaluated those gene sets related to the most prominent cancer features. High SEMA3F expression correlated with an enriched gene set related to the oestrogen response (Fig. 7c and Supplementary Table 4).

The expression of SEMA3F, NRP1, NRP2, EMT proteins and other genes related to invasion capacity (MMPs, TIMP2, E-cadherin, vimentin, TGFβ1 and SMAD4) was also analysed within the Oslo BC patient cohort composed of 57 pure DCIS and 313 IDC samples [48] (Fig. 7d, e). In accordance with our experimental results, SEMA3F and NRP2 were upregulated in the IDC samples compared to those in the pure DCIS sample (Fig. 7d); however, NRP1 did not significantly differ. The expression of EMT program activation markers, such as TGFβ1, MMP2, MMP14 and TIMP2, was also increased in the IDC samples (Fig. 7d). E-cadherin, an epithelial marker, was expressed at a reduced level in IDC samples, but no differences were observed in vimentin or SMAD4 (Fig. 7d). In addition, NRP1 and NRP2 were correlated with the stromal score in BC patients (Fig. 7e), suggesting that these two coreceptors could also be important for stromal cells, as well as for myoepithelial cells or fibroblasts, suggesting that SEMA3F likely exerts paracrine action and that alternative signals, such as those from VEGF, also function as coreceptors [63]. These data indicate that SEMA3F, NRP1 and NRP2 could be therapeutically targeted to prevent DCIS progression to IDC.

**Fig. 8:**
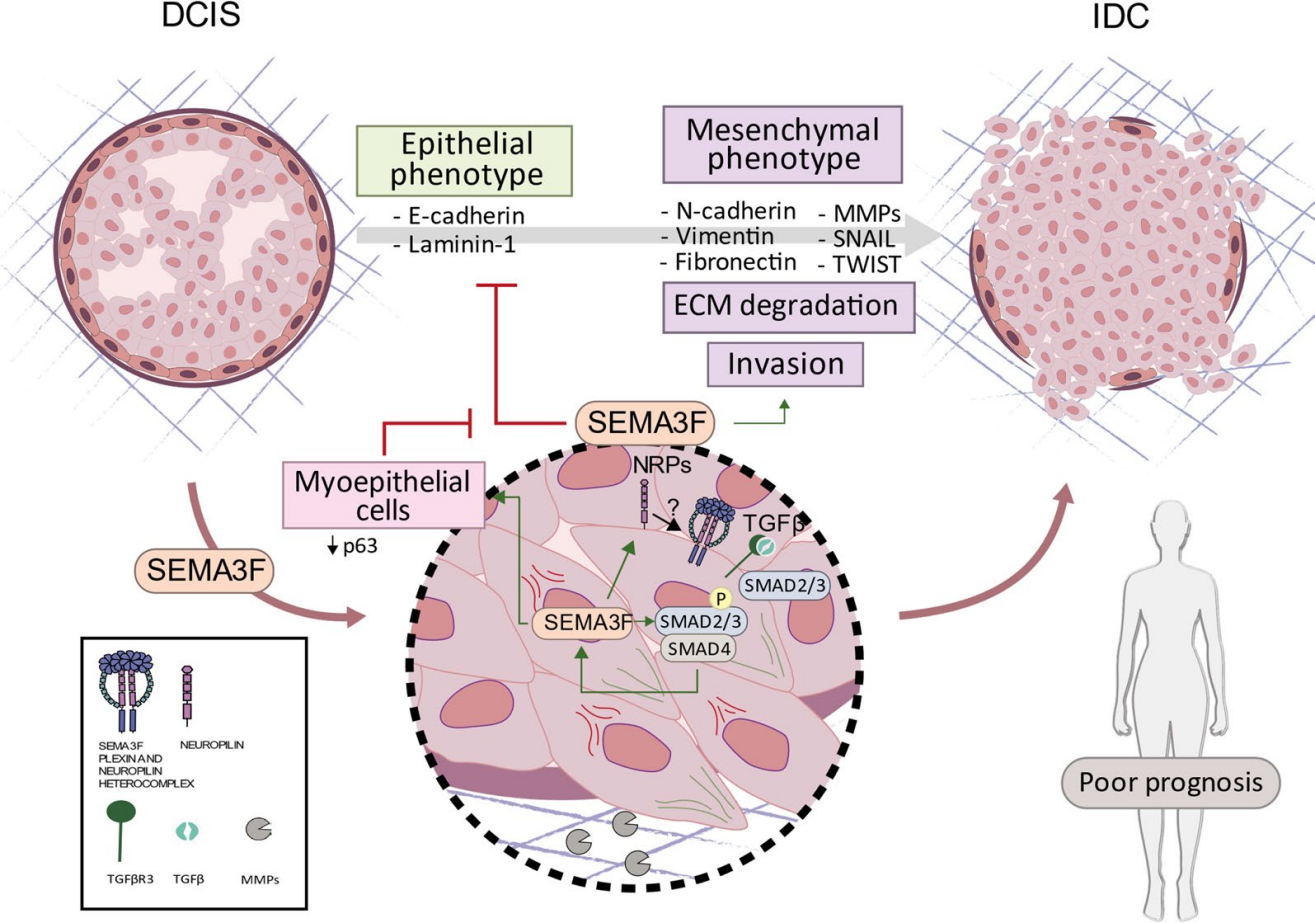
SEMA3F induces EMT, ECM degradation and invasion, promoting DCIS-to-IDC transition. SEMA3F is a protumorigenic molecule that promotes EMT and increases the invasive capacity of epithelial cells, affects the integrity of acinar structure, as well as the ability to degrade the extracellular matrix by decreasing E-cadherin and laminin-1 and increasing N-cadherin, vimentin, fibronectin, MMPs, SNAIL and TWIST levels. It is likely that these SEMA3F-induced tumorigenic functions are mediated through coreceptors, NRPs, and the TGFβ signalling pathway, among others. Moreover, SEMA3F affects not only epithelial cells but also myoepithelial cells, affecting their antitumorigenic activity and promoting a more malignant phenotype (indicated by p63 downregulation)

Taken together, these results further corroborate our in vitro and in vivo data and reveal the importance of the SEMA3F/NRP1/NRP2 complex in the progression and acquisition of invasive features in DCIS lesions from bench to bedside (Fig. 8). Likewise, these data indicate that SEMA3F/NRP1/NRP2 could be considered new therapeutic targets and relevant poor prognostic biomarkers for patient clinical management and outcomes in DCIS lesions (Fig. 8). These findings could contribute to improving the accuracy of the initial diagnosis and prognosis of DCIS BC patients with substantial clinical implications.

## Discussion

Here, we report a novel role for SEMA3F through its coreceptors NRP1 and NRP2. SEMA3F enables the transition from an epithelial breast cancer phenotype to a mesenchymal phenotype, promoting invasive traits and initiating noninvasive to invasive transition. NRP1 and NRP2 have already been implicated in cancer invasion, metastasis and progression [16, 64–68], but this is not the case for SEMA3F, which has been traditionally considered a good prognostic marker not only for BC but also for several other types of cancer [69]. Our results demonstrate for the first time that SEMA3F can be an adequate marker of poor prognosis during the DCIS transition to invasion in BC patients. This finding is in agreement with previous reports in hepatocarcinoma [30, 31] and in other types of cancer, including BC [32], and reveals SEMA3F and its coreceptors as candidates for poor prognostic markers in cancer and possible cancer therapeutic targets.

SEMA3F overexpression clearly induced a more invasive phenotype, which was particularly obvious in 3D cell cultures and in our in vivo assay model. The morphological changes and polarity alterations shown by mild-aggressive cell lines under the action of SEMA3F overexpression indicate that this represents a strong challenge for these cells to form their typical organized rounded-shaped and polarized 3D growths [36]. Moreover, our novel observation of the negative effects of SEMA3F on laminin-1, a key component of the basal membrane, together with the induction of high collagenase activity, links SEMA3F to the disappearance of the basal membrane, a physical and biological obstacle for DCIS progression to IDC [70]. The effects of SEMA3F are likely due to its autocrine/paracrine effects on both epithelial and myoepithelial cells, with the latter playing a key role as a tumor suppressor in BC [71, 72]. Our results suggest that these effects could be mediated through the NRP1/NRP2 coreceptors since their localization via 3D immunofluorescence seems to be compatible with the myoepithelial cell compartment at the acinus edge. In agreement with our results, NRP1 expression was found to be greater in myoepithelial cells from tumor tissues than in those from healthy tissues, and NRP1 expression was reduced during the progression to IDC [73], which could lead to complete elimination of the myoepithelial cell layer that surrounds the duct. In fact, this increase in myoepithelial NRP1 expression could be a prerequisite for the disappearance of myoepithelial cells [73]. Obviously, additional efforts must be devoted to clarifying the role of SEMA3F as an invasiveness cue in myoepithelial cells, but our results, as well as those of others [73, 74], reinforce the view that SEMA3F is produced by epithelial cancer cells prior to its progression to IDC and that myoepithelial cells are affected by local SEMA3F production at this precise temporal step in BC.

The role of SEMA3F in the acquisition of an aggressive phenotype in BC cells is not limited to direct EMT activation. SEMA3F induces an increase in the expression of β-catenin, a member of the Wnt signalling pathway involved in the acquisition of invasive capacity in cancer cells and related to poor survival [75]. Interactions between the Wnt/β-catenin pathway and the TGFβ pathway are common and have been implicated both in fostering metastasis through the EMT process in BC [76] and in ECM remodelling through the stimulation of MMP synthesis and secretion [77]. SEMA3F seems to be involved in all these processes. In fact, MMP2 and MMP14 in the conditioned media of mild aggressive cancer cells were strongly activated by SEMA3F, in accordance with previous results that demonstrated that these MMPs are involved in the cleavage of collagen IV [78], a basal membrane compound. In our 3D cell cultures, enhanced collagenase activity and invasive capacity were observed after SEMA3F treatment. In addition, TIMP2, whose secretion is enhanced by SEMA3F, has been previously described as a key protein involved in MMP2 and MMP14 activation, is specifically linked to the induction of migration in BC cells [79] and is positively correlated with poor prognosis in BC patients [80]. We also report that as a part of the EMT program, SEMA3F overexpression upregulates the expression of the TGFβ pathway, the most important cell signalling pathway regulating EMT processes and strongly implicated in BC progression [81, 82]. In fact, the induction of a mesenchymal phenotype by SEMA3F occurs through the activation of the canonical pathway by TGFβ, which affects TGFβR3 gene expression and downstream effectors such as SMAD2 and SMAD4. When SMAD2 was knocked down, SEMA3F overexpression in mild-aggressive BC cell lines was completely abrogated, even in the SEMA3F-overexpressing cell line. In turn, TGFβ also positively modulates the levels of SEMA3F. This relationship between SEMA3F and TGFβ in a model of early preinvasive BC parallels the increase in plasma TGFβ levels found in patients at this initial step [83]. The most obvious explanation is that SEMA3F stimulates the TGFβ pathway, but another possibility can coexist with this first pathway in an in vivo tumor context, i.e., that the induction of MMP2, and MMP14 by SEMA3F, which have been implicated in the proteolytic cleavage-mediated activation of the TGFβ signal [58, 84], might occur, thus triggering the EMT program. NRP1 could also be implicated in the interaction between SEMA3F and TGFβ since it is known to play a role in stimulating the TGFβ pathway, activating the EMT and eventually leading to the acquisition of an invasive phenotype [66, 85]. NRP1 and NRP2 are also coreceptors of TGFβ1 involved in the activation of their latent molecules [66, 85]; therefore, this could represent another step in the mechanism linking SEMA3F and TGFβ. Even though additional experiments must be carried out to better clarify the relationship between TGFβ and SEMA3F signalling in DCIS transition to IDC, our results clearly demonstrate that there is positive feedback between these two proteins that supports invasion.

Although SEMA3F has a tenfold greater affinity for NRP2 than for NRP1 [86], both coreceptors are clearly implicated in SEMA3F signalling in BC [85, 87]. In accordance with these data, blockade of NRP1 and NRP2 in MCF10DCIS-SEMA3F cells resulted in complete inhibition of the 3D cell culture effects produced by SEMA3F overexpression. Although we cannot exclude the contribution of the inhibition of the TGFβ pathway to this effect, our findings also point toward a new mechanism of SEMA3F proinvasive action in the DCIS-to-IDC transition in BC through both NRP coreceptors, highlighting its demonstrated role in BC progression [85]. Our results also reveal a connection between these two coreceptors that can contribute to the SEMA3F signal together or in parallel. Most importantly, since NRP2 does not seem to play a key role in normal adult tissues, blocking the effects of SEMA3F on DCIS progression might be a good new therapeutic approach for preventing secondary effects on healthy regions [88]. Similarly, two soluble NRP1 isoforms have been described as inhibitory tools for MDA-MB-231 BC cell migration mediated by nonsoluble NRP1 receptors [89].

To evaluate the potential role of SEMA3F-NRP1/NRP2 as therapeutic targets and prognostic markers in the DCIS stage of BC and to start moving our results from bench to bedside, gene expression data from tumors from all BC stages in the GOBO database were analysed. The results obtained corroborate our in vitro and in vivo findings, as well as other previous data in the literature [64, 90], suggesting that correlating SEMA3F and its coreceptors NRP1 and NRP2 with poor prognosis in BC patients. Results analyzed in DCIS I (more invasive) and DCIS II (less invasive) suggest that SEMA3F is induced early in DCIS, but consistent with our NRP1/NRP2 blockade analysis, only when SEMA3F and NRP1/NRP2 expression show a positive correlation, that is in DCIS I and invasive lesions, SEMA3F is fully able to signal as a proinvasive cue. Furthermore, the specific analysis of database samples comparing pure DCIS patients to IDC patients strongly confirmed that SEMA3F, NRP1 and NRP2 were upregulated in the invasive stage and that their expression was correlated with TGFβ pathway stimulation and with increased activation of MMPs, a trend highly related to EMT. These data show the clinical importance of SEMA3F, NRP1 and NRP2 in DCIS-to-IDC BC progression and correlate with the activation of EMT in IDC versus DCIS [37]. In agreement with our data, previous results from our laboratory revealed that NRP2 is related to poor prognosis [16] in patients with the basal-like BC subtype, similar to the findings for NRP1 [91] and SEMA3F [32]. Considering that SEMA3F positively correlates with an estrogen response and NRP1 positively correlates with the basal-like BC subtype, this could be explained by the fact that NRP1 acts as a co-receptor for many ligands, including those that control survival, migration and invasion, which are particularly important in basal breast cancer, such as VEGF, which leads to tumorigenesis and metastasis [92]. In addition, the association between SEMA3F and an enrichment of estrogen response genes is partially consistent with the results of an interesting study by Strand and colleagues, which suggests that in some cases ERhigh is associated with DCIS recurrence [93]. The strong correlation between NRP1 or NRP2 and the stromal score could indicate that these coreceptors play a key role in stromal cells, as it has been already proposed [74]. However, the role of these cells in myoepithelial and other microenvironmental cells might be worth exploring.

Moreover, the combination of our proposed biomarkers SEMA3F, NRP1 and NRP2 of DCIS progression risk with other previously identified [93, 94] and other prognostic parameters such as margin status and DCIS dimensions [95] may help to better stratify these patients into low and high-risk groups and overcome the problem of overtreatment. Additionally, further research efforts are needed to demonstrate that SEMA3F, NRP1 and NRP2 could work as risk progression markers not only for DCIS, but also for the other pre-invasive lesion in BC, lobular carcinoma in situ (LCIS), and especially for those that are submitted to treatment, the pleomorphic LCIS [96].

## Conclusions

In summary (Fig. 8), we demonstrated for the first time that SEMA3F, via its coreceptors NRP1 and NRP2, is a strong straightforward invasive cue that activates the EMT program and facilitates the DCIS-to-IDC transition. In this sense, SEMA3F stimulates the TGFβ pathway, which in turn increases SEMA3F expression, establishing a proinvasive loop. Although NRP1 and NRP2 also affect myoepithelial cell tumor suppressor function, cells presence and integrity are disturbed, clearly disrupting the basal membrane structure. Patient data regarding SEMA3F and its coreceptors NRP1 and NRP2 validate them as DCIS evolution prognosis markers for IDC and as new therapeutic targets, as has been previously described, to keep preinvasive BC lesions in this more therapeutically affordable BC stage.

### Supplementary Information

Supplementary Material 1.

Supplementary Material 2.

Supplementary Material 3.

Supplementary Material 4.

Supplementary Material 5.

Supplementary Material 6.

Supplementary Material 7.

## Data Availability

The datasets used in this manuscript are public and available (for more detailed information, see the methods section, Bioinformatics procedure/Analysis of database part), and the analyses performed during the current study are available from the corresponding author upon reasonable request.

## Abbreviations

SEMA3F: Semaphorin 3F
NRP: Neuropilin
DCIS: Ductal carcinoma in situ
IDC: Invasive ductal carcinoma
BC: Breast cancer
EMT: Epithelial to mesenchymal transition
ECM: Extracellular matrix
FBS: Foetal bovine serum
HS: Horse serum
EGF: Epithelial growth factor
ATCC: American type culture collection
CM: Conditioned medium
CCM: Concentrated conditioned medium
WB: Western blot
TET-on: Tetracycline-controlled expression
CAM: Chicken chorioallantoic membrane
GOBO: Gene expression-based outcome for breast cancer
GEO: Gene expression omnibus
GSEA: Gene set enrichment analysis
LCIS: Lobular carcinoma in situ
