# Supplementary material for: The SEMA3F-NRP1/NRP2 axis is a key factor in the acquisition of invasive traits in in situ breast ductal carcinoma
Source: Breast Cancer Res. 2024 Aug 13;26:122. doi: 10.1186/s13058-024-01871-0 (PMC11320849; doi:10.1186/s13058-024-01871-0)
Supplement: Supplementary file 1 — Supplementary Material 1. [file 13058_2024_1871_MOESM1_ESM.pdf]

Supplementary Figure 1

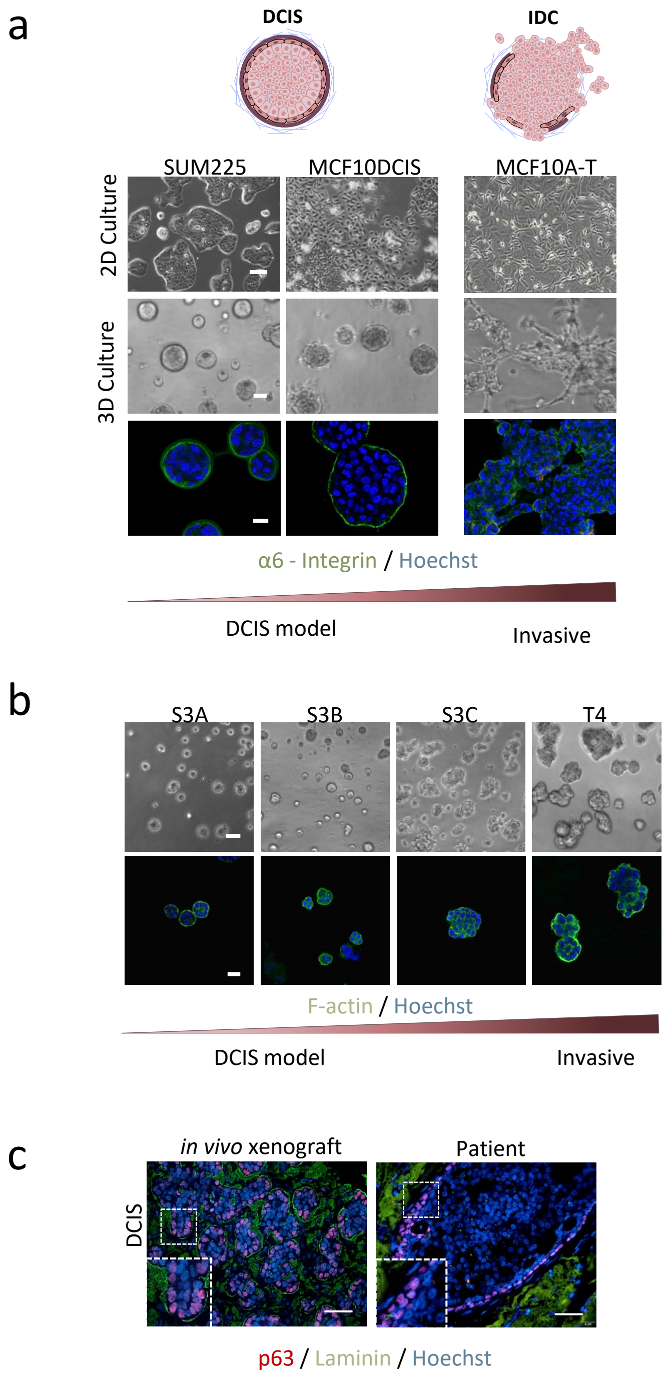

Suppl. Figure 1. *In vitro* and *in vivo* systems of DCIS and potential linear progression of BC. a) Graphical and photographic representation of the used BC *in vitro* transition model, comprising DCIS (SUM225 and MCF10DCIS) and invasive or IDC (MCF10A-T) cell lines. Top and middle panels, representative 2D (top; scale bar: 75µm) and 3D (middle; scale bar: 50µm) phase contrast images. Bottom panels, representative IF images of α6-integrin (scale bar: 20µm). **b)** 3D cultures of the HMT-3522 S1-derived cell lines, arranged from less to more aggressive cell lines: S3A, S3B, S3C and T4. Top panels illustrate representative 3D phase contrast images (scale bar: 50µm). Bottom panels showcase representative IF images of F-actin (phalloidin) (scale bar: 50µm). **c)** Representative IF images of p63 (red) and laminin (green) in CAM *in vivo* xenograft model (left panels) and patients' samples (right panels) with DCIS breast cancer. Scale bar: 40µm.
