# Supplementary material for: The SEMA3F-NRP1/NRP2 axis is a key factor in the acquisition of invasive traits in in situ breast ductal carcinoma
Source: Breast Cancer Res. 2024 Aug 13;26:122. doi: 10.1186/s13058-024-01871-0 (PMC11320849; doi:10.1186/s13058-024-01871-0)
Supplement: Supplementary file 2 — Supplementary Material 2. [file 13058_2024_1871_MOESM2_ESM.pdf]

Supplementary Figure 2

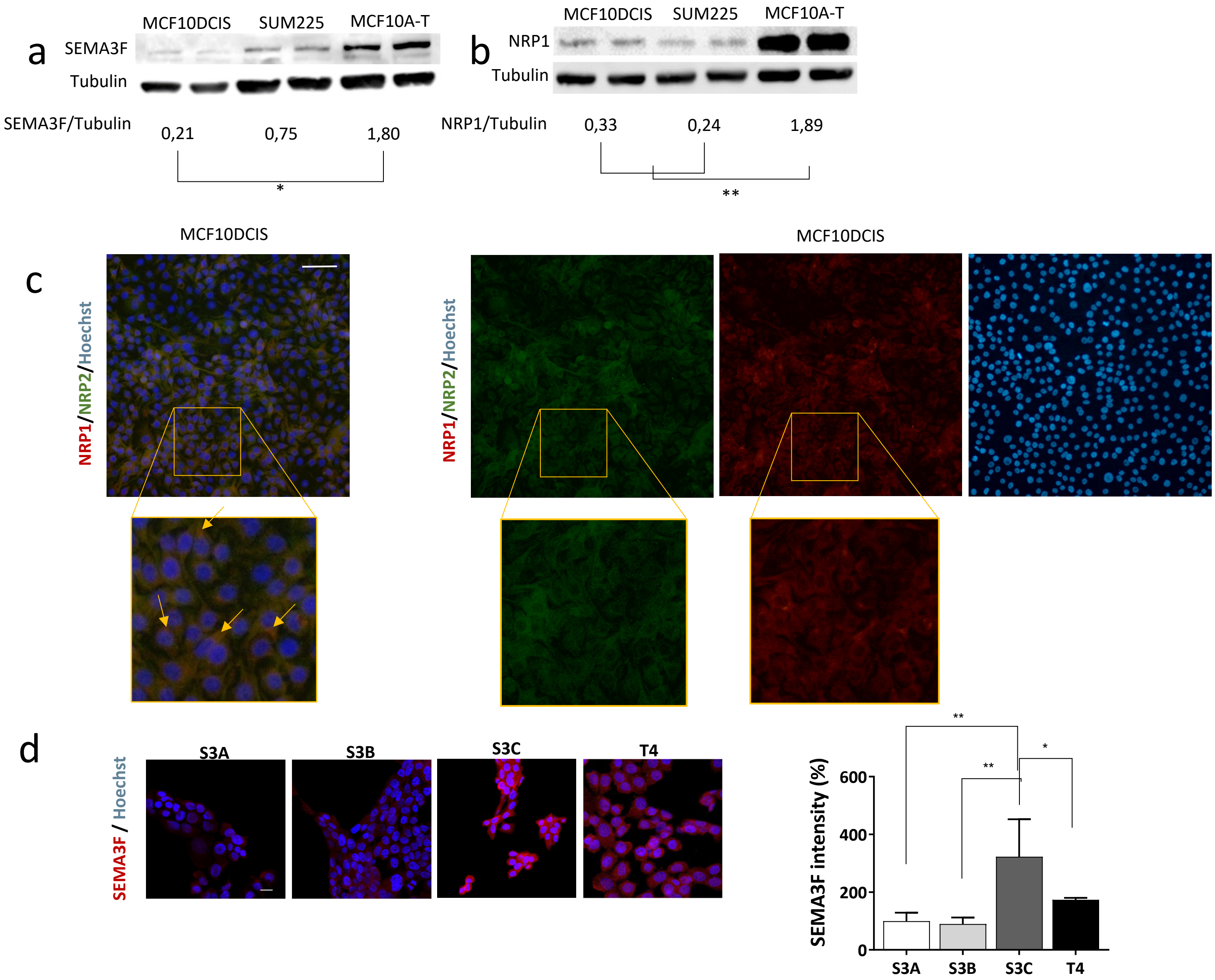

Suppl. Figure 2. Characterization of DCIS to IDC transition model. a, b) Representative western blot analysis of SEMA3F (**A**) and NRP1 (**B**) protein levels normalized with tubulin in the *in vitro* DCIS to IDC transition model. *P < 0.05, **P < 0.01 comparing protein quantification of all tumor cell lines by one-way ANOVA, Mann-Whitney's test. **c)** Representative double IF images for MCF10DCIS.com cells of NRP1 (red) and NRP2 (green) and nuclei (Hoechst blue), and amplification details. Merged channels left panel, and individual channels in right panel. Yellow arrows indicate colocalization of both receptors (scale bar: 50 µm). **d)** Representative IF images (left panels; scale bar: 20µm) and mfi quantification (right panel) for SEMA3F in the HMT-3522 S1-derived cell lines, ordered from less to more aggressive cell lines: S3A, S3B, S3C and T4. The graph represents the percentage (%) of mfi normalized to S3A ± S.E.M.; *P < 0.05, **P < 0.01 comparing all tumor cell lines by one-way ANOVA, Mann-Whitney's test
