# Supplementary material for: The SEMA3F-NRP1/NRP2 axis is a key factor in the acquisition of invasive traits in in situ breast ductal carcinoma
Source: Breast Cancer Res. 2024 Aug 13;26:122. doi: 10.1186/s13058-024-01871-0 (PMC11320849; doi:10.1186/s13058-024-01871-0)
Supplement: Supplementary file 3 — Supplementary Material 3. [file 13058_2024_1871_MOESM3_ESM.pdf]

Supplementary Figure 3

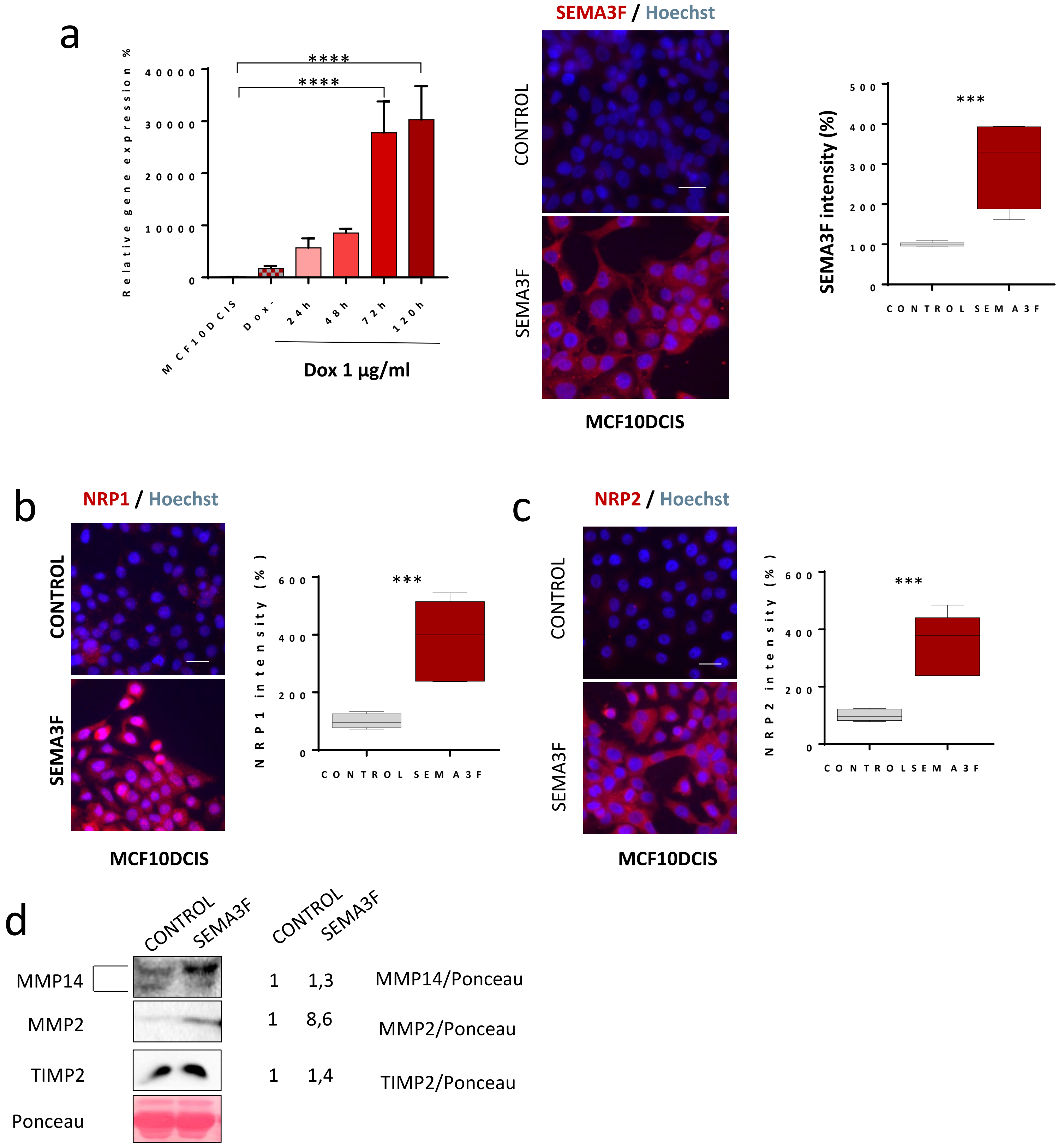

Suppl. Figure 3. Effects of SEMA3F over-expression in MCF10DCIS cell line. A) Left panel, analysis of *SEMA3F* mRNA relative expression in MCF10DCIS cells. SEMA3F expression was induced by 24, 48, 72 or 120h of doxycycline (Dox; 1µg/mL) treatment. The graph represents the % of mRNA levels ± S.E.M. referred to non-treated MCF10DCIS cells (control); ****P < 0.0001 comparing non-treated *vs* SEMA3F overexpressing MCF10DCIS cells by one-way ANOVA, Mann-Whitney's test. Middle and right panels, representative IF images (middle panel; scale bar: 20µm) and mfi quantification (right panel) for SEMA3F in control or SEMA3F overexpressing MCF10DCIS cells. The graph represents the % of mfi levels ± S.E.M. referred to control cells; ***P < 0.001 comparing control *vs* SEMA3F overexpressing MCF10DCIS cells by one-way ANOVA, Mann-Whitney's test. **B, C)** Representative IF images (left panel; scale bar: 20µm) and mfi quantification (right panel) for NRP1 (**B**) and NRP2 (**C**) in control or SEMA3F overexpressing MCF10DCIS cells. The graphs represent the % of mfi levels ± S.E.M. referred to control cells; ***P < 0.001 comparing control *vs* SEMA3F overexpressing MCF10DCIS cells by one-way ANOVA, Mann-Whitney's test. **D)** Representative Western blot analysis (left panel) of protein levels of MMP14, MMP2 and TIMP2 and a representative image of Ponceau staining. Quantification normalised to total Ponceau-stained lane (right panel) in SEMA3F overexpressing MCF10DCIS cells.
