# Supplementary material for: The SEMA3F-NRP1/NRP2 axis is a key factor in the acquisition of invasive traits in in situ breast ductal carcinoma
Source: Breast Cancer Res. 2024 Aug 13;26:122. doi: 10.1186/s13058-024-01871-0 (PMC11320849; doi:10.1186/s13058-024-01871-0)
Supplement: Supplementary file 4 — Supplementary Material 4. [file 13058_2024_1871_MOESM4_ESM.pdf]

Supplementary Figure 4

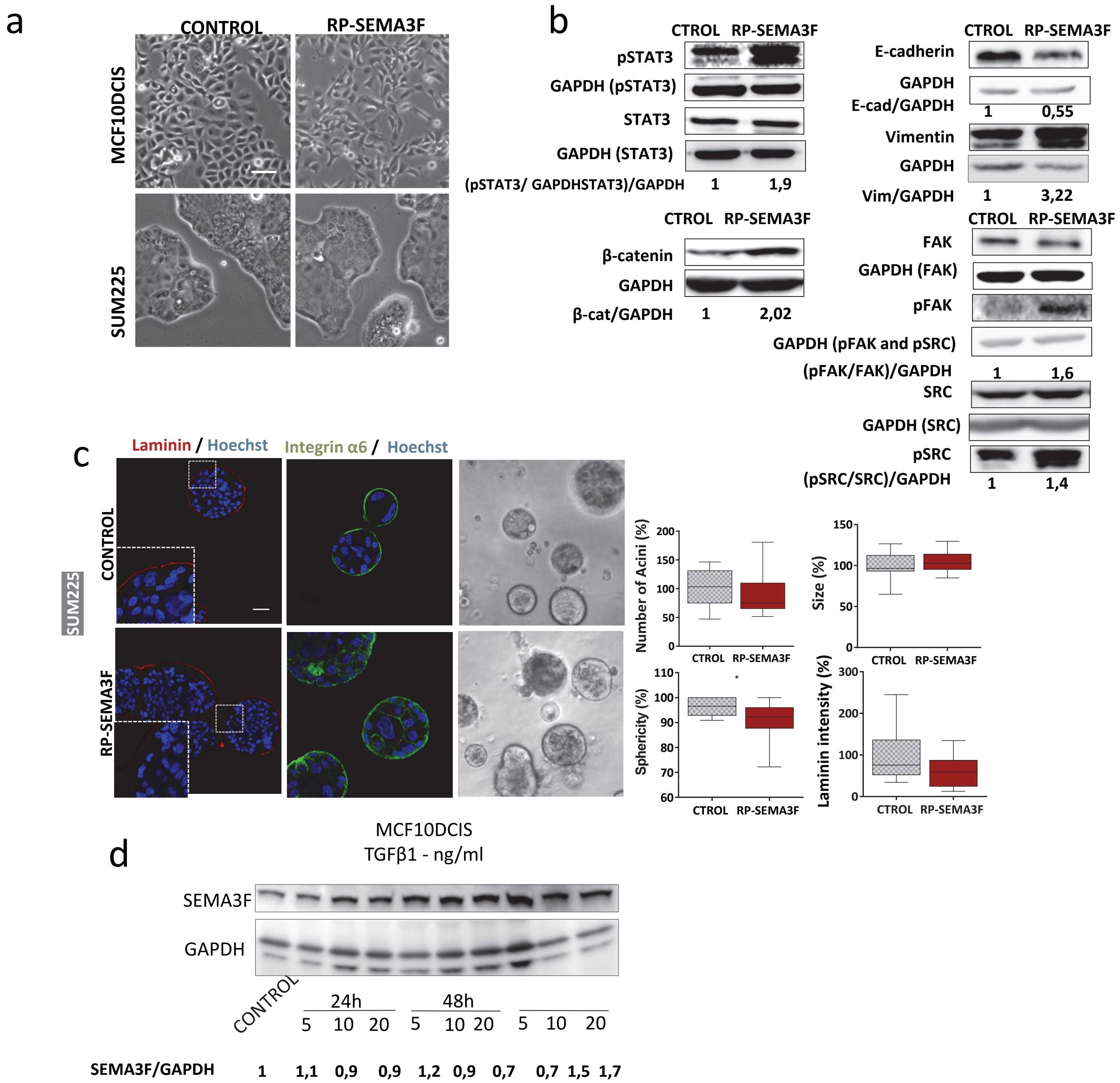

Suppl. Figure 4. Bidirectional relationship between EMT program activation and SEMA3F in MCF10DCIS cells. A) Representative bright field images of MCF10DCIS and SUM225 cells after chronic SEMA3F treatment (RP-SEMA3F) (100ng/mL for 2 weeks). Scale bar: 50µm. **B)** Representative western blot analysis of pSTAT3/STAT3, E-cadherin, Vimentin, β-catenin, pFAK/FAK and pSRC/SRC protein levels normalized with GAPDH in MCF10DCIS cells chronically treated with SEMA3F (RP-SEMA3F. Protein quantifications are referred to the non-treated control condition. **C)** Representative 3D phase contrast (right images; scale bar: 50µm) and IF images (left and middle images; scale bar=20µm for Laminin, scale bar=50µm for Integrin) and the % of the number of acini, size, sphericity and laminin mfi quantification (right panels) in SUM225 cells chronically treated with SEMA3F (RP-SEMA3F) (10ng/mL for 2 weeks). **D)** Representative western blot analysis of SEMA3F protein levels normalized with GAPDH in TGFβ1-treated MCF10DCIS cells. Cells were treated with increasing doses (5-10-20ng/mL) for 24, 48 and 72h. Protein quantifications are referred to the non-treated control condition. The graphs represent mean values ± S.E.M.; non-significant differences were observed comparing control *vs* SEMA3F-treated cells by one-way ANOVA, Mann-Whitney's test.
