# Supplementary material for: The SEMA3F-NRP1/NRP2 axis is a key factor in the acquisition of invasive traits in in situ breast ductal carcinoma
Source: Breast Cancer Res. 2024 Aug 13;26:122. doi: 10.1186/s13058-024-01871-0 (PMC11320849; doi:10.1186/s13058-024-01871-0)
Supplement: Supplementary file 5 — Supplementary Material 5. [file 13058_2024_1871_MOESM5_ESM.pdf]

Supplementary Figure 5

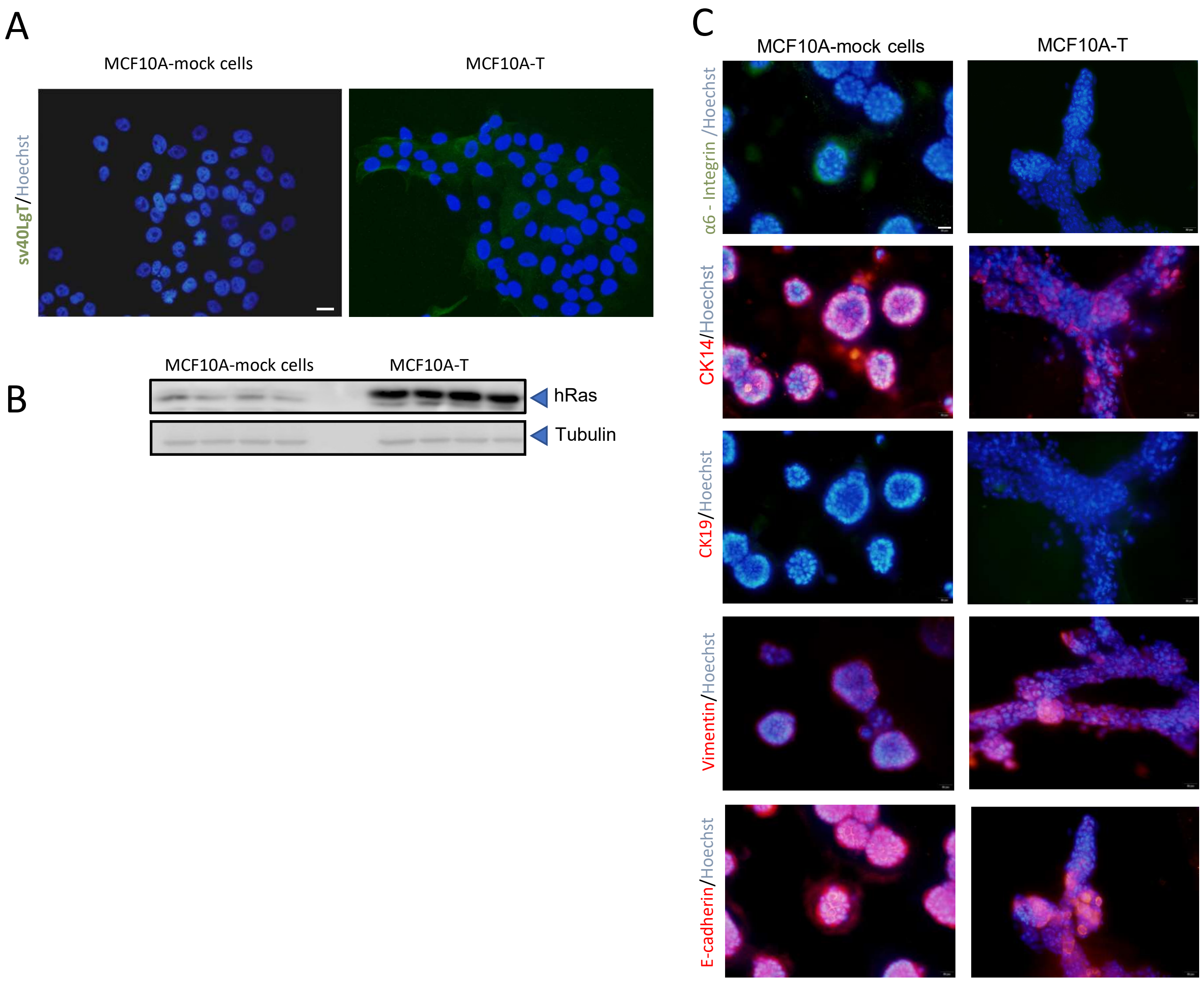

Suppl. Figure 5. MCF10A-Transformed (MCF10A-T) generation from MCF10A and characterization. A) Representative IF images of the oncoprotein SV40LgT in wild-type (mock) or transformed MCF10A (MCF10A-T) cells. Scale bar: 200µm . **B)** Representative western blot analysis of hRas protein levels normalized with β-Tubulin in wild-type (mock) or transformed MCF10A (MCF10A-T) cells. Quadrupled samples. **C)** Representative IF images of CD49f, CK14, CK19, Vimentin and E-cadherin in wild-type (mock) or transformed MCF10A (MCF10A-T) cells. Scale bar : 50µm.
