# Supplementary material for: The SEMA3F-NRP1/NRP2 axis is a key factor in the acquisition of invasive traits in in situ breast ductal carcinoma
Source: Breast Cancer Res. 2024 Aug 13;26:122. doi: 10.1186/s13058-024-01871-0 (PMC11320849; doi:10.1186/s13058-024-01871-0)
Supplement: Supplementary file 6 — Supplementary Material 6. [file 13058_2024_1871_MOESM6_ESM.pptx]

## Slide 1
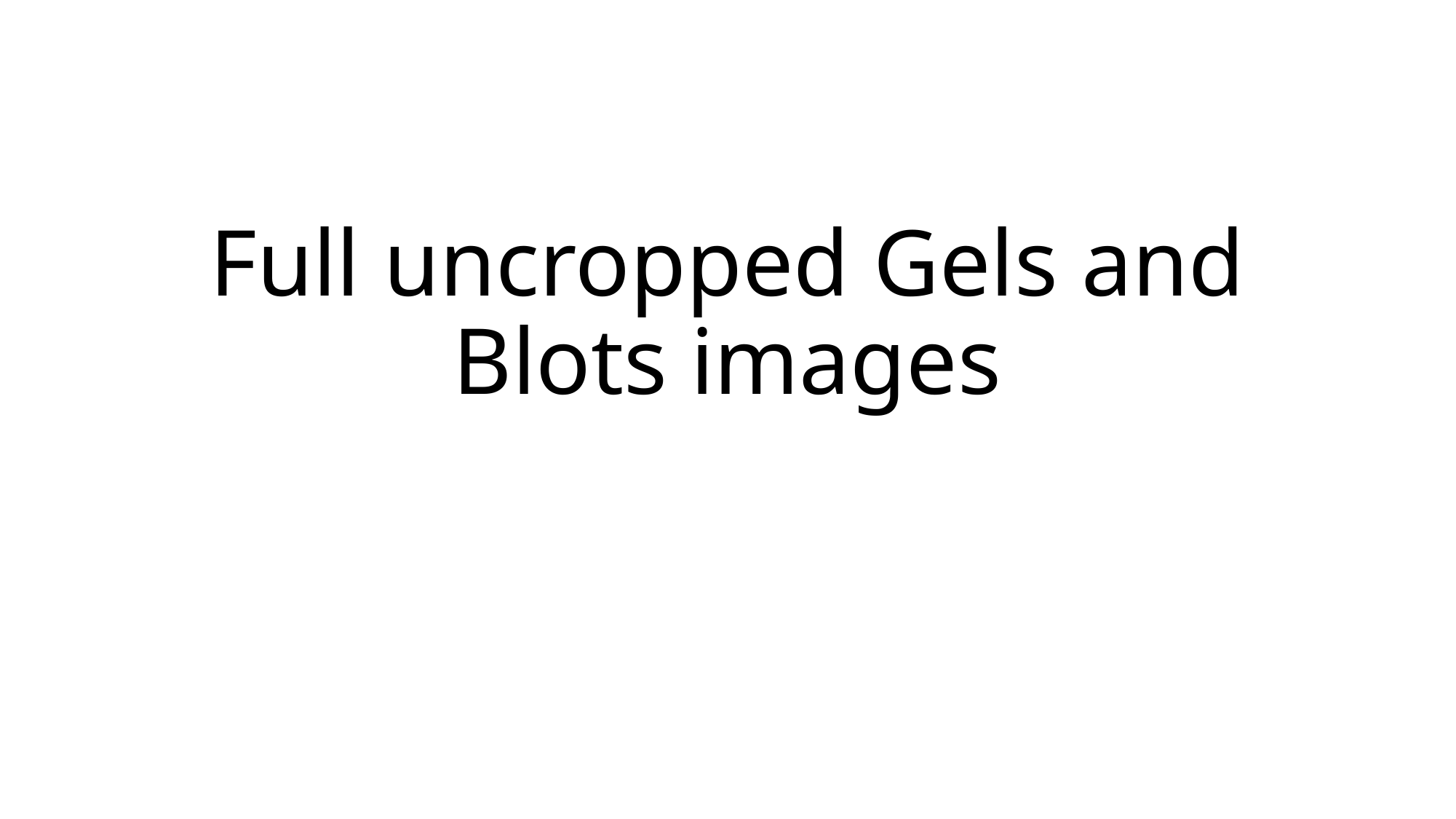

# Full uncropped Gels and Blots images

## Slide 2
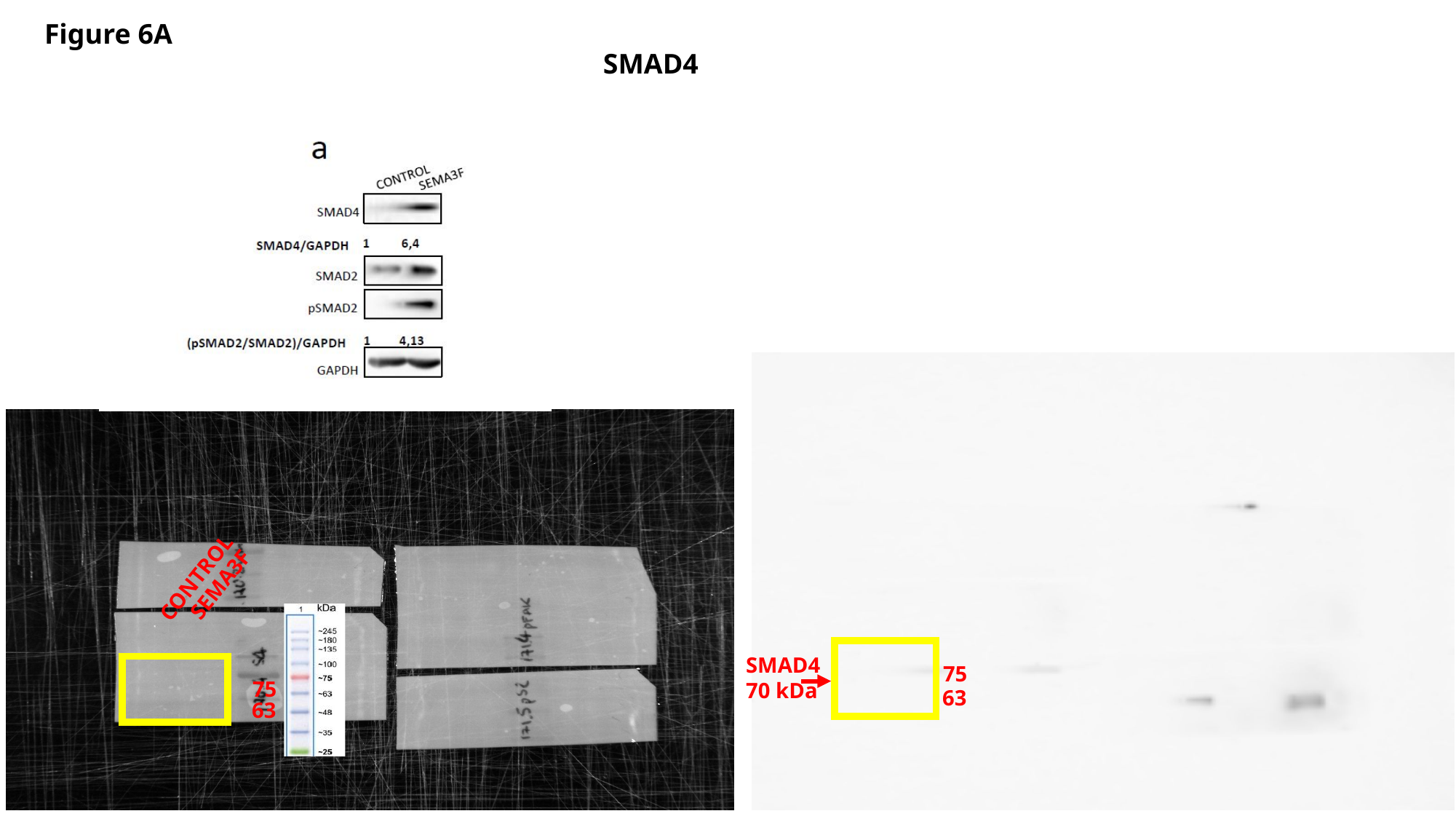

Figure 6A
SMAD4
SMAD4
70 kDa
75
63
CONTROL
SEMA3F
75
63

## Slide 3
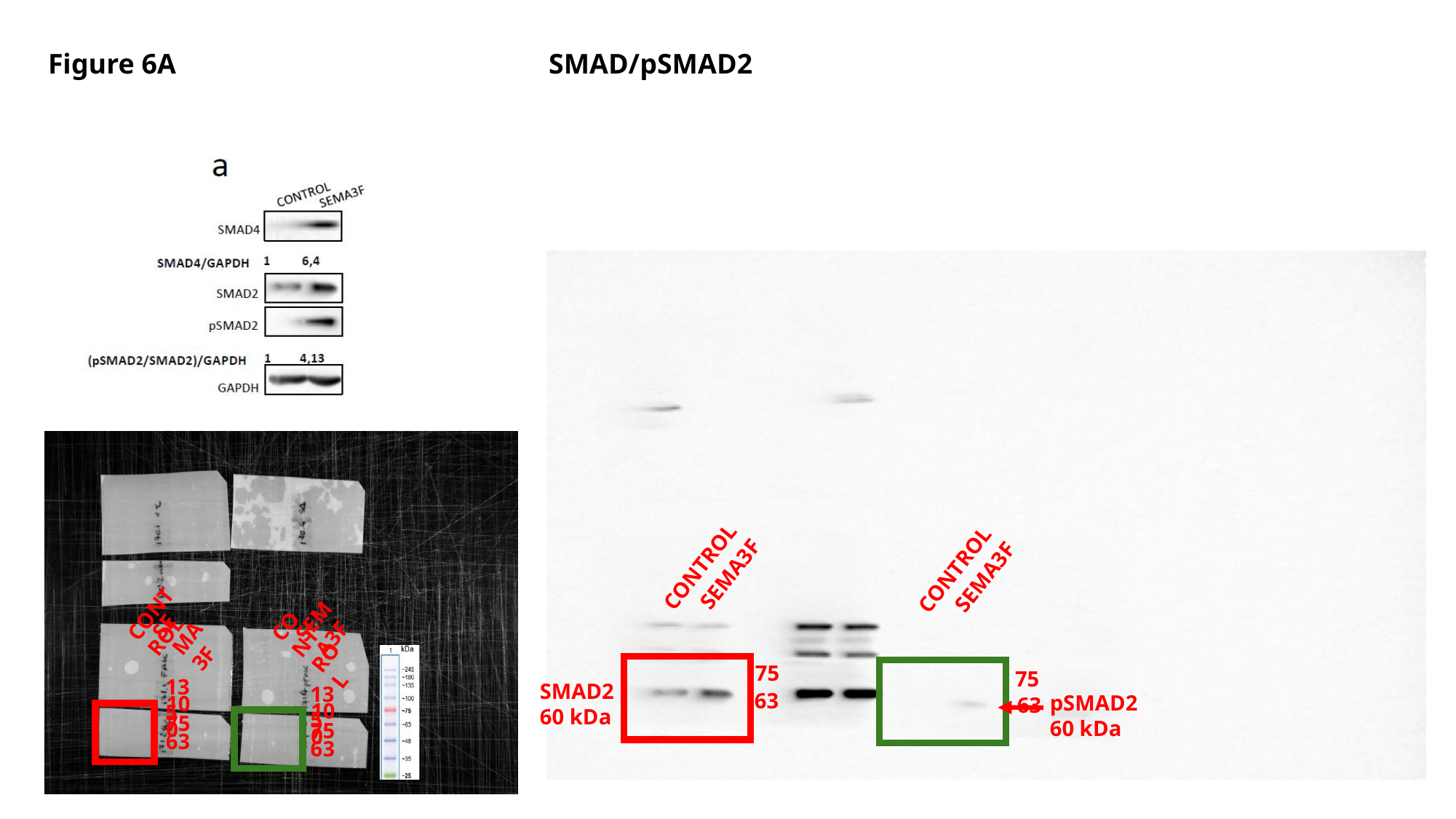

Figure 6A
SMAD/pSMAD2
CONTROL
CONTROL
SEMA3F
SEMA3F
75
75
SMAD2
60 kDa
63
pSMAD2
60 kDa
CONTROL
SEMA3F
SEMA3F
CONTROL
135
135
100
100
75
75
63
63
63

## Slide 4
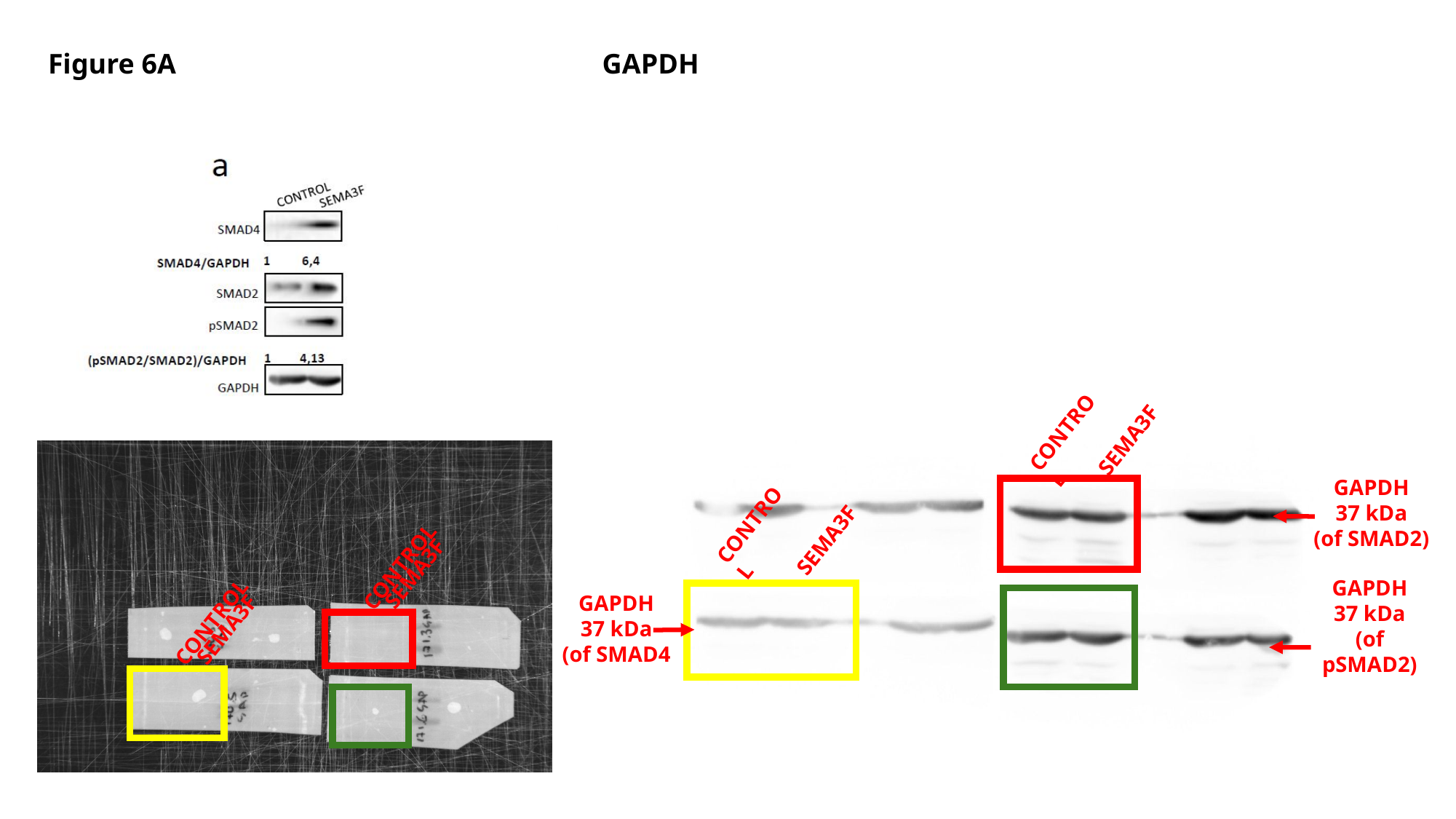

Figure 6A
GAPDH
CONTROL
SEMA3F
GAPDH
37 kDa
(of SMAD2)
CONTROL
SEMA3F
GAPDH
37 kDa
(of pSMAD2)
GAPDH
37 kDa
(of SMAD4
CONTROL
SEMA3F
CONTROL
SEMA3F

## Slide 5
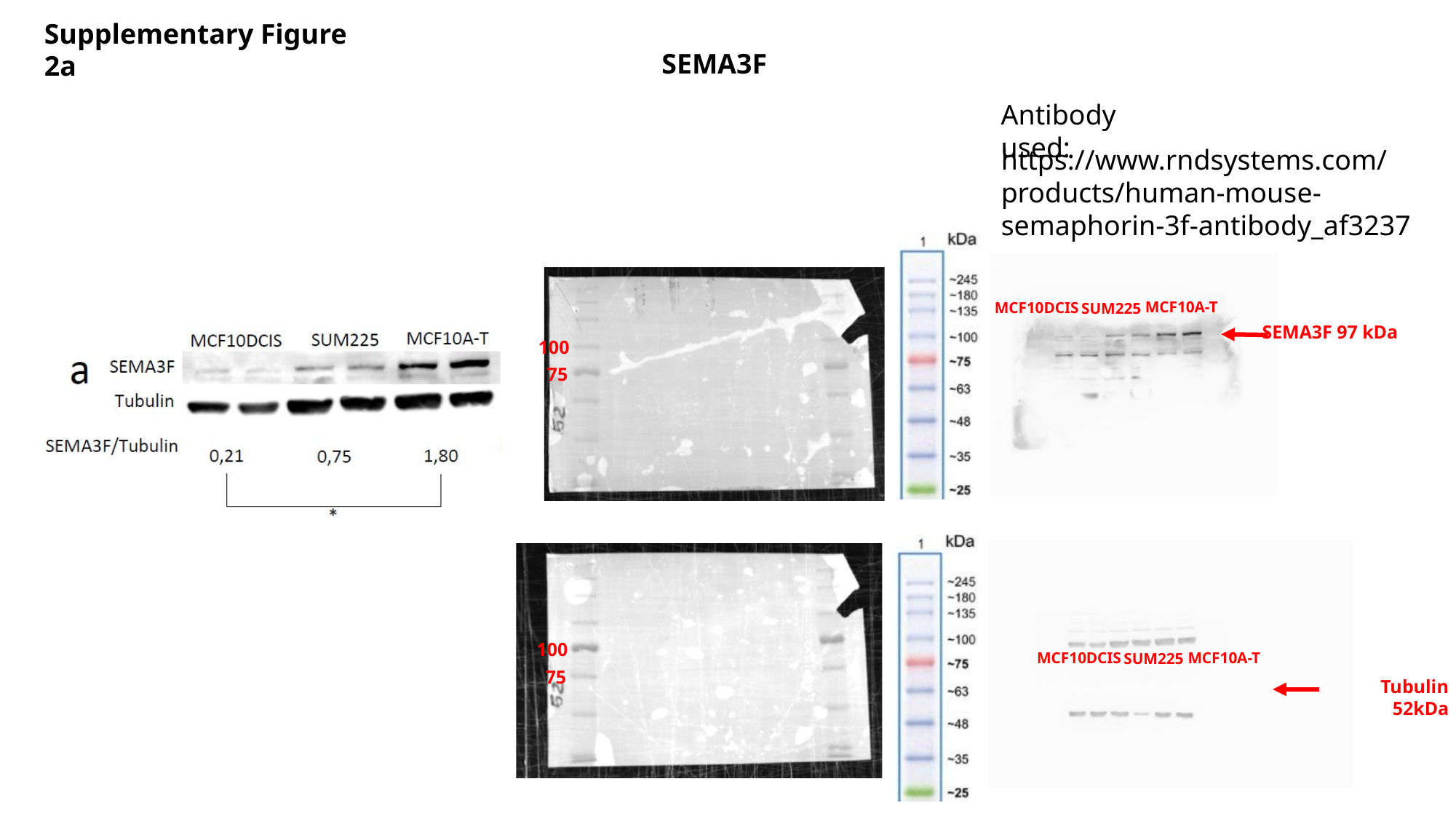

Supplementary Figure 2a
SEMA3F
Antibody used:
https://www.rndsystems.com/products/human-mouse-semaphorin-3f-antibody_af3237
MCF10A-T
MCF10DCIS
SUM225
SEMA3F 97 kDa
100
75
100
MCF10A-T
MCF10DCIS
SUM225
75
Tubulin 52kDa

## Slide 6
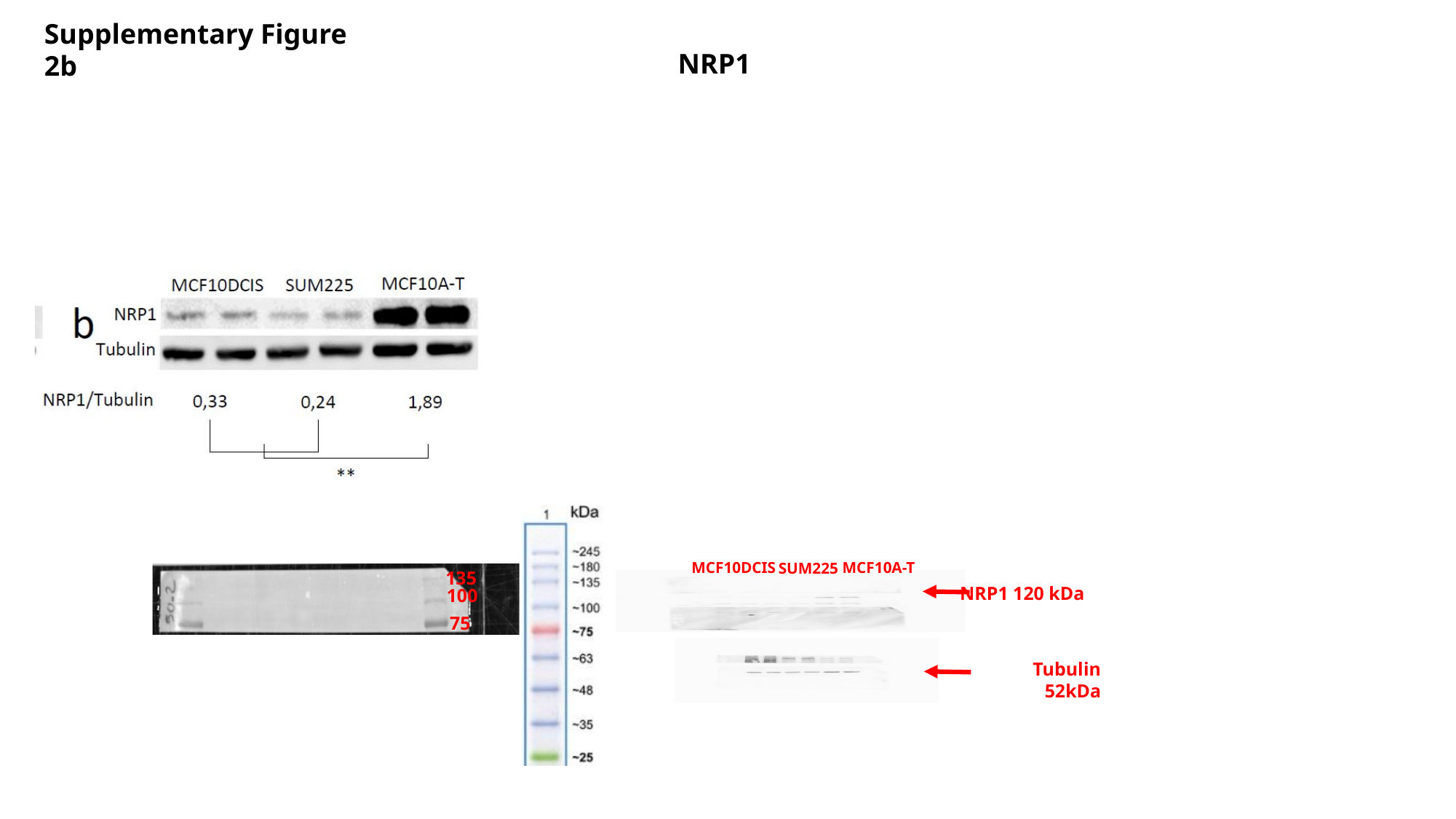

Supplementary Figure 2b
NRP1
MCF10A-T
MCF10DCIS
SUM225
135
NRP1 120 kDa
100
75
Tubulin 52kDa

## Slide 7
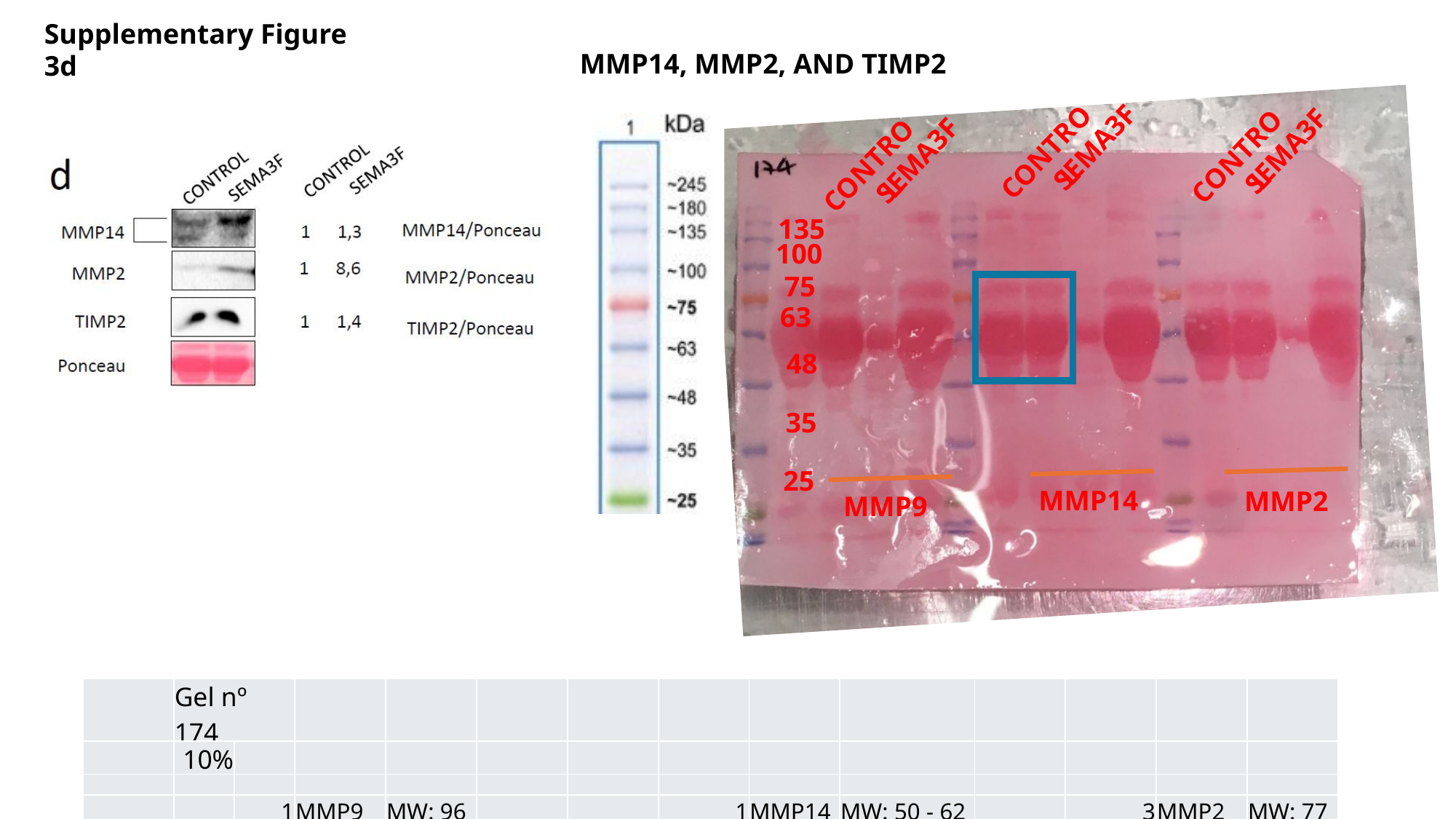

Supplementary Figure 3d
MMP14, MMP2, AND TIMP2
SEMA3F
SEMA3F
CONTROL
CONTROL
SEMA3F
CONTROL
135
100
75
63
48
35
25
MMP14
MMP2
MMP9
| | Gel nº 174 | | | | | | | | | | | | |
| --- | --- | --- | --- | --- | --- | --- | --- | --- | --- | --- | --- | --- | --- |
| | 10% | | | | | | | | | | | | |
| | | | | | | | | | | | | | |
| | | 1 | MMP9 | MW: 96 | | | 1 | MMP14 | MW: 50 - 62 | | 3 | MMP2 | MW: 77 |

## Slide 8
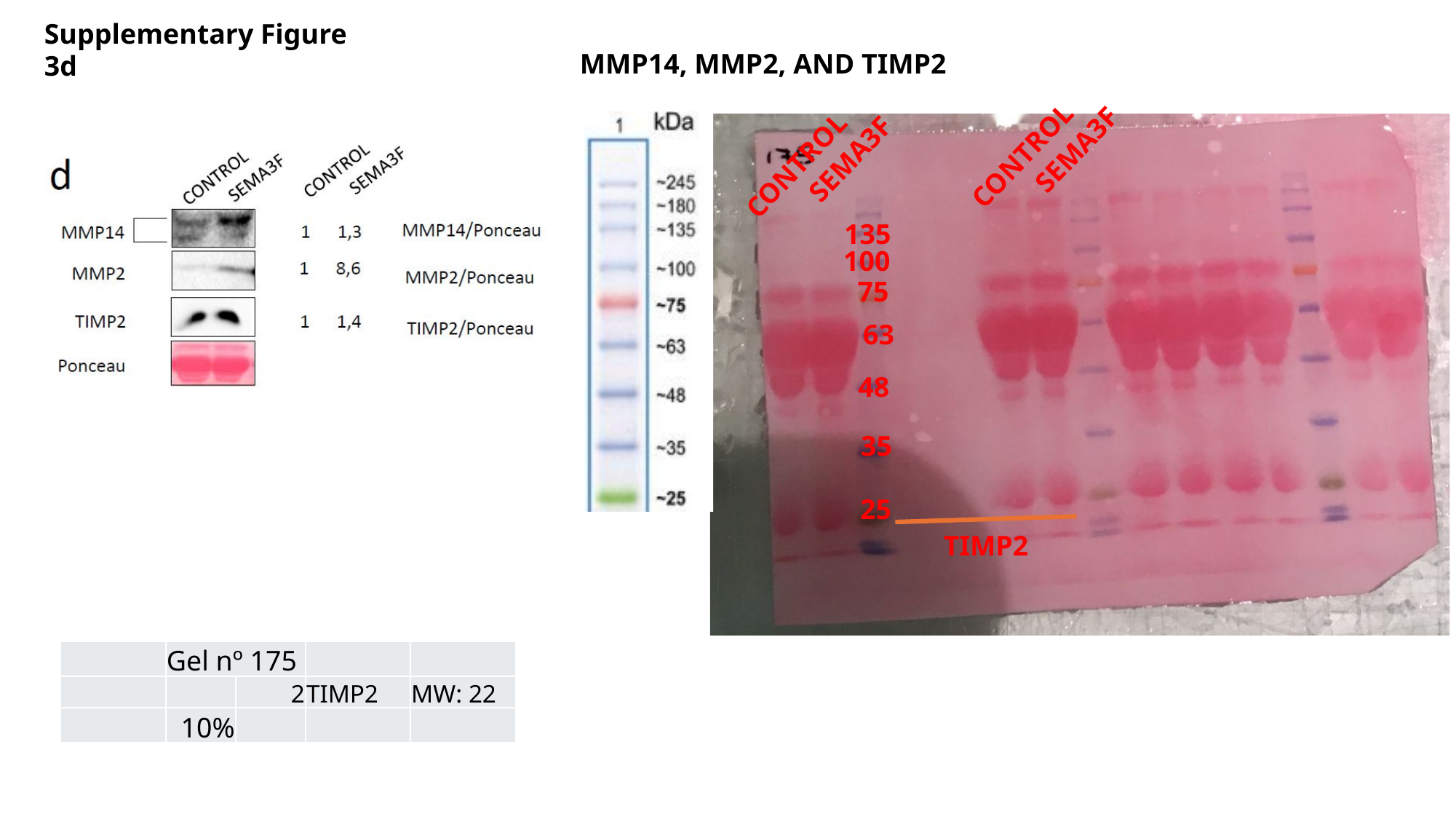

Supplementary Figure 3d
MMP14, MMP2, AND TIMP2
SEMA3F
CONTROL
SEMA3F
CONTROL
135
100
75
63
48
35
25
TIMP2
| | Gel nº 175 | | | |
| --- | --- | --- | --- | --- |
| | | 2 | TIMP2 | MW: 22 |
| | 10% | | | |

## Slide 9
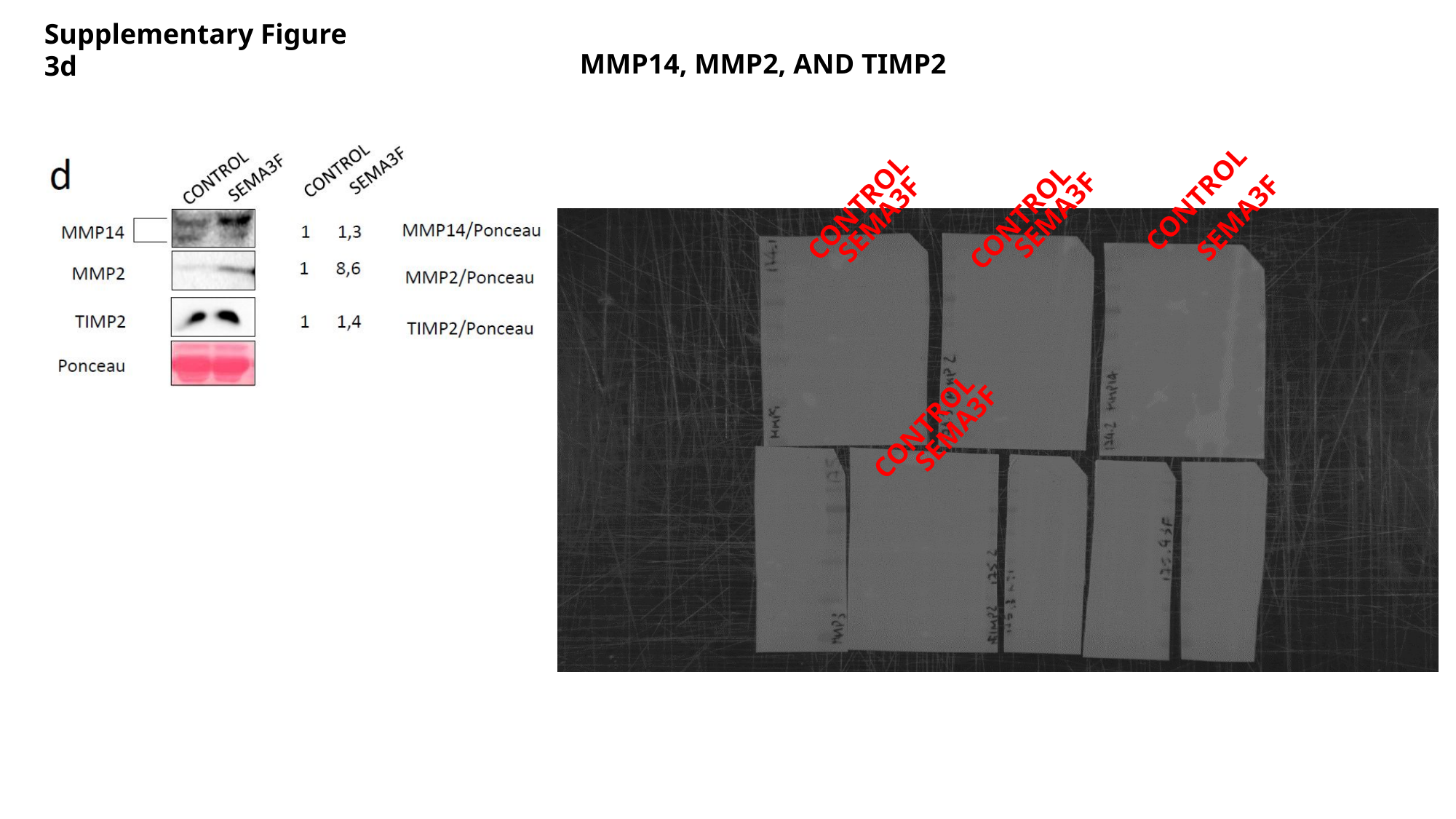

Supplementary Figure 3d
MMP14, MMP2, AND TIMP2
CONTROL
CONTROL
SEMA3F
CONTROL
SEMA3F
SEMA3F
CONTROL
SEMA3F

## Slide 10
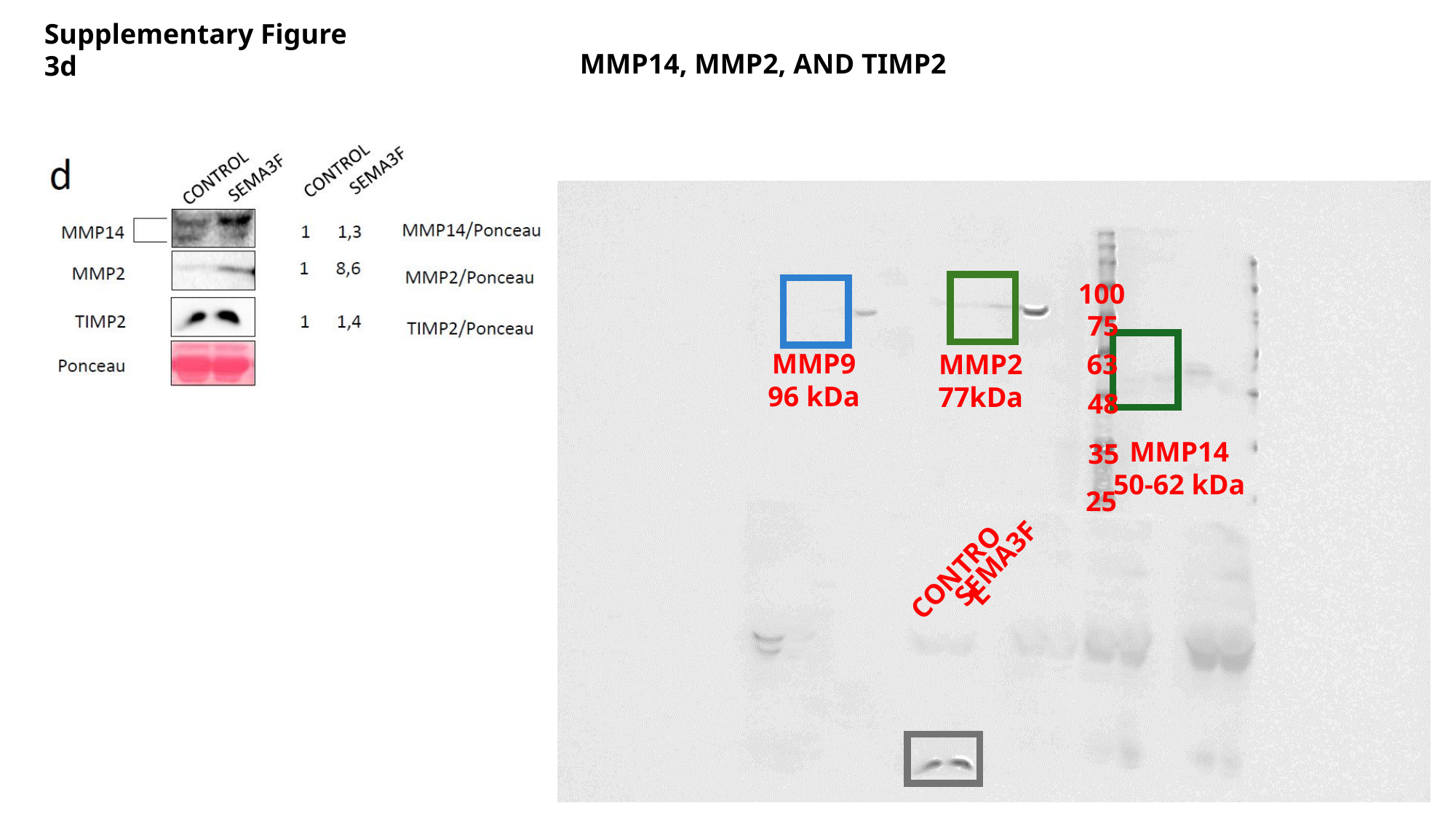

Supplementary Figure 3d
MMP14, MMP2, AND TIMP2
100
75
MMP9
96 kDa
63
MMP2
77kDa
48
MMP14
50-62 kDa
35
25
SEMA3F
CONTROL

## Slide 11
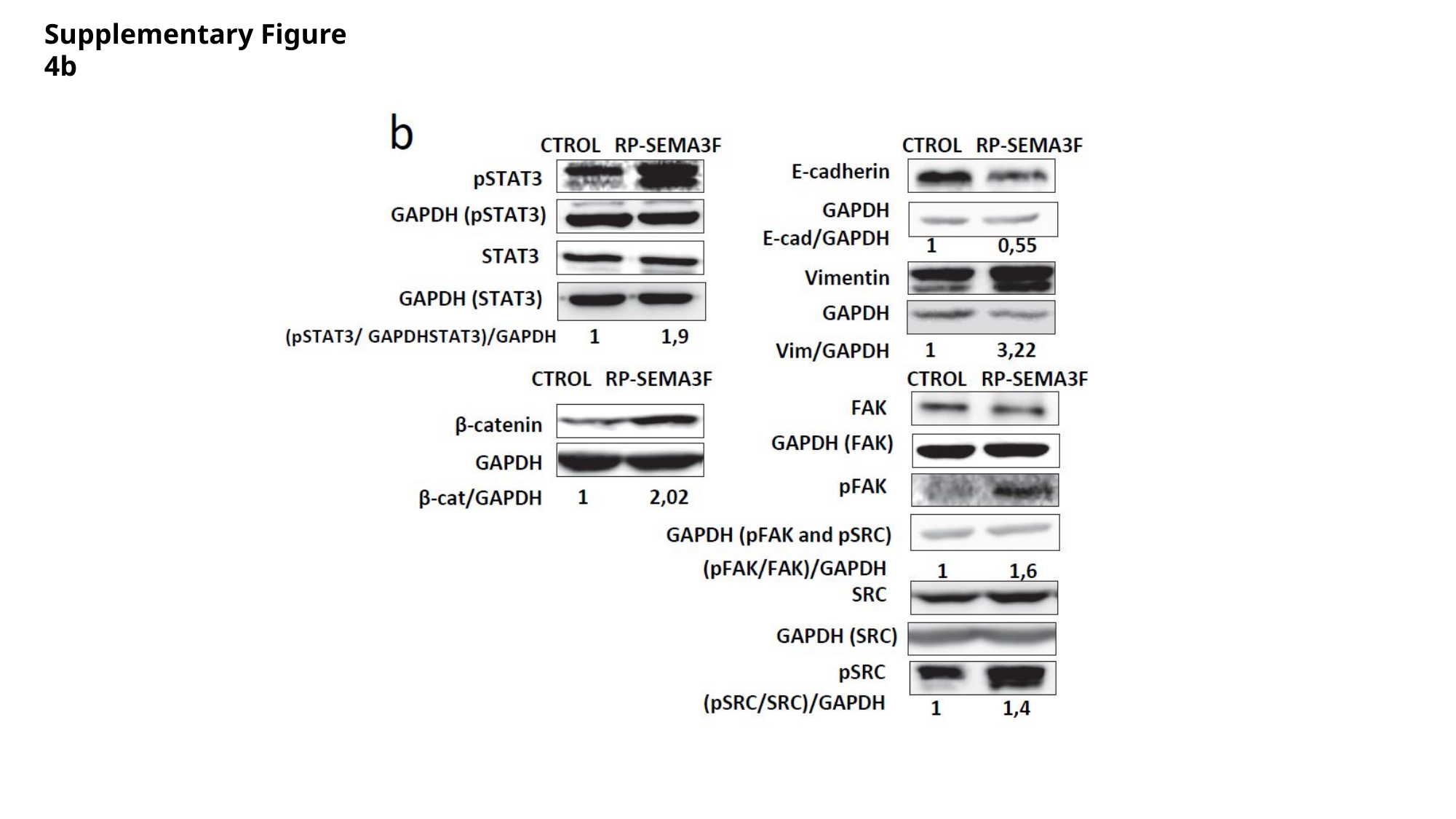

Supplementary Figure 4b

## Slide 12
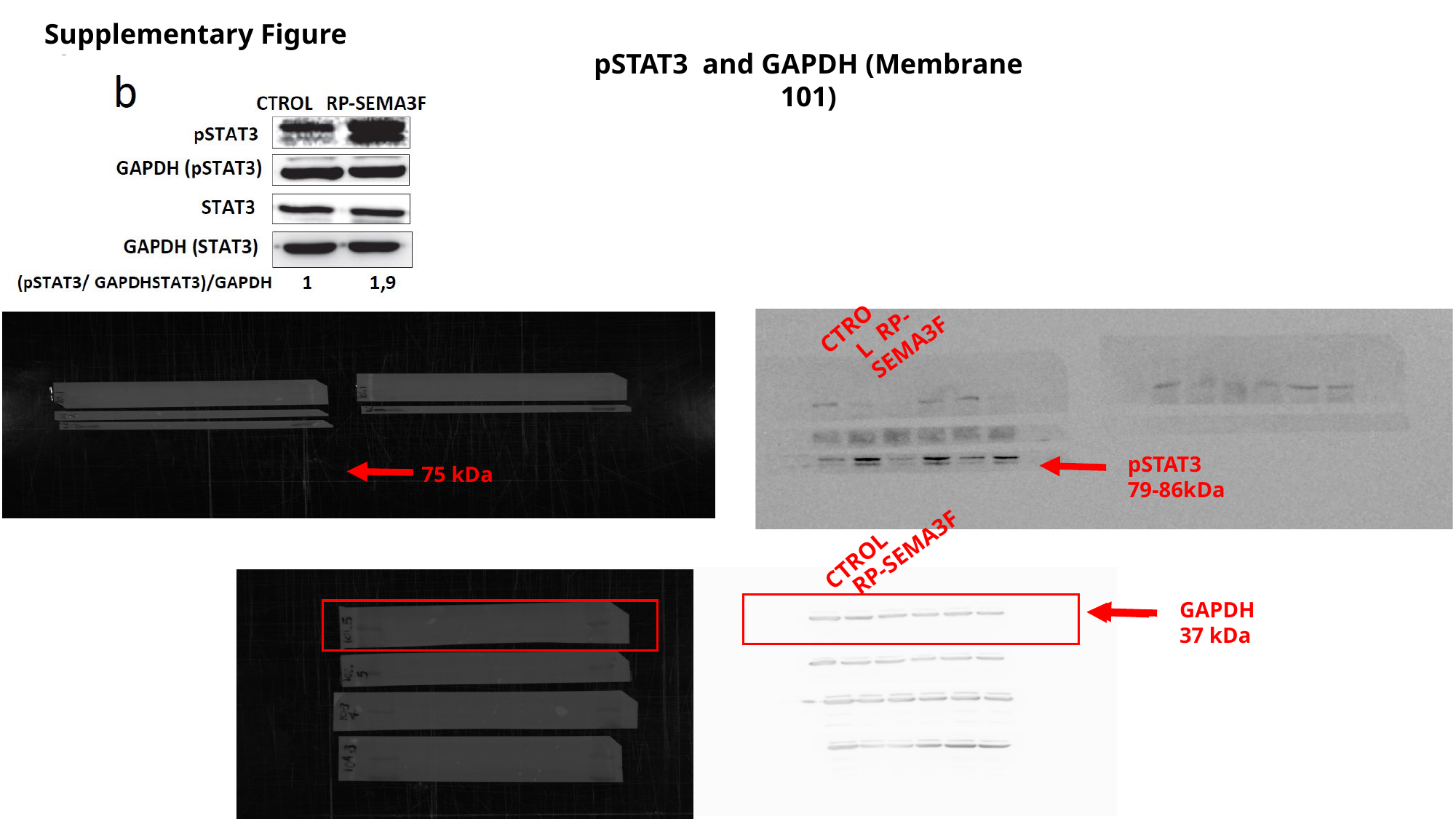

Supplementary Figure 4b
pSTAT3 and GAPDH (Membrane 101)
RP-SEMA3F
CTROL
pSTAT3
79-86kDa
75 kDa
RP-SEMA3F
CTROL
GAPDH
37 kDa

## Slide 13
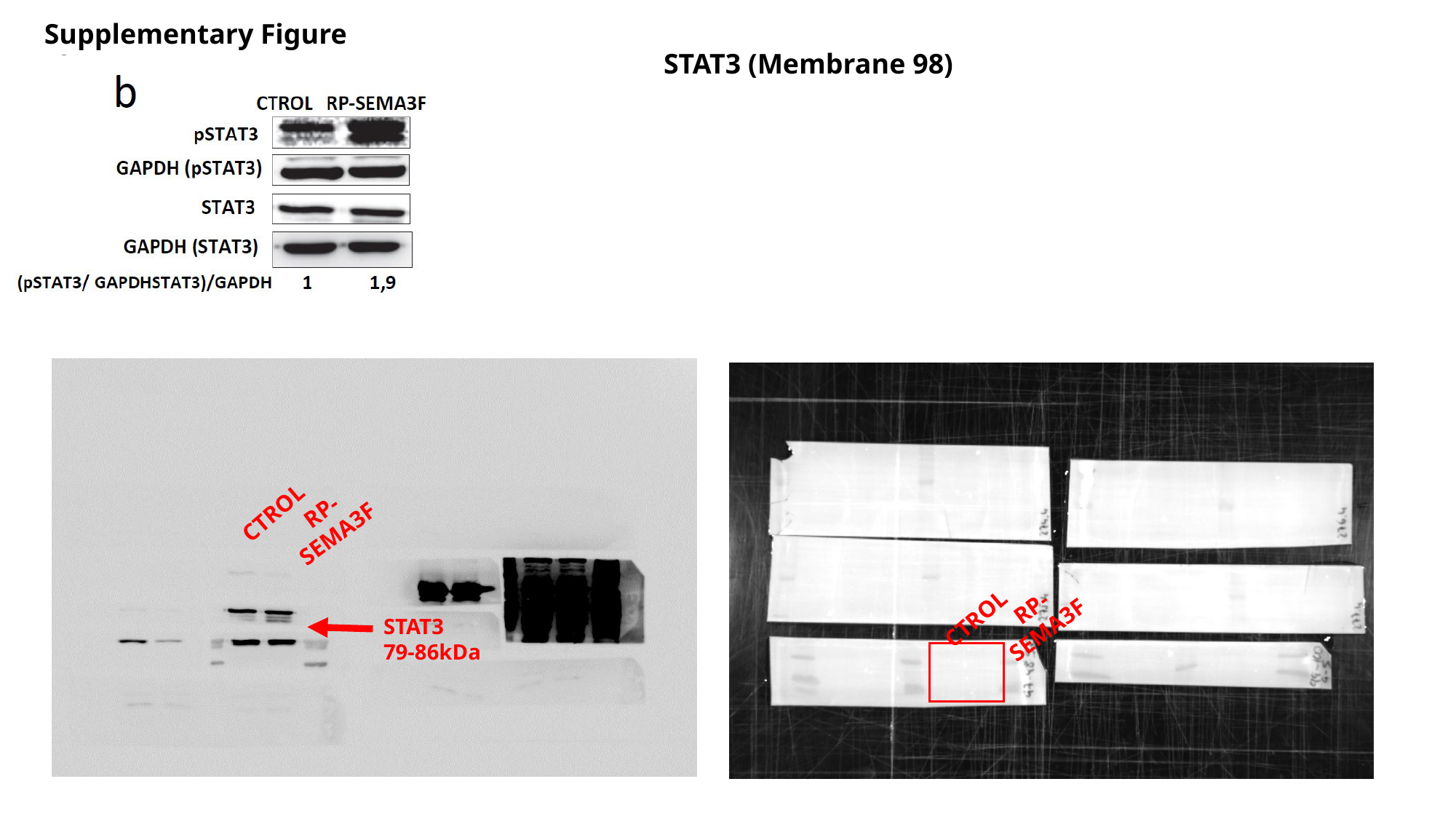

Supplementary Figure 4b
STAT3 (Membrane 98)
RP-SEMA3F
CTROL
RP-SEMA3F
CTROL
STAT3
79-86kDa

## Slide 14
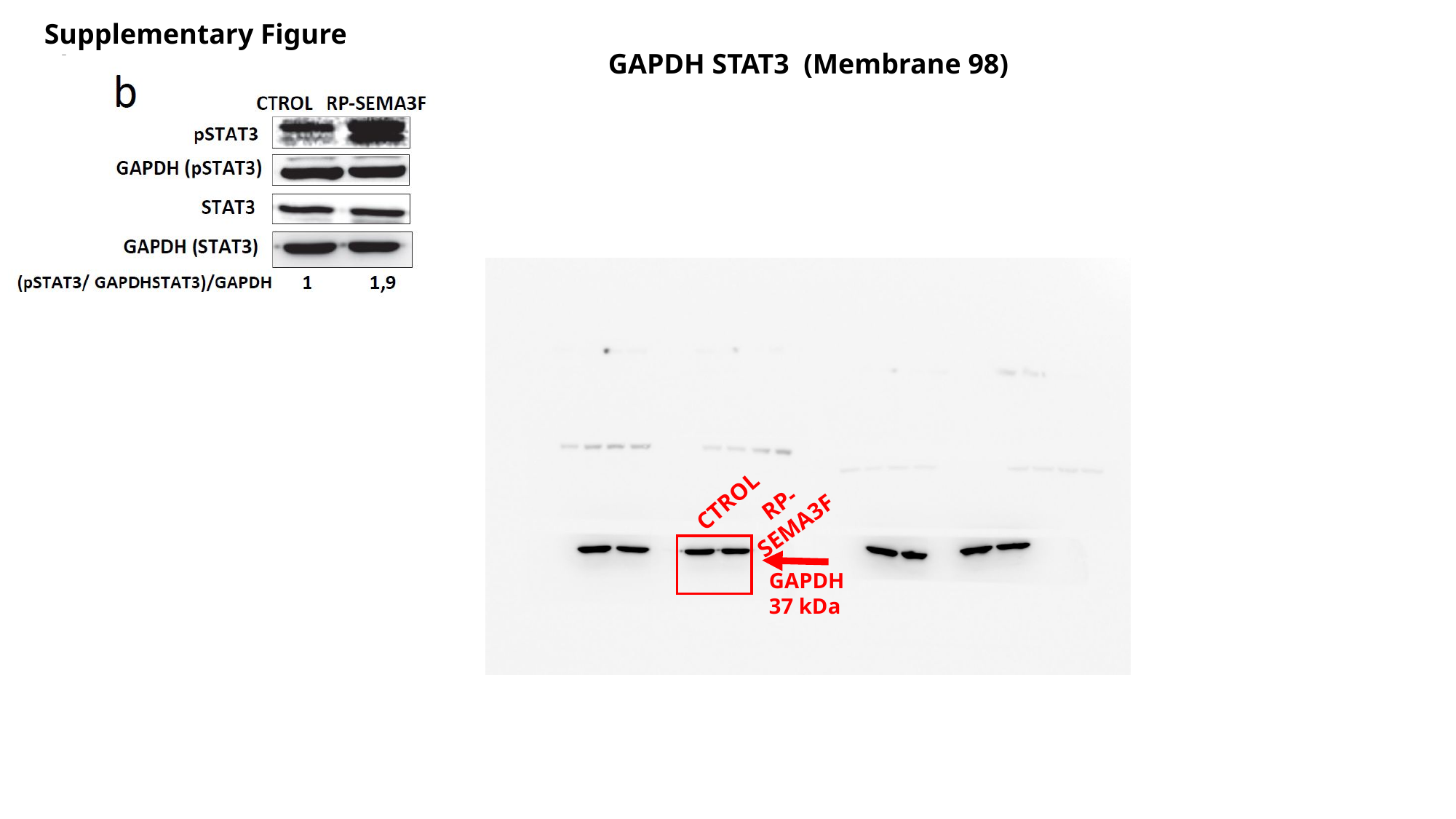

Supplementary Figure 4b
GAPDH STAT3 (Membrane 98)
CTROL
RP-SEMA3F
GAPDH
37 kDa

## Slide 15
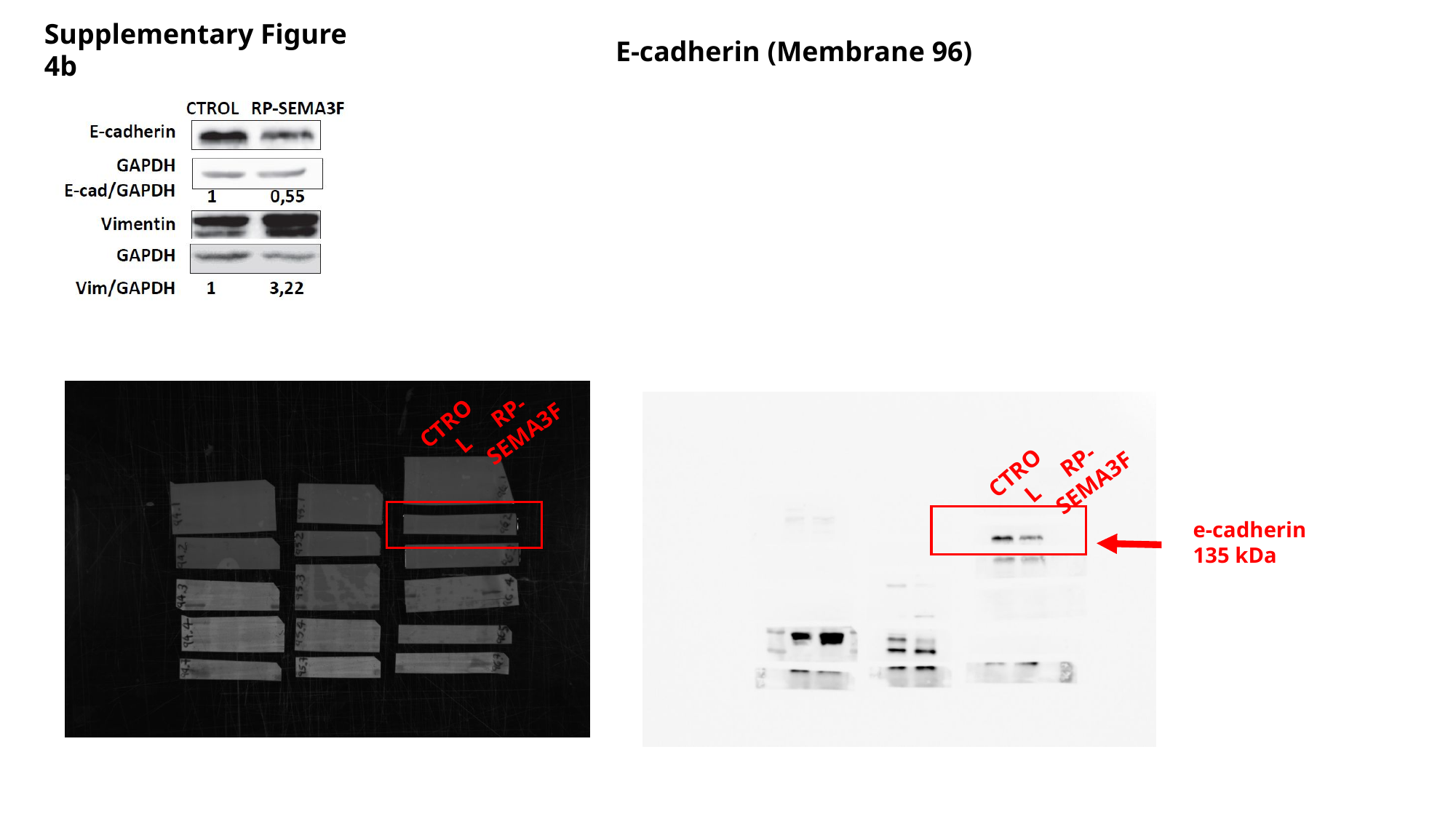

| | Membrane 96 | | | | | | | | | | | | | | | | | | | |
| --- | --- | --- | --- | --- | --- | --- | --- | --- | --- | --- | --- | --- | --- | --- | --- | --- | --- | --- | --- | --- |
| | | | | | | | | | | | | | | | | | | | | |
| | | | | | | | | | | | | | | | | | | | | |
| | | | | | | | | | | | | | | | | | | | | |
| | | | | | | | | | | | | | | | | | | | | |
| | | | | | | | | | | | | | | | | | | | | |
| | | | | | | | | | | | | | | | | | | | | |
| | | | | | | | | | | | | | | | | | | | | |
| | | | | | | | | | | | | | | | | | | | | |
| | | | | | | | | | | | | | | | | | | | | |
| | | | | | | | | | | | | | | | | | | | | |
| | | | | | | | | | | | | | | | | | | | | |
| | | | | | | | | | | | | | | | | | | | | |
| | | | | | | | | | | | | | | | | | | | | |
| | | | | | | | | | | | | | | | | | | | | |
| | | | | | | | | | | | | | | | | | | | | |
| | | | | | | | | | | | | | | | | | | | | |
| | | | | | | | | | | | | | | | | | | | | |
| | | | | | | | | | | | | | | | | | | | | |
| | | | | | | | | | | | | | | | | | | | | |
Supplementary Figure 4b
E-cadherin (Membrane 96)
RP-SEMA3F
CTROL
RP-SEMA3F
CTROL
e-cadherin
135 kDa

## Slide 16
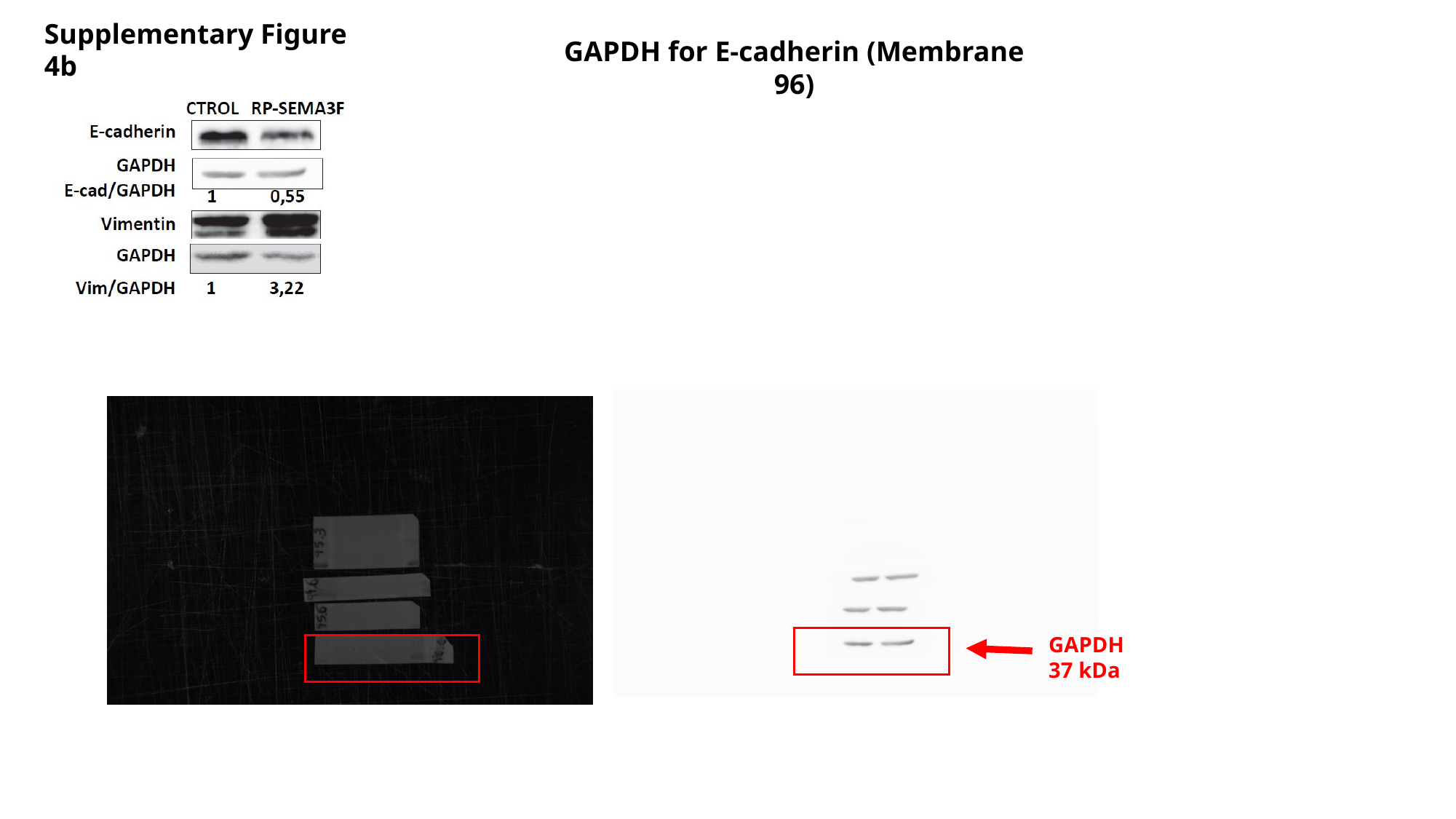

| | Membrane 96 | | | | | | | | | | | | | | | | | | | |
| --- | --- | --- | --- | --- | --- | --- | --- | --- | --- | --- | --- | --- | --- | --- | --- | --- | --- | --- | --- | --- |
| | | | | | | | | | | | | | | | | | | | | |
| | | | | | | | | | | | | | | | | | | | | |
| | | | | | | | | | | | | | | | | | | | | |
| | | | | | | | | | | | | | | | | | | | | |
| | | | | | | | | | | | | | | | | | | | | |
| | | | | | | | | | | | | | | | | | | | | |
| | | | | | | | | | | | | | | | | | | | | |
| | | | | | | | | | | | | | | | | | | | | |
| | | | | | | | | | | | | | | | | | | | | |
| | | | | | | | | | | | | | | | | | | | | |
| | | | | | | | | | | | | | | | | | | | | |
| | | | | | | | | | | | | | | | | | | | | |
| | | | | | | | | | | | | | | | | | | | | |
| | | | | | | | | | | | | | | | | | | | | |
| | | | | | | | | | | | | | | | | | | | | |
| | | | | | | | | | | | | | | | | | | | | |
| | | | | | | | | | | | | | | | | | | | | |
| | | | | | | | | | | | | | | | | | | | | |
| | | | | | | | | | | | | | | | | | | | | |
Supplementary Figure 4b
GAPDH for E-cadherin (Membrane 96)
GAPDH
37 kDa

## Slide 17
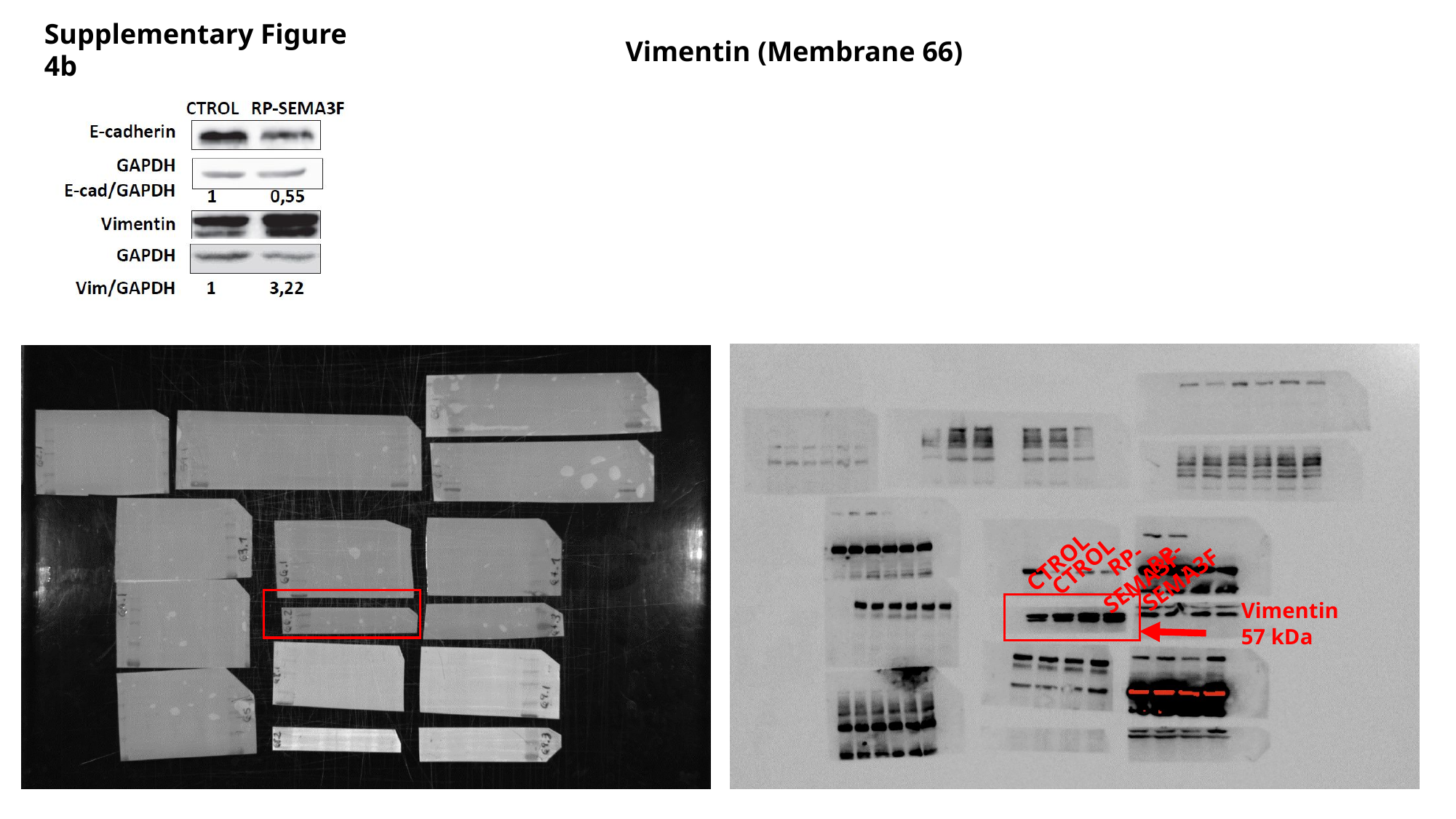

Supplementary Figure 4b
Vimentin (Membrane 66)
RP-SEMA3F
RP-SEMA3F
CTROL
CTROL
Vimentin
57 kDa

## Slide 18
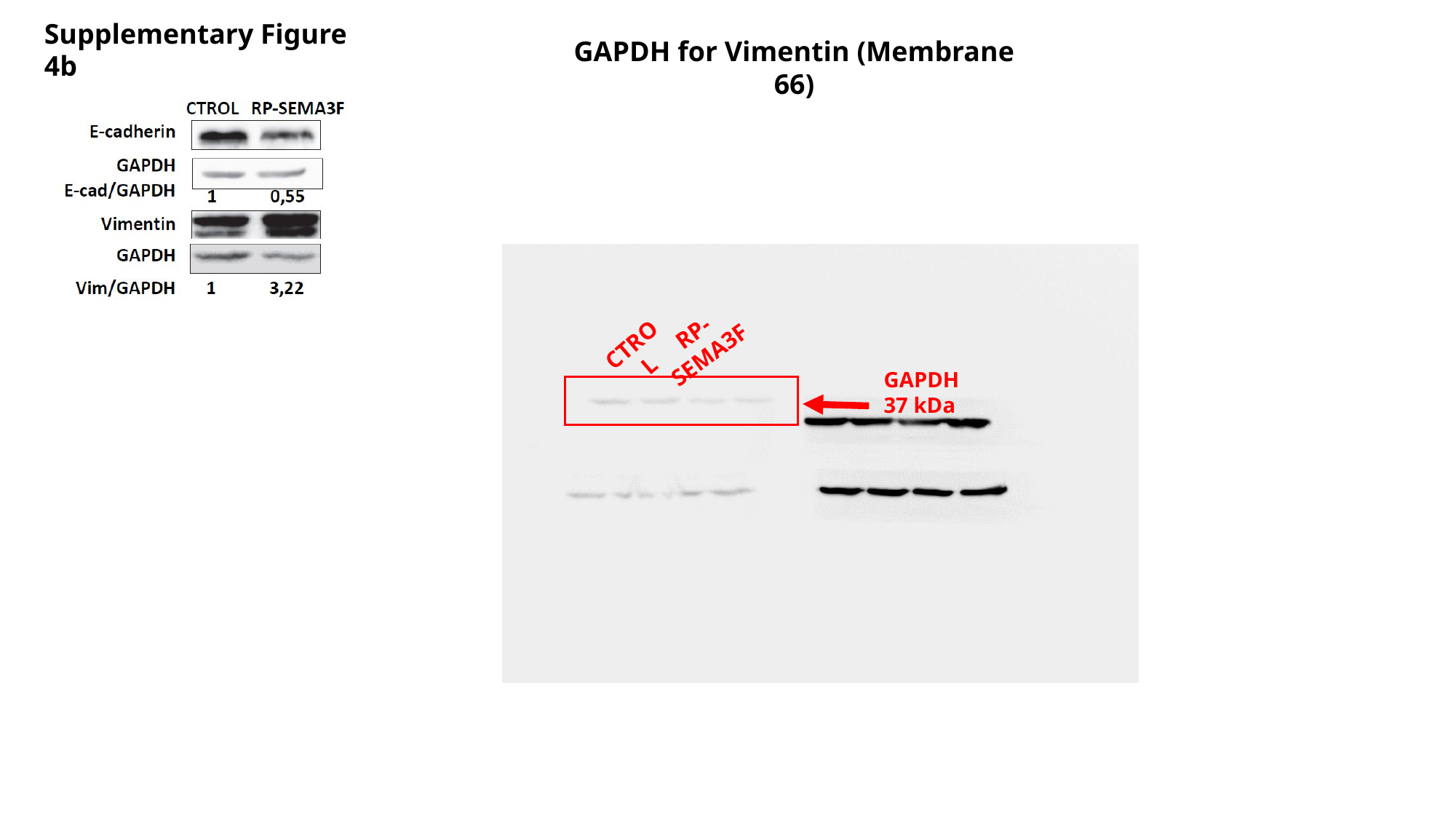

Supplementary Figure 4b
GAPDH for Vimentin (Membrane 66)
RP-SEMA3F
CTROL
GAPDH
37 kDa

## Slide 19
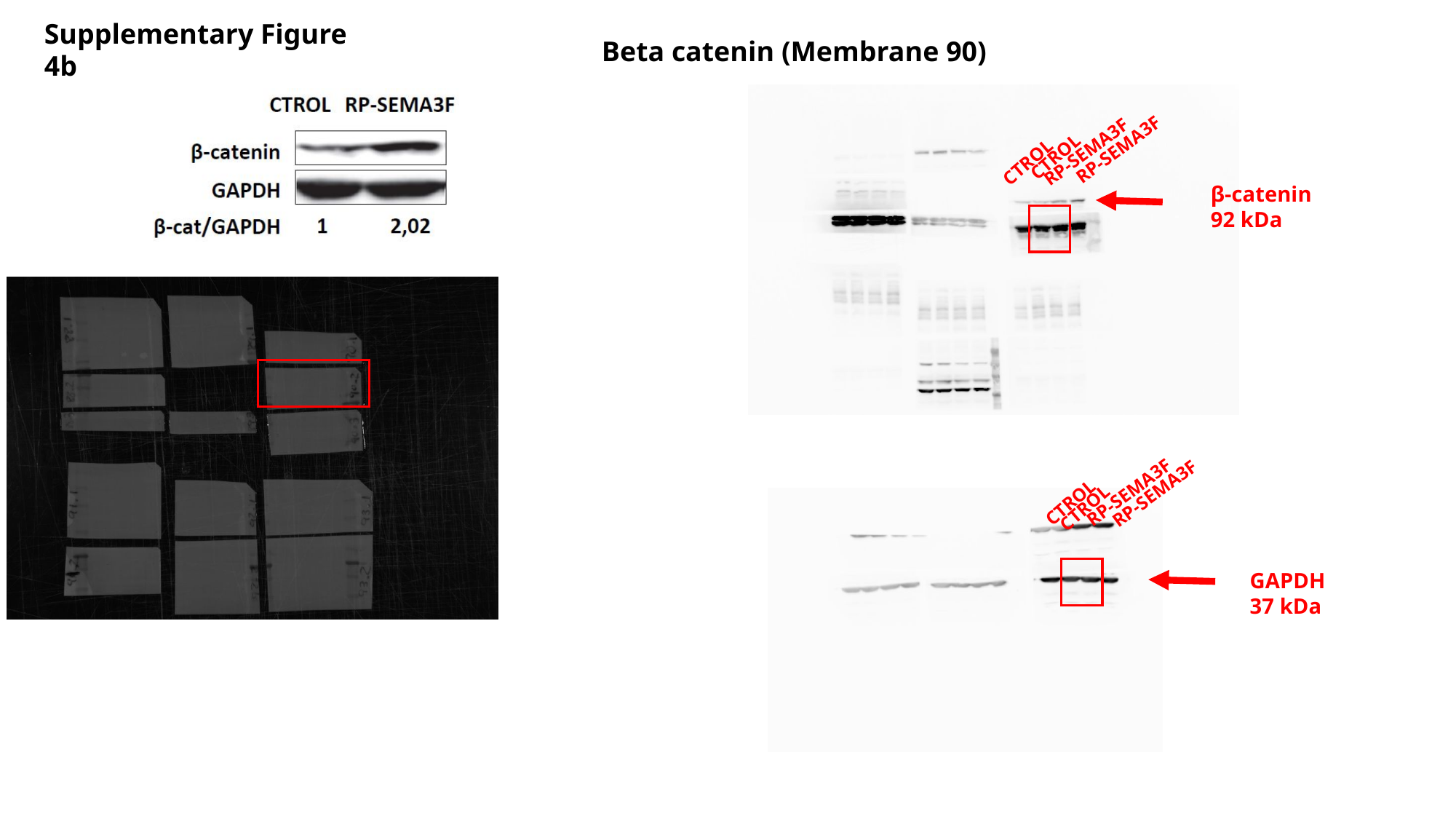

Supplementary Figure 4b
Beta catenin (Membrane 90)
RP-SEMA3F
RP-SEMA3F
CTROL
CTROL
β-catenin
92 kDa
RP-SEMA3F
RP-SEMA3F
CTROL
CTROL
GAPDH
37 kDa

## Slide 20
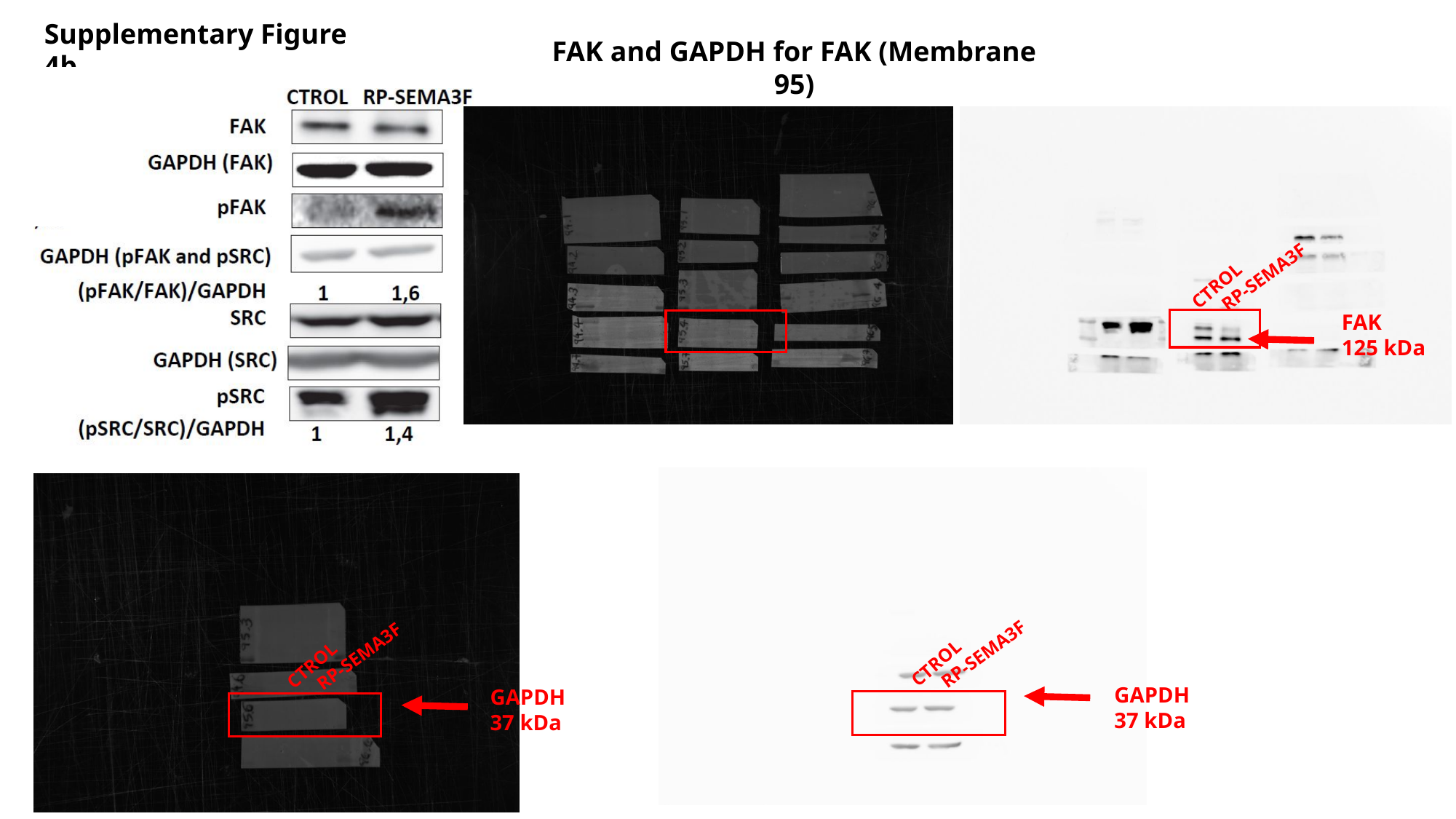

Supplementary Figure 4b
FAK and GAPDH for FAK (Membrane 95)
RP-SEMA3F
CTROL
FAK
125 kDa
RP-SEMA3F
RP-SEMA3F
CTROL
CTROL
GAPDH
37 kDa
GAPDH
37 kDa

## Slide 21
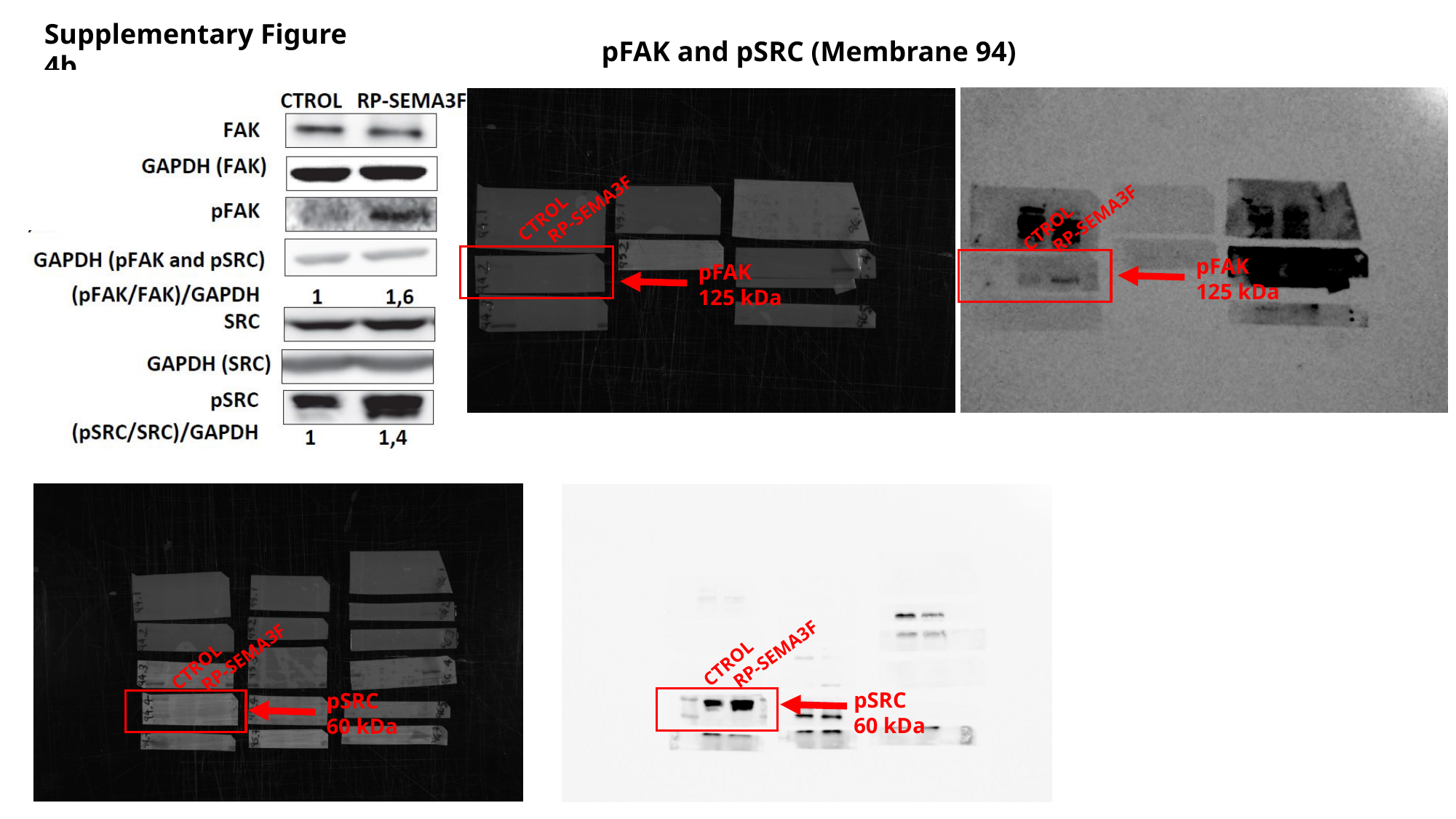

Supplementary Figure 4b
pFAK and pSRC (Membrane 94)
RP-SEMA3F
CTROL
RP-SEMA3F
CTROL
pFAK
125 kDa
pFAK
125 kDa
RP-SEMA3F
RP-SEMA3F
CTROL
CTROL
pSRC
60 kDa
pSRC
60 kDa

## Slide 22
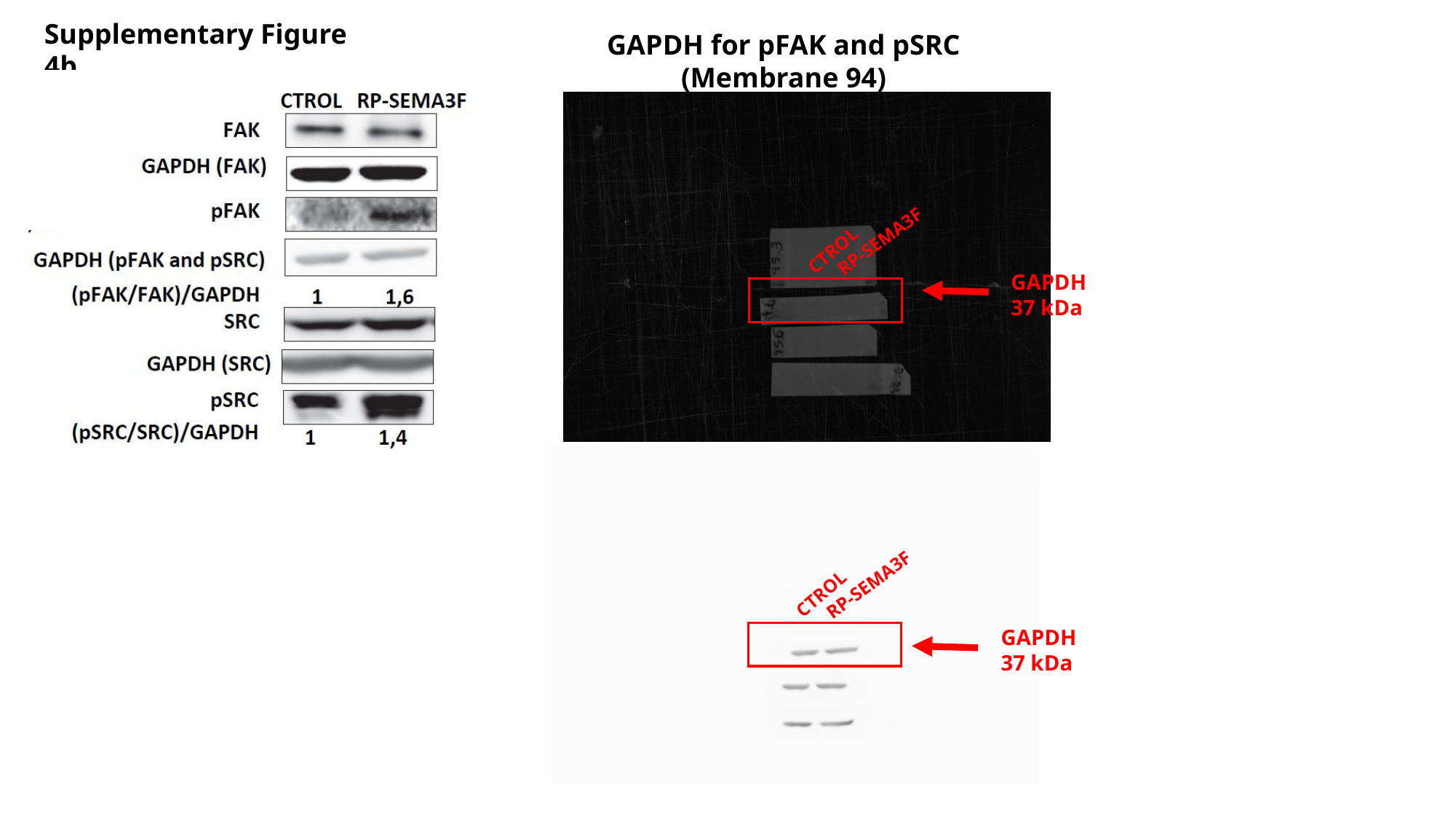

Supplementary Figure 4b
GAPDH for pFAK and pSRC (Membrane 94)
RP-SEMA3F
CTROL
GAPDH
37 kDa
RP-SEMA3F
CTROL
GAPDH
37 kDa

## Slide 23
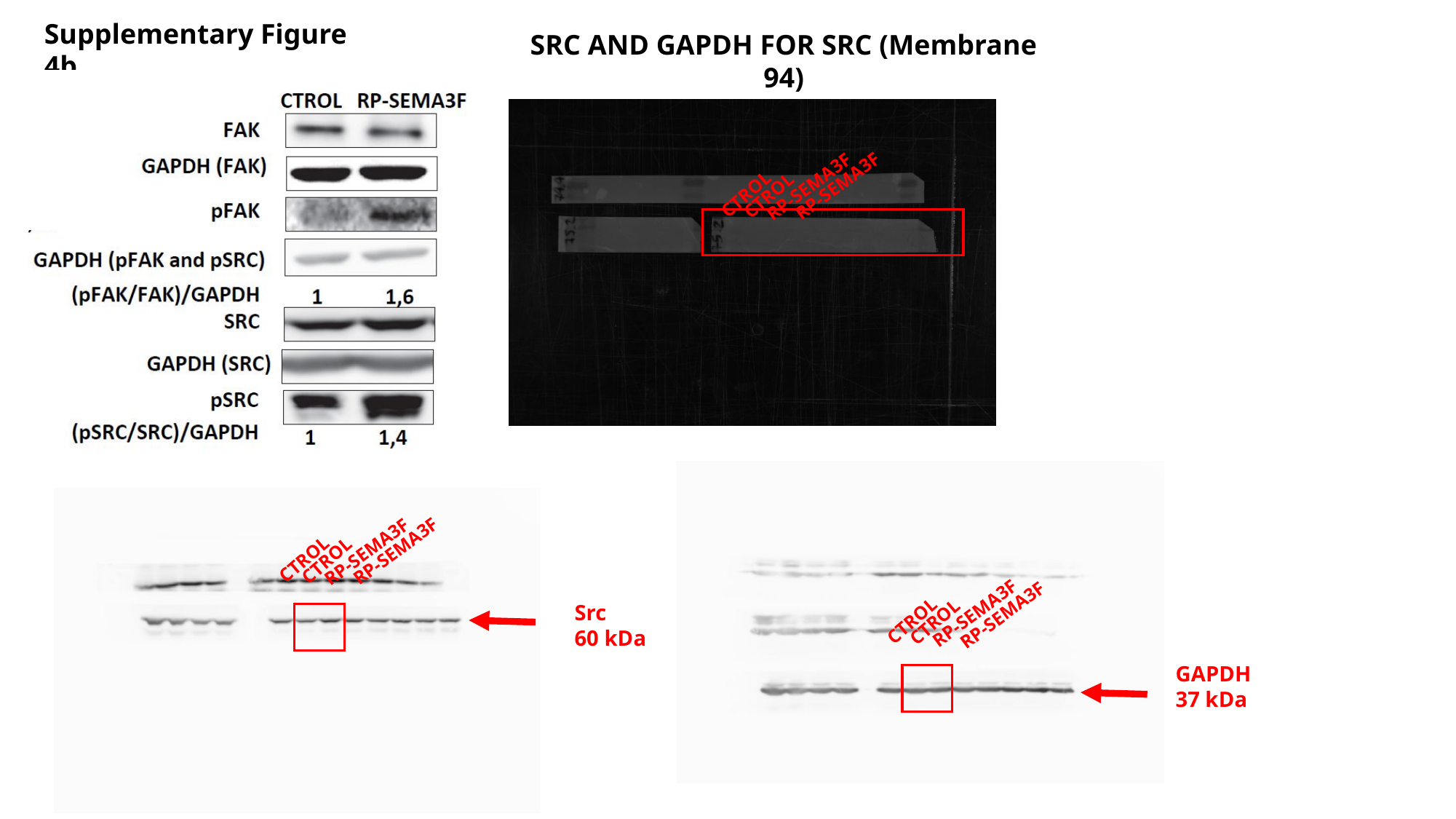

Supplementary Figure 4b
SRC AND GAPDH FOR SRC (Membrane 94)
RP-SEMA3F
RP-SEMA3F
CTROL
CTROL
RP-SEMA3F
RP-SEMA3F
CTROL
CTROL
Src
60 kDa
RP-SEMA3F
RP-SEMA3F
CTROL
CTROL
GAPDH
37 kDa

## Slide 24
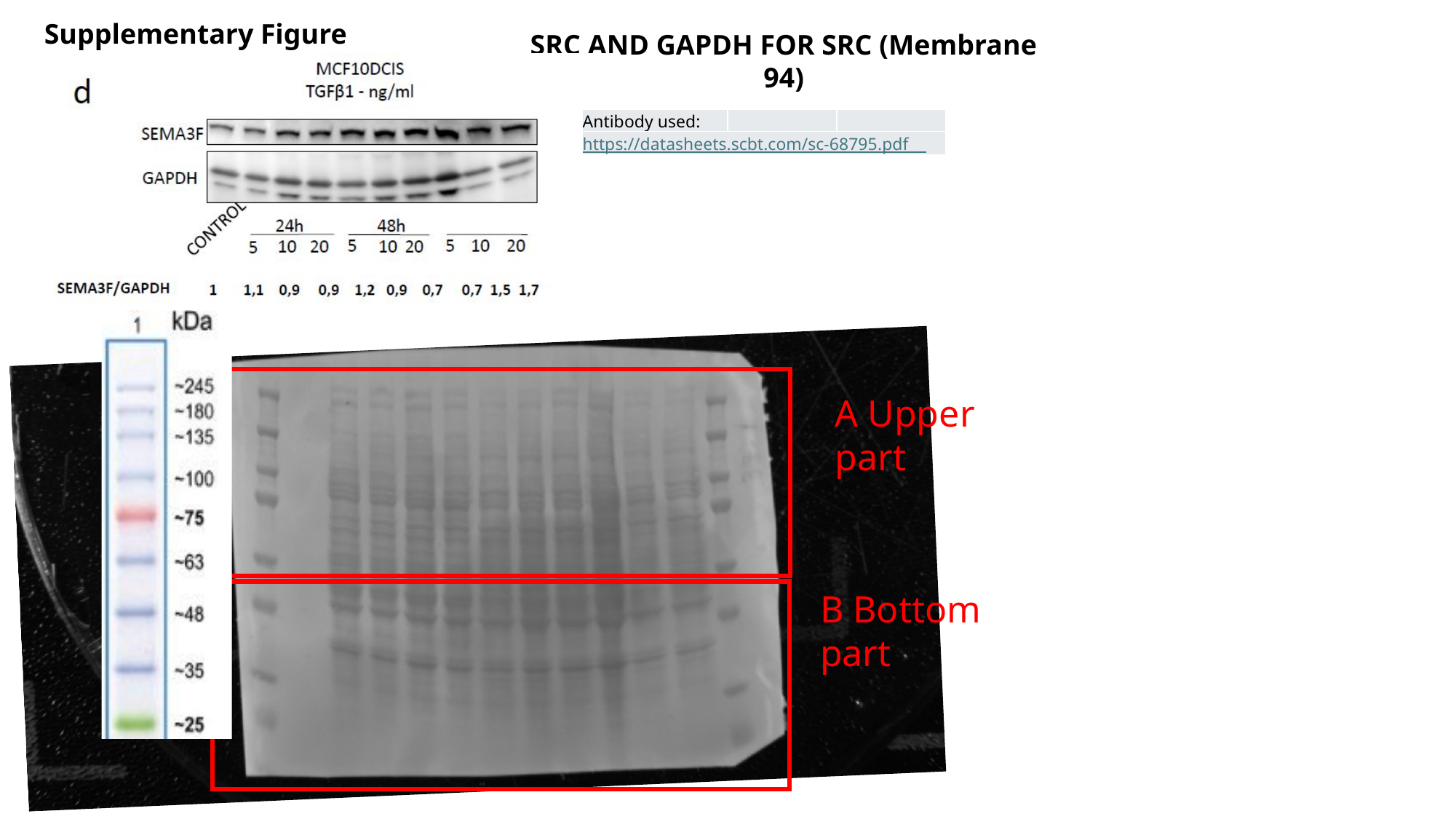

Supplementary Figure 4d
SRC AND GAPDH FOR SRC (Membrane 94)
| Antibody used: | | |
| --- | --- | --- |
| https://datasheets.scbt.com/sc-68795.pdf | | |
A Upper part
B Bottom part

## Slide 25
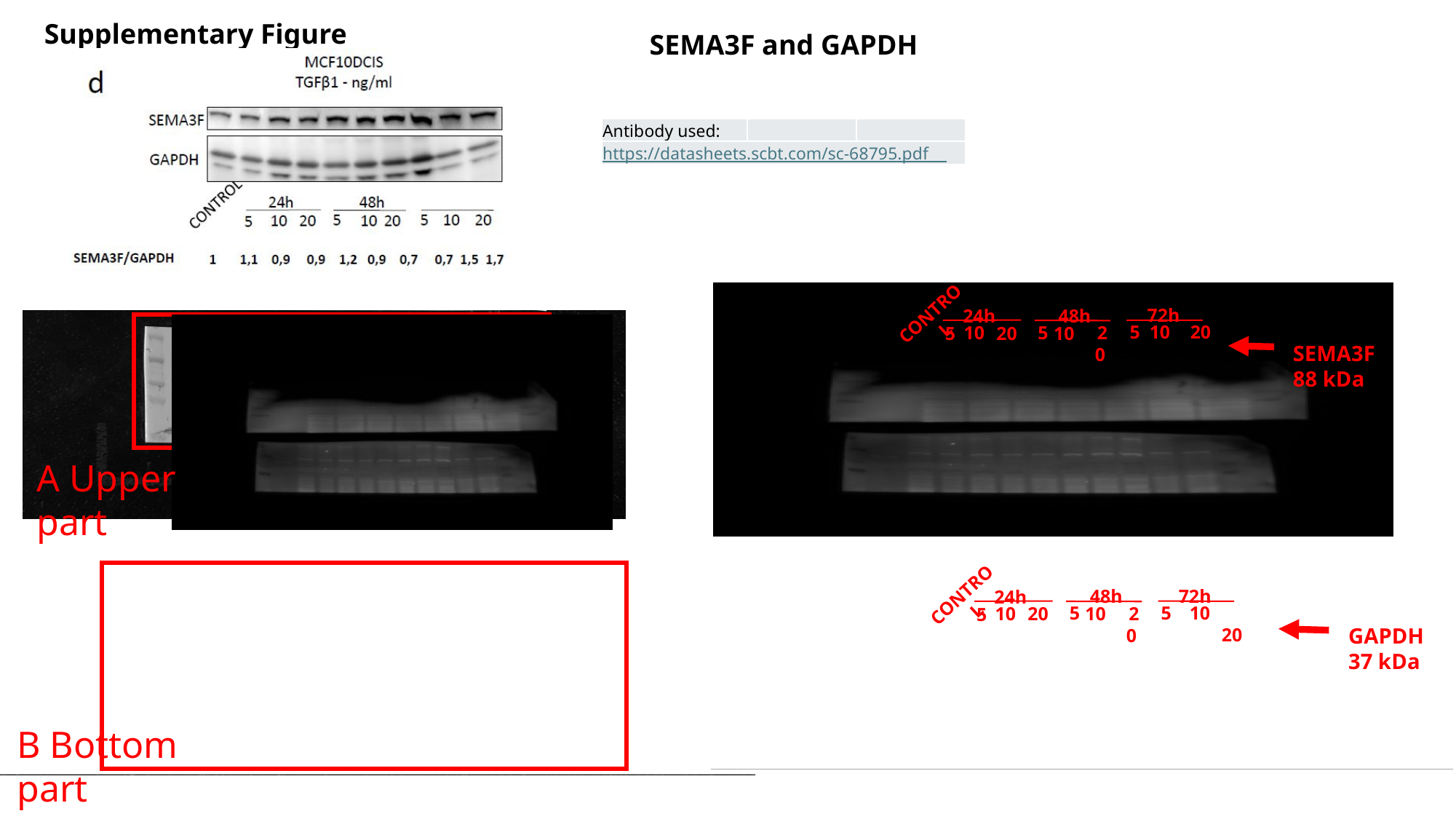

Supplementary Figure 4d
SEMA3F and GAPDH
| Antibody used: | | |
| --- | --- | --- |
| https://datasheets.scbt.com/sc-68795.pdf | | |
SEMA3F
88 kDa
72h
CONTROL
48h
24h
20
5
10
5
10
20
20
10
5
A Upper part
GAPDH
37 kDa
72h
CONTROL
48h
24h
10
 20
5
5
10
20
20
10
5
B Bottom part

## Slide 26
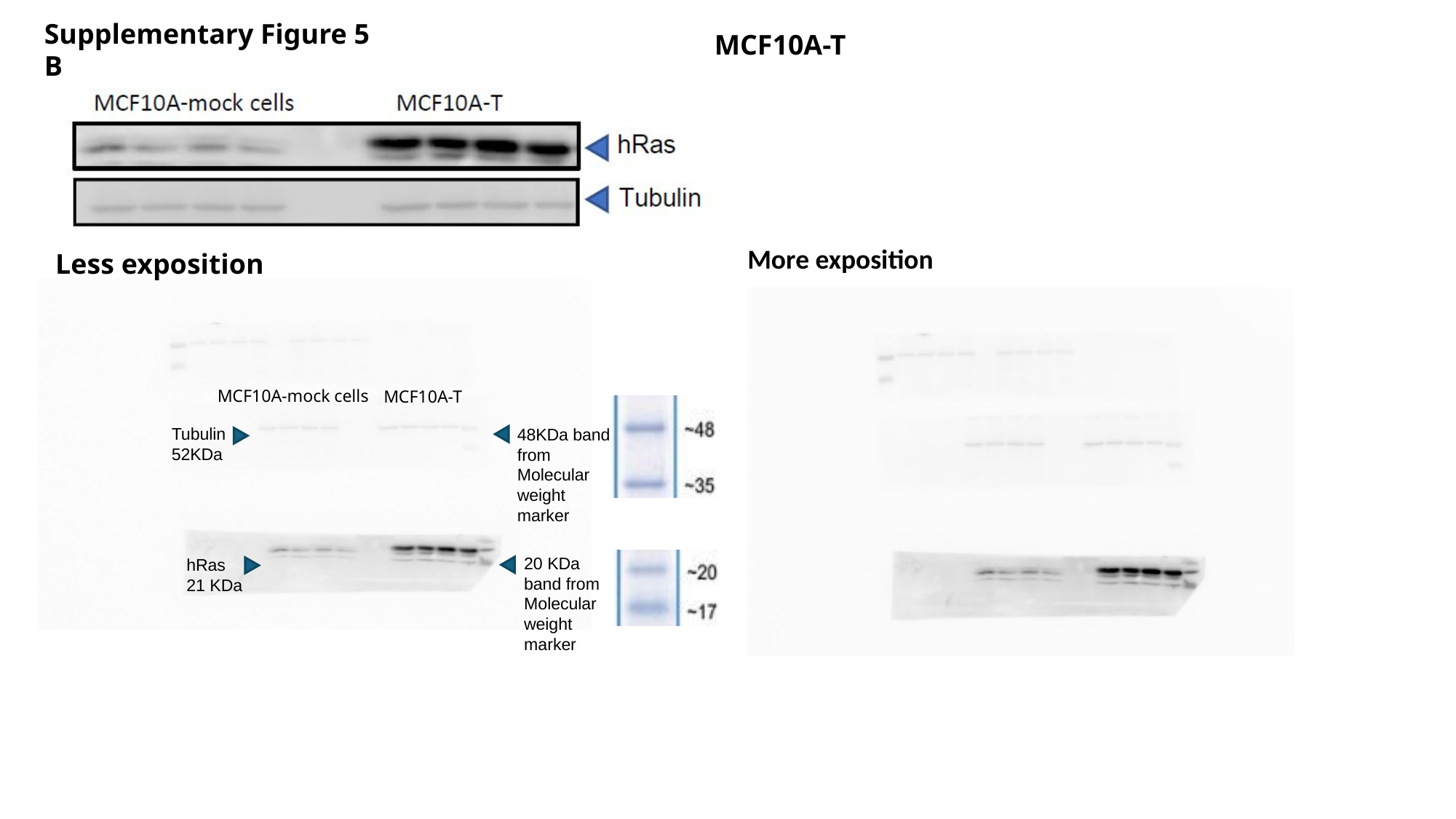

Supplementary Figure 5 B
MCF10A-T
More exposition
Less exposition
MCF10A-mock cells
MCF10A-T
Tubulin
52KDa
48KDa band from Molecular weight marker
20 KDa
band from Molecular weight marker
hRas
21 KDa
