# Supplementary material for: The SEMA3F-NRP1/NRP2 axis is a key factor in the acquisition of invasive traits in in situ breast ductal carcinoma
Source: Breast Cancer Res. 2024 Aug 13;26:122. doi: 10.1186/s13058-024-01871-0 (PMC11320849; doi:10.1186/s13058-024-01871-0)
Supplement: Supplementary file 7 — Supplementary Material 7. [file 13058_2024_1871_MOESM7_ESM.docx]

| **NETRIN** | **HISTAMINE** | **NEUROPILIN** | **SNARE**  **(Complex)** | **INDEPENDENT GENES** |
| --- | --- | --- | --- | --- |
| DCC | HRH1 | NRP1 | SNAP23 | GRID1 |
| NEO1 (R) | HRH2 | NRP2 | SNAP25 | NGFR |
| UNC5A | HRH3 | SEMA4D | VAMP1 | CNTFR |
| NTN1 | HRH4 | SEMA3A | VAMP2 | SLC17AT |
|  | HDC | SEMA3B | STX1A | ADORA1 |
|  |  | SEMA3C | STX1B | APP |
|  |  | SEMA3D | STXBP1 |  |
|  |  | SEMA3F | STXBP2 |  |
|  |  | SEMA3G | STX2 |  |
|  |  | PLXNA1 | STX3 |  |
|  |  | PLXNA1 | STX6 |  |
|  |  | PLXNA3 | SYT1 |  |
|  |  | PLXNA4 | CPLX1 |  |
|  |  | PLXND1 |  |  |
|  |  |  |  |  |
| **Supplementary Table 1.** List of neurogenes used for differential genetic analysis, categorized into five groups based on their interrelations. This compilation stems from the previous bioinformatics analysis performed by our research team^15^. | | | | |
|  |  |  |  |  |
|  |  |  |  |  |
|  |  |  |  |  |
|  |  |  |  |  |
|  |  |  |  |  |
|  |  |  |  |  |
|  |  |  |  |  |
|  |  |  |  |  |
|  |  |  |  |  |
|  |  |  |  |  |
|  |  |  |  |  |
|  |  |  |  |  |
|  |  |  |  |  |
|  |  |  |  |  |
|  |  |  |  |  |
|  |  |  |  |  |

| Gene | Reference |  | Gene | Reference |
| --- | --- | --- | --- | --- |
| ACTB | Hs00946916_m1 |  | **SEMA3A** | Hs00173810_m1 |
| ADORA1 | Hs00181231_m1 |  | **SEMA3B** | Hs00190328_m1 |
| APP | Hs00169098_m1 |  | **SEMA3C** | Hs00989373_m1 |
| CD10 | Hs00153510_m1 |  | **SEMA3D** | Hs00380877_m1 |
| CK14 | Hs00265033_m1 |  | **SEMA3F** | Hs00188273_m1 |
| CK19 | Hs00761767_s1 |  | **SEMA3G** | Hs00220101_m1 |
| CNTFR | Hs00181798_m1 |  | **SEMA4D** | Hs00925667_m1 |
| CPLX1 | Hs00362510_m1 |  | **SLC17A7** | Hs00220404_m1 |
| DCC | Hs00180437_m1 |  | **SMAD2** | Hs00998187_m1 |
| CDH1 | Hs01023894_m1 |  | **SNAIL1** | Hs00195591_m1 |
| FN1 | Hs00365052_m1 |  | **SNAIL2** | Hs00950344_m1 |
| GRID1 | Hs00324946_m1 |  | **SNAP23** | Hs01047496_m1 |
| HDC | Hs01113376_m1 |  | **SNAP25** | Hs00938962_m1 |
| HRH1 | Hs00911670_s1 |  | **STX1A** | Hs00270282_m1 |
| HRH2 | Hs00254569_s1 |  | **STX1B** | Hs01041315_m1 |
| HRH2 | Hs00254569_s1 |  | **STX2** | Hs00181827_m1 |
| HRH3 | Hs00200610_m1 |  | **STX3** | Hs00188210_m1 |
| HRH4 | Hs00222094_m1 |  | **STX6** | Hs01057343_m1 |
| NEO1 | Hs00924151_m1 |  | **STXBP1** | Hs01119036_m1 |
| NGFR | Hs00609976_m1 |  | **STXBP2** | Hs00199557_m1 |
| NRP1 | Hs00826128_m1 |  | **SYT1** | Hs00194572_m1 |
| NRP2 | Hs00187290_m1 |  | **TWIST** | Hs01675818_s1 |
| NTN1 | Hs00924151_m1 |  | **UNC5A** | Hs00293338_m1 |
| PLXNA1 | Hs00413698_m1 |  | **VAMP1** | Hs04399177_m1 |
| PLXNA2 | Hs00300697_m1 |  | **VAMP2** | Hs00360269_m1 |
| PLXNA3 | Hs00250178_m1 |  | **VIM** | Hs00185584_m1 |
| PLXNA4 | Hs00297356_m1 |  |  |  |
| PLXND1 | Hs00892410_m1 |  |  |  |

**Supplementary table 2.** List of TaqMan®primers and probes used for qPCR (Applied Biosystems™).

| Primary antibody | Brand | Cat# | Origin | Dilution | Appl* |
| --- | --- | --- | --- | --- | --- |
| α-Tubulin | Cell Signalling | 2144S | Rabbit | 1/5000 | WB |
| α-Tubulin | Cell Signalling | 3873S | Mouse | 1/1000 | WB |
| β-catenin | Cell Signalling | 9587 | Rabbit | 1/100  1/1000 | IF  WB |
| CK14 | BioLegend | 906004 | Chicken | 1/500 | IF |
| CK14 | Abcam | Ab9220 | Mouse | 1/20 | IF |
| CK19 | Abcam | Ab7754 | Mouse | 1/100 | IF |
| Cleaved-Caspase 3 | Cell Signalling | 9664S | Rabbit | 1/300 | IF |
| E-cadherin | Cell Signalling | 3195S | Rabbit | 1/200  1/1000 | IF  WB |
| FAK | Cell Signalling | 3285T | Rabbit | 1/1000 | WB |
| GAPDH | Cell Signalling | 2118S | Rabbit | 1/1000 | WB |
| Integrin α6/CD49f | BD Pharmingen | 555734 | Rat | 1/200 | IF |
| Laminin | Abcam | Ab11575 | Rabbit | 1/50 | IF |
| MMP2 | Abcam | Ab37150 | Rabbit | 1/1000 | WB |
| MMP14 | Millipore | MAB3328 | Mouse | 1/1000 | WB |
| NRP1 | Abcam | 25998 | Rabbit | 1/200  1/1000 | IF  WB |
| NRP2 | R&D Systems | AF2215 | Goat | 1/50 | IF |
| p63 | Abcam | Ab735 | Mouse | 1/50 | IF |
| P63 | Santa Cruz | 8431 | Mouse | 1/50 | IF |
| pFAK | Cell Signalling | 3283S | Rabbit | 1/1000 | WB |
| pSMAD2 | Cell Signalling | 3108S | Rabbit | 1/1000 | WB |
| pSRC | Cell Signalling | 6943T | Rabbit | 1/1000 | WB |
| pSTAT3 | Cell Signalling | 9131L | Rabbit | 1/1000 | WB |
| SEMA3F | Santa Cruz | 68795 | Goat | 1/50  1/1000 | IF  WB |
| SEMA3F | R&D Systems | AF3237 | Sheep | 1/50  1/1000 | IF  WB |
| SMAD2 | Cell Signalling | 5339S | Rabbit | 1/1000 | WB |
| SMAD4 | Cell Signalling | 38454 | Rabbit | 1/1000 | WB |
| SRC | Cell Signalling | 2109T | Rabbit | 1/1000 | WB |
| STAT3 | Cell Signalling | 9139 | Mouse | 1/1000 | WB |
| TIMP2 | Abcam | Ab38973 | Rabbit | 1/1000 | WB |
| Vimentin | Dako | M0725 | Mouse | 1/100  1/1000 | IF  WB |
|  |  |  |  |  |  |
| Secondary antibody | **Brand** | **Cat#** | **Origin** | **Dilution** | **Appl*** |
| Alexa Fluor 555 anti-mouse IgG | Invitrogen | A21422 | Goat | 1/500 | IF |
| Alexa Fluor 555 anti-rat IgG | Invitrogen | A21434 | Goat | 1/500 | IF |
| Alexa Fluor 555 anti-rabbit IgG | Invitrogen | A21422 | Goat | 1/500 | IF |
| Alexa Fluor 488 anti-chicken IgG | Invitrogen | A11039 | Goat | 1/500 | IF |
| Alexa Fluor 488 anti-sheep IgG | Invitrogen | A11015 | Goat | 1/500 | IF |
| Alexa Fluor 488 anti-mouse IgG | Invitrogen | A21121 | Goat | 1/500 | IF |
| Alexa Fluor 488 anti-rabbit IgG | Invitrogen | A11034 | Goat | 1/500 | IF |
| Alexa Fluor 488 anti-rat IgG | Invitrogen | A11006 | Goat | 1/500 | IF |
| Alexa Fluor 488 anti-goat IgG | Invitrogen | A-11055 | Donkey | 1/1000 | IF |
| Alexa Fluor 568 anti-goat IgG | Invitrogen | A-21432 | Donkey | 1/1000 | IF |
| Anti-goat IgG HRP conjugate | Merck Millipore | AP180P | Donkey | 1/2500 | WB |
| Anti-mouse IgG HRP conjugate | GE Healthcare | NXA931 | Sheep | 1/2500 | WB |
| Anti-rabbit IgG HRP conjugate | GE Healthcare | NA934V | Donkey | 1/2500 | WB |
| Anti-sheep IgG HRP Conjugate | Invitrogen | 618620 | Rabbit | 1/5000 | WB |
| Supplementary table 3. Primary and secondary antibodies. Appl*: Application*; WB: Western Blot. IF: Immunofluorescence. | | | | | |

| **GENE:** | **MOLECULE:** | **FUNCTION:** |
| --- | --- | --- |
|  |  |  |
| **AMFR** | Autocrine motility factor receptor | Transmembrane receptors, the ligand of which is secreted by the tumour cell itself, the activation of which leads to an increase in the mobility of the tumour cell. |
| **CD44** | CD44 molecule | Cell surface glycoprotein, hyaluronic acid receptors. |
| **CLDN7** | Claudina 7 | Integral membrane protein component of tight junctions. |
| **FKBP4** | FKBP Prolyl Isomerase 4 | Protein of the immunophilinase family |
| **FRK** | Fyn-related kinase | Nuclear tyrosine kinase involved in growth suppression between the G1 and S phases of the cell cycle. |
| **GJA1** | Gap Junction Alpha 1 | Protein component of Gap Junctions |
| **KRT19** | Cytokeratin 19 | Intermediate filament protein responsible for the structural integrity of epithelial cells. |
| **LAMC2** | Gamma 2 laminin subunit | Extracellular matrix glycoproteins, the main constituent of the basement membrane. |
| **MAPK13** | Mitogen-activated protein kinase 13 | It acts as a point of integration for multiple biochemical signals and is involved in various cellular processes such as proliferation. |
| **MUC1** | Mucina 1 | Surface-associated protein, essential for the formation of a protective mucosal barrier on the surface of epithelial cells. |
| **MYOF** | Myoferrin | Plasma and nuclear membrane associated protein. |
| **OVOL2** | Egg Like Zinc Finger 2 | Transcription repressor factor with zinc pretend domains, involved in epithelial development and differentiation. |
| **PGR** | Progesterone receptor | Member of the steroid receptor superfamily. Its presence in breast cancer promotes growth under progesterone stimulation. |
| **PKP3** | Plakophilin 3 | Desmosome protein involved in cell adhesion. |
| **PLXNB1** | Plexin B1 | SEMA4A and 4D receptor, associated with metastasis in breast cancer |
| **PTPN6** | Non-receptor type 6 tyrosine phosphatase protein | Signalling molecule involved in multiple processes such as cell growth, differentiation, mitotic cycle and oncogenic transformation. |
| **RAB17** | Ras-related Rab-17 protein | Small GTP-binding proteins involved in membrane trafficking. |
| **RAB31** | Ras-related Rab-31 protein | Small GTP-binding proteins involved in vesicle orientation. |
| **RET** | Ret Proto-Oncogen | Transmembrane receptor and member of the protein tyrosine kinase family. Involved in cell differentiation, growth, migration and survival. |
| **SEMA3B** | Semaphorin 3B | Axonal guide molecule. It has been described to suppress the progression of breast cancer. |
| **TSPAN13** | Tetraspanin 13 | Cell surface proteins involved in cell development, growth and motility. |
| **UGDH** | UDP-Glucose 6-Dehydrogenase | Biosynthesis of glycosaminoglycans components of the extracellular matrix involved in signal transduction, cell migration, tumourigenesis, and metastasis. |
| **Supplementary Table 4**. Genes differentially expressed between patients with pure DCIS, categorized with high expression and low expression of SEMA3F. These genes have been selected as enriched in the SEMA3F high expression group through Gene Set Enrichment Analysis (GSEA) using the GSE26304 database. All data presented in this table has been sourced from GeneCards (https://www.genecards.org/). | | |
